# Author Correction: Interacting topological quantum chemistry in 2D with many-body real space invariants

**DOI:** 10.1038/s41467-024-47165-z

**Published:** 2024-04-17

**Authors:** Jonah Herzog-Arbeitman, B. Andrei Bernevig, Zhi-Da Song

**Affiliations:** 1 Department of Physics, Princeton University, Princeton, NJ 08544 USA; 2 Donostia International Physics Center, P. Manuel de Lardizabal 4, 20018 Donostia-San Sebastian, Spain; 3 IKERBASQUE, Basque Foundation for Science, Bilbao, Spain; 4 Present Address: International Center for Quantum Materials, School of Physics, Peking University, Beijing, 100871 China; 5 Present Address: Hefei National Laboratory, Hefei, 230088 China; 6 Present Address: Collaborative Innovation Center of Quantum Matter, Beijing, 100871 China

**Keywords:** Topological insulators, Electronic properties and materials

Correction to: *Nature Communications* 10.1038/s41467-024-45395-9, published online 8 February 2024

The original version of this Article omitted the following from the Acknowledgements: “Z.-D. S. was supported by the National Key Research and Development Program of China (No. 2021YFA1401903), National Natural Science Foundation of China (General Program No. 12274005), and Innovation Program for Quantum Science and Technology (No. 2021ZD0302403).”

This has now been corrected in both the PDF and HTML versions of the Article.

The original version of this Article did not contain the present addresses for the author Zhi-Da Song. This has now been corrected in both the PDF and HTML versions of the Article.

