# Supplementary material for: Interacting topological quantum chemistry in 2D with many-body real space invariants
Source: Nat Commun. 2024 Feb 8;15:1171. doi: 10.1038/s41467-024-45395-9 (PMC11258144; doi:10.1038/s41467-024-45395-9)
Supplement: Supplementary file 1 — Supplementary Information [file 41467_2024_45395_MOESM1_ESM.pdf]

Supplementary Information

Jonah Herzog-Arbeitman[1], B. Andrei Bernevig[1,2,3], and Zhi-Da Song[1,4]
[1]*Department of Physics, Princeton University, Princeton, NJ 08544*
[2]*Donostia International Physics Center, P. Manuel de Lardizabal 4, 20018 Donostia-San Sebastian, Spain*
[3]*IKERBASQUE, Basque Foundation for Science, Bilbao, Spain and*
[4]*International Center for Quantum Materials, School of Physics, Peking University, Beijing 100871, China*

I. SUPPLEMENTARY NOTE 1

A. Symmetries in Interacting Hamiltonians

In this Supplementary Note, we lay out our notations for the Hamiltonians, symmetries, and irreducible representations (irreps) defined in this work. Supplementary Tables I and II list the character tables in all point groups (PGs) with and without time-reversal symmetry.

Let $\mathbf{a}_1, \mathbf{a}_2$ be the 2D Bravais lattice vectors, which we normalize for convenience by requiring $\mathbf{a}_1 \times \mathbf{a}_2 = +1$, fixing the area of the unit cell to 1. Greek indices $\alpha = 1, \dots, N_{orb}$ denote the orbitals of the unit cell (i.e. the local Hilbert space dimension). We find it convenient to work in an index convention where $c^\dagger_{\mathbf{R},\alpha}$ is the creation operator in the $\mathbf{R} = r_1\mathbf{a}_1 + r_2\mathbf{a}_2, r_i \in \mathbb{Z}$ unit cell of orbital α, which is located at $\mathbf{R} + \boldsymbol{\delta}_\alpha$. We raise and lower indices on the electron operators to save space in some expressions.

We now discuss crystalline symmetries which form the space group (also called a wallpaper group in two spatial dimensions) G. Comprehensive details of the space group may be found in **??**. In brief, G contains an infinite subgroup composed of the translations T_i and a finite set of generators of rotations and in-plane mirrors which yield the point groups (PGs). In this work, we restrict ourselves to these smymorphic (PG) symmetries. G may also contain time-reversal $\mathcal{T}$, but we do not consider magnetic space groups explicitly here. However, one can extend our classification to this case.

A space group symmetry $g \in G$ is defined by its action on the election operators which reads

$$g^\dagger c_{\mathbf{R},\alpha} g = \sum_\beta D[g]^\alpha_\beta c_{g(\mathbf{R}+\boldsymbol{\delta}_\alpha)-\boldsymbol{\delta}_\beta,\beta} \ . \tag{1}$$

where $D[g]^\alpha_\beta$, the $N_{orb}\times N_{orb}$ representation matrix of g on the orbitals, is nonzero only when $g\boldsymbol{\delta}_\alpha = \boldsymbol{\delta}_\beta \mod \mathbf{a}_i$. Hence $g(\mathbf{R}+\boldsymbol{\delta}_\alpha)-\boldsymbol{\delta}_\beta$ is a lattice vector and Eq. 1 is well-defined. Spinless representations describe systems without spin-orbit coupling (SOC) and have $D[C_n]^n = D[M]^2 = +1$ and spinful systems with SOC satisfy $D[C_n]^n = D[M]^2 = -1$, where C_n is an n-fold rotation and M is a mirror. In groups with time-reversal $\mathcal{T}$, spinless particles have $\mathcal{T}^2 = +1$ and spinful particles have $\mathcal{T}^2 = -1$. The representation of $\mathcal{T}$ on the orbitals is denoted $D[\mathcal{T}]K$ where K is the complex conjugation operator, and hence $D[\mathcal{T}]D^*[\mathcal{T}] = \pm 1$ for spinless/spinful particles. The irreps in all 2D PGs with and without SOC, with and without time-reversal are shown in Supplementary Tables I and II. They can also be found, with full group theory data, on the Bilbao Crystallographic Server[1,2] as the $k_z = 0$ slice of the 3D layer groups.

We can now write down a Hamiltonian H with arbitrary interactions that preserve the space group symmetries. We decompose $H = \sum_q H_q$ where H_q is a "q-body" term. We call H interacting if it contains 2-body or higher terms. Explicitly, the q-body term is written

$$H_q = \sum_{\mathbf{R}'_1\dots\mathbf{R}'_q,\mathbf{R}_1\dots\mathbf{R}_1} t^{\alpha_1\dots\alpha_q}_{\beta_1\dots\beta_q}(\{\mathbf{R}',\mathbf{R}\}) c^\dagger_{\mathbf{R}'_1,\alpha_1}\dots c^\dagger_{\mathbf{R}'_q,\alpha_q} c_{\mathbf{R}_1,\beta_1}\dots c_{\mathbf{R}_q,\beta_q} \tag{2}$$

where we have summed over the repeated greek indices. The tensor t obeys

$$\begin{aligned} \text{fermion anti-symmetry}: &\ t^{\alpha_2\alpha_1\dots\alpha_q}_{\beta_1\dots\beta_q}(\mathbf{R}'_2\mathbf{R}'_1,\dots) = -t^{\alpha_1\alpha_2\dots\alpha_q}_{\beta_1\dots\beta_q}(\mathbf{R}'_1\mathbf{R}'_2\dots), \\ \text{Hermiticity}: &\ \left(t^{\alpha_1\dots\alpha_q}_{\beta_1\dots\beta_q}(\{\mathbf{R}',\mathbf{R}\})\right)^* = t^{\beta_1\dots\beta_q}_{\alpha_1\dots\alpha_q}(\{\mathbf{R},\mathbf{R}'\}) \ . \end{aligned} \tag{3}$$

By imposing translation invariance, we find

$$t^{\alpha_1\dots\alpha_q}_{\beta_1\dots\beta_q}(\{\mathbf{R}',\mathbf{R}\}) = t^{\alpha_1\dots\alpha_q}_{\beta_1\dots\beta_q}(\{\mathbf{R}'+\mathbf{a}_i,\mathbf{R}+\mathbf{a}_i\}) \ . \tag{4}$$

Continuing to impose that $g \in G$ is a symmetry of H_q for general elements of the space group, we require

$$\begin{aligned} g^\dagger H_q g &= \sum_{\{\mathbf{R},\mathbf{R}'\}} t^{\alpha_1\ldots\alpha_q}_{\beta_1\ldots\beta_q}(\{\mathbf{R}',\mathbf{R}\}) g^\dagger c^\dagger_{\mathbf{R}'_1,\alpha_1} g \ldots g^\dagger c^\dagger_{\mathbf{R}'_q,\alpha_q} g g^\dagger c^{\beta_1}_{\mathbf{R}_1} g \ldots g^\dagger c^{\beta_q}_{\mathbf{R}_q} g \\ &= \sum_{\{\mathbf{R},\mathbf{R}'\}} (D^\dagger[g]^{\alpha'_1}_{\alpha_1} \ldots D^\dagger[g]^{\alpha'_q}_{\alpha_q}) t^{\alpha_1\ldots\alpha_q}_{\beta_1\ldots\beta_q}(\{g^{-1}(\mathbf{R}'+\boldsymbol{\delta}_{\alpha'}) - \boldsymbol{\delta}_\alpha, g^{-1}(\mathbf{R}+\boldsymbol{\delta}_{\beta'}) - \boldsymbol{\delta}_\beta\})(D[g]^{\beta_1}_{\beta'_1} \ldots) c^\dagger_{\mathbf{R}'_1,\alpha'_1} \ldots c^\dagger_{\mathbf{R}'_q,\alpha'_q} c^{\beta'_1}_{\mathbf{R}_1} \ldots c^{\beta'_q}_{\mathbf{R}_q} \end{aligned} \tag{5}$$

The $\alpha_1,\ldots,\alpha_q$ and $\beta_1,\ldots,\beta_q$ indices are fully anti-symmetric because they contract with the fully anti-symmetric $t^{\alpha_1,\ldots,\alpha_q}_{\beta_1,\ldots,\beta_q}$ tensor, and the $\alpha'_1,\ldots,\alpha'_q$ and $\beta'_1,\ldots,\beta'_q$ indices are fully anti-symmetric because they contract with the electron operators. Now using $gH_qg^\dagger = H_q$, we obtain

$$(D^\dagger[g]^{\alpha'_1}_{\alpha_1} \ldots D^\dagger[g]^{\alpha'_q}_{\alpha_q}) t^{\alpha_1\ldots\alpha_q}_{\beta_1\ldots\beta_q}(\{\mathbf{R}'+\boldsymbol{\delta}_{\alpha'}) - \boldsymbol{\delta}_\alpha, g^{-1}(\mathbf{R}+\boldsymbol{\delta}_{\beta'}) - \boldsymbol{\delta}_\beta\}\}(D[g]^{\beta_1}_{\beta'_1} \ldots D[g]^{\beta_q}_{\beta'_q}) = t^{\alpha'_1\ldots\alpha'_q}_{\beta'_1\ldots\beta'_q}(\{\mathbf{R}',\mathbf{R}\}) \tag{6}$$

so t transforms in the representation $\bigwedge_{i=1}^q D[g] = D[g] \wedge \cdots \wedge D[g]$. Here $\wedge$ denotes the exterior (anti-symmetric) product which anti-symmetrizes $D^\dagger[g]^{\alpha'_1}_{\alpha_1} \ldots D^\dagger[g]^{\alpha'_q}_{\alpha_q}$ in its top and bottom indices. Time reversal behaves similarly. Repeating the calculation, we find

$$(D^\dagger[\mathcal{T}]^{\alpha'_1}_{\alpha_1} \ldots D^\dagger[\mathcal{T}]^{\alpha'_q}_{\alpha_q}) t^{*\alpha_1\ldots\alpha_q}_{\beta_1\ldots\beta_q}(\{\mathbf{R}',\mathbf{R}\})(D[\mathcal{T}]^{\beta_1}_{\beta'_1} \ldots D[\mathcal{T}]^{\beta_q}_{\beta'_q}) = t^{\alpha'_1\ldots\alpha'_q}_{\beta'_1\ldots\beta'_q}(\{\mathbf{R}',\mathbf{R}\}) \,. \tag{7}$$

Finally, we remark that our expression for H in Eq. 2 is particle-number preserving. By construction, H commutes with the total $U(1)$ charge operator

$$U(\theta) = e^{i\theta\hat{N}}, \quad \hat{N} = \sum_{\mathbf{R}} c^\dagger_{\mathbf{R},\alpha} c_{\mathbf{R},\alpha} \tag{8}$$

for all θ, and we have set the electron charge to 1. $U(\theta)$ generates the $U(1)$ global symmetry and allows us to fix the total $U(1)$ particle number to N. The filling in the thermodynamic limit is $\nu = N/A = N_{occ}/N_{orb}$ where A is the number of unit cells, N_{orb} is the number of orbitals per unit cell, and N_{occ} is the number of electrons per unit cell.

PG 2	1	2
A	1	1
B	1	-1
${}^2\overline{E}$	1	$-i$
${}^1\overline{E}$	1	i

PG m		1	m
A'	A'	1	1
A''	A''	1	-1
${}^2E_{1/2}$	${}^2\overline{E}$	1	$-i$
${}^1E_{1/2}$	${}^1\overline{E}$	1	i

PG $2mm$		1	2	m_{100}	m_{010}
A_1	A_1	1	1	1	1
A_2	A_2	1	1	-1	-1
B_1	B_1	1	-1	-1	1
B_2	B_2	1	-1	1	-1
$E_{1/2}$	$\overline{E}$	2	0	0	0

PG 4		1	4^+	2	4^-
A	A	1	1	1	1
B	B	1	-1	1	-1
${}^1E$	${}^1E$	1	$-i$	-1	i
${}^2E$	${}^2E$	1	i	-1	$-i$
${}^1E_{1/2}$	${}^2\overline{E}_1$	1	$e^{-i\frac{\pi}{4}}$	$-i$	$e^{i\frac{\pi}{4}}$
${}^2E_{1/2}$	${}^1\overline{E}_1$	1	$e^{i\frac{\pi}{4}}$	i	$e^{-i\frac{\pi}{4}}$
${}^1E_{3/2}$	${}^2\overline{E}_2$	1	$e^{i\frac{3\pi}{4}}$	$-i$	$e^{-i\frac{3\pi}{4}}$
${}^2E_{3/2}$	${}^1\overline{E}_2$	1	$e^{-i\frac{3\pi}{4}}$	i	$e^{i\frac{3\pi}{4}}$

PG $4mm$		1	$\{4^+, 4^-\}$	2	$\{m_{010}, m_{100}\}$	$\{m_{110}, m_{1-10}\}$
A_1	A_1	1	1	1	1	1
A_2	A_2	1	1	1	-1	-1
B_1	B_1	1	-1	1	1	-1
B_2	B_2	1	-1	1	-1	1
E	E	2	0	-2	0	0
$E_{1/2}$	$\overline{E}_1$	2	$\sqrt{2}$	0	0	0
$E_{3/2}$	$\overline{E}_2$	2	$-\sqrt{2}$	0	0	0

PG 3		1	3^+	3^-
A	A	1	1	1
${}^2E$	${}^2E$	1	$e^{i\frac{2\pi}{3}}$	$e^{-i\frac{2\pi}{3}}$
${}^1E$	${}^1E$	1	$e^{-i\frac{2\pi}{3}}$	$e^{i\frac{2\pi}{3}}$
$A_{3/2}$	$\overline{E}$	1	-1	-1
${}^2E_{1/2}$	${}^1\overline{E}$	1	$e^{-i\frac{\pi}{3}}$	$e^{i\frac{\pi}{3}}$
${}^1E_{1/2}$	${}^2\overline{E}$	1	$e^{i\frac{\pi}{3}}$	$e^{-i\frac{\pi}{3}}$

PG 6		1	6^+	3^+	2	3^-	6^-
A	A	1	1	1	1	1	1
B	B	1	-1	1	-1	1	-1
${}^1E_1$	${}^1E_2$	1	$e^{-i\frac{\pi}{3}}$	$e^{-i\frac{2\pi}{3}}$	-1	$e^{i\frac{2\pi}{3}}$	$e^{i\frac{\pi}{3}}$
${}^2E_1$	${}^2E_2$	1	$e^{i\frac{\pi}{3}}$	$e^{i\frac{2\pi}{3}}$	-1	$e^{-i\frac{2\pi}{3}}$	$e^{-i\frac{\pi}{3}}$
${}^1E_2$	${}^1E_1$	1	$e^{i\frac{2\pi}{3}}$	$e^{i\frac{4\pi}{3}}$	1	$e^{i\frac{2\pi}{3}}$	$e^{i\frac{4\pi}{3}}$
${}^2E_2$	${}^2E_1$	1	$e^{-i\frac{2\pi}{3}}$	$e^{-i\frac{4\pi}{3}}$	1	$e^{-i\frac{2\pi}{3}}$	$e^{-i\frac{4\pi}{3}}$
${}^1E_{1/2}$	${}^2\overline{E}_3$	1	$e^{i\frac{\pi}{6}}$	$e^{i\frac{\pi}{3}}$	i	$e^{-i\frac{\pi}{3}}$	$e^{-i\frac{\pi}{6}}$
${}^2E_{1/2}$	${}^1\overline{E}_3$	1	$e^{-i\frac{\pi}{6}}$	$e^{-i\frac{\pi}{3}}$	$-i$	$e^{i\frac{\pi}{3}}$	$e^{i\frac{\pi}{6}}$
${}^1E_{3/2}$	${}^2\overline{E}_1$	1	$-i$	-1	i	-1	i
${}^2E_{3/2}$	${}^1\overline{E}_1$	1	i	-1	$-i$	-1	$-i$
${}^1E_{5/2}$	${}^2\overline{E}_2$	1	$-e^{i\frac{\pi}{6}}$	$e^{i\frac{\pi}{3}}$	$-i$	$e^{-i\frac{\pi}{3}}$	$-e^{-i\frac{\pi}{6}}$
${}^2E_{5/2}$	${}^1\overline{E}_2$	1	$-e^{-i\frac{\pi}{6}}$	$e^{-i\frac{\pi}{3}}$	i	$e^{i\frac{\pi}{3}}$	$-e^{i\frac{\pi}{6}}$

PG $3m$		1	$\{3^+, 3^-\}$	$\{m_{120}, m_{210}, m_{1-10}\}$
A_1	A_1	1	1	1
A_2	A_2	1	1	-1
E	E	2	-1	0
$E_{1/2}$	$\overline{E}_1$	2	1	0
${}^1E_{3/2}$	${}^1\overline{E}$	1	-1	i
${}^2E_{3/2}$	${}^2\overline{E}$	1	-1	$-i$

PG $6mm$		1	$\{6^+, 6^-\}$	$\{3^+, 3^-\}$	2	$\{m_{100}, m_{010}, m_{110}\}$	$\{m_{120}, m_{210}, m_{1-10}\}$
A_1	A_1	1	1	1	1	1	1
A_2	A_2	1	1	1	1	-1	-1
B_1	B_1	1	-1	1	-1	-1	1
B_2	B_2	1	-1	1	-1	1	-1
E_1	E_1	2	1	-1	-2	0	0
E_2	E_2	2	-1	-1	2	0	0
$E_{1/2}$	$\overline{E}_1$	2	$\sqrt{3}$	1	0	0	0
$E_{3/2}$	$\overline{E}_3$	2	0	-2	0	0	0
$E_{5/2}$	$\overline{E}_2$	2	$-\sqrt{3}$	1	0	0	0

TABLE I. Supplementary Table 1. The character tables of 2D magnetic PGs. The irrep names are shown in the first column using the Altmann-Herzig notation and second columns using the Bilbao Crystallographic notation. However, in $p6$, the notation differs from the BCS but is consistent with Ref[3]. In each table, the linear irreps above the second horizontal line are the single-valued irreps (no-SOC), and the projective irreps below the second horizontal line are the double-valued irreps (SOC).

PG $21'$		1	2
A		1	1
B		1	-1
$^1\overline{E}{}^2\overline{E}$		2	0

PG $m1'$		1	m
A'	A'	1	1
A''	A''	1	-1
$^2E_{1/2}{}^1E_{1/2}$	$^2\overline{E}{}^1\overline{E}$	2	0

PG $2mm1'$		1	2	m_{100}	m_{010}
A_1	A_1	1	1	1	1
A_2	A_2	1	1	-1	-1
B_1	B_1	1	-1	-1	1
B_2	B_2	1	-1	1	-1
$E_{1/2}$	$\overline{E}$	2	0	0	0

PG $41'$		1	4^+	2	4^-
A	A	1	1	1	1
B	B	1	-1	1	-1
$^1E{}^2E$	$^1E{}^2E$	2	0	-2	0
$^1E_{1/2}{}^2E_{1/2}$	$^1\overline{E}_1{}^2\overline{E}_1$	2	$\sqrt{2}$	0	$\sqrt{2}$
$^1E_{3/2}{}^2E_{3/2}$	$^1\overline{E}_2{}^2\overline{E}_2$	2	$-\sqrt{2}$	0	$-\sqrt{2}$

PG $4mm1'$		1	$\{4^+,4^-\}$	2	$\{m_{010},m_{100}\}$	$\{m_{110},m_{1-10}\}$
A_1	A_1	1	1	1	1	1
A_2	A_2	1	1	1	-1	-1
B_1	B_1	1	-1	1	1	-1
B_2	B_2	1	-1	1	-1	1
E	E	2	0	-2	0	0
$E_{1/2}$	$\overline{E}_1$	2	$\sqrt{2}$	0	0	0
$E_{3/2}$	$\overline{E}_2$	2	$-\sqrt{2}$	0	0	0

PG $31'$		1	3^+	3^-
A	A	1	1	1
$^1E{}^2E$	$^1E{}^2E$	2	-1	-1
$A_{3/2}A_{3/2}$	$\overline{E}\overline{E}$	2	-2	-2
$^2E_{1/2}{}^1E_{1/2}$	$^1\overline{E}{}^2\overline{E}$	2	1	1

PG $3m1'$		1	$\{3^+,3^-\}$	$\{m_{120},m_{210},m_{1-10}\}$
A_1	A_1	1	1	1
A_2	A_2	1	1	-1
E	E	2	-1	0
$E_{1/2}$	$\overline{E}_1$	2	1	0
$^1E_{3/2}{}^2E_{3/2}$	$^1\overline{E}{}^2\overline{E}$	2	-2	0

PG $61'$		1	6^+	3^+	2	3^-	6^-
A	A	1	1	1	1	1	1
B	B	1	-1	1	-1	1	-1
$^1E_1{}^2E_1$	$^1E_2{}^2E_2$	2	1	-1	-2	-1	1
$^1E_2{}^2E_2$	$^1E_1{}^2E_1$	2	-1	-1	2	-1	-1
$^1E_{1/2}{}^2E_{1/2}$	$^1\overline{E}_3{}^2\overline{E}_3$	2	$\sqrt{3}$	1	0	1	$\sqrt{3}$
$^1E_{3/2}{}^2E_{3/2}$	$^1\overline{E}_1{}^2\overline{E}_1$	2	0	-2	0	-2	0
$^1E_{5/2}{}^2E_{5/2}$	$^1\overline{E}_2{}^2\overline{E}_2$	2	$-\sqrt{3}$	1	0	1	$-\sqrt{3}$

PG $6mm1'$		1	$\{6^+,6^-\}$	$\{3^+,3^-\}$	2	$\{m_{100},m_{010},m_{110}\}$	$\{m_{120},m_{210},m_{1-10}\}$
A_1	A_1	1	1	1	1	1	1
A_2	A_2	1	1	1	1	-1	-1
B_1	B_1	1	-1	1	-1	-1	1
B_2	B_2	1	-1	1	-1	1	-1
E_1	E_1	2	1	-1	-2	0	0
E_2	E_2	2	-1	-1	2	0	0
$E_{1/2}$	$\overline{E}_1$	2	$\sqrt{3}$	1	0	0	0
$E_{3/2}$	$\overline{E}_3$	2	0	-2	0	0	0
$E_{5/2}$	$\overline{E}_2$	2	$-\sqrt{3}$	1	0	0	0

TABLE II. Supplementary Table 2. The character tables of 2D PGs with time-reversal (sometimes called the gray groups). Here and throughout $1'$ denotes $\mathcal{T}$ as a group element. The irrep names are shown in the first column using the Altmann-Herzig notation and second columns using the Bilbao Crystallographic notation. In each table, the irreps above the second horizontal line are the single-valued irreps (no-SOC), and the irreps below the second horizontal line are the double-valued irreps (SOC). The traces of the anti-unitary operators are not shown because they are not invariant under unitary transforms.

II. SUPPLEMENTARY NOTE 2

A. Many-Body Atomic Limits and Construction of RSIs

In this Supplementary Note, we construct many-body local Real Space Invariants (RSIs). First Supplementary Note II B gives a simple example of how single-particle RSIs breaks down with interactions. Supplementary Note II C then defines the many-body atomic limit states and gives explicit expressions for the many-body local RSIs of rotation groups. Supplementary Note II D generalizes these results to all 2D point groups with mirrors, time-reversal, and spin-orbit coupling. Lastly, Supplementary Note II E evaluates the many-body local RSIs on product states to give expressions in terms of irrep multiplicities and single-particle RSIs.

B. 1D Many-Body RSI example

?? obtained the single-particle Real Space Invariants (RSIs) as adiabatic invariants in non-interacting Hamiltonians. States with different single-particle RSIs cannot be connected without a gap closing in non-interacting Hamiltonians. We give a minimal example of the breakdown of these single-particle RSIs when interactions are added by showing that two states with different single-particle RSIs can be brought into superposition by interaction terms.

Let us consider a 1D system with inversion (C_2) symmetry. We propose a three site system with open boundaries. The site $R=0$ has two s orbitals denoted s, s', and the sites at $R=1,-1$ have two p orbitals each denoted p, p'. Inversion $\mathcal{I}$ takes $R \to -R$. We set the non-interacting Hamiltonian to be

$$H_0 = -\frac{t}{2}(c^\dagger_{0,s}c_{0,s} + c^\dagger_{0,s'}c_{0,s'}) + \frac{t}{2}(c^\dagger_{-1,p}c_{-1,p} + c^\dagger_{-1,p'}c_{-1,p'} + c^\dagger_{1,p}c_{1,p} + c^\dagger_{1,p'}c_{1,p'}), \quad t>0 \tag{9}$$

so the s orbitals are at lower energy than the p orbitals. The groundstate of H_0 at filling 2 is given by $|GS\rangle = c^\dagger_{0,s}c^\dagger_{0,s'}|0\rangle$. We add a pair-hopping interaction term in the form

$$H_{int} = \frac{1}{\sqrt{2}}U(c^\dagger_{1,p}c^\dagger_{1,p'}c_{0,s}c_{0,s'} + c^\dagger_{-1,p}c^\dagger_{-1,p'}c_{0,s}c_{0,s'}) + h.c. \tag{10}$$

which respects inversion symmetry, using $\mathcal{I}c^\dagger_{r,\alpha}\mathcal{I}^\dagger = \pm c^\dagger_{-r,\alpha}$ for $\alpha = s/p$. Intuitively, H_{int} connects the states $c^\dagger_{0,s}c^\dagger_{0,s'}|0\rangle$ and $\frac{1}{\sqrt{2}}(c^\dagger_{1,p}c^\dagger_{1,p'} + c^\dagger_{-1,p}c^\dagger_{-1,p'})|0\rangle$. This would be impossible at the single-particle level. Alternatively, we can diagonalize $H_0 + H_{int}$ to obtain explicit energies and states. There are 6 orbitals in the model, so filling 2 gives a 15-dimensional Hilbert space. Inversion symmetry splits the Hilbert space into 7 even parity states and 8 odd parity states. A basis for the even parity states is

$$|i\rangle = c^\dagger_{0,s}c^\dagger_{0,s'}|0\rangle\,,\ w^\dagger_{0,s}w^\dagger_{0,s'}|0\rangle\,,\ w^\dagger_{0,p}w^\dagger_{0,p'}|0\rangle\,,\ c^\dagger_{0,s}w^\dagger_{0,s}|0\rangle\,,\ c^\dagger_{0,s'}w^\dagger_{0,s}|0\rangle\,,\ c^\dagger_{0,s}w^\dagger_{0,s'}|0\rangle\,,\ c^\dagger_{0,s'}w^\dagger_{0,s'}|0\rangle \tag{11}$$

where we introduced the Wannier functions

$$\begin{aligned} w^\dagger_{0,p} &= \frac{1}{\sqrt{2}}(c^\dagger_{1,p} + c^\dagger_{-1,p}), \qquad w^\dagger_{0,s} = \frac{1}{\sqrt{2}}(c^\dagger_{1,p} - c^\dagger_{-1,p}) \\ w^\dagger_{0,p'} &= \frac{1}{\sqrt{2}}(c^\dagger_{1,p'} + c^\dagger_{-1,p'}), \qquad w^\dagger_{0,s'} = \frac{1}{\sqrt{2}}(c^\dagger_{1,p'} - c^\dagger_{-1,p'}) \end{aligned} \tag{12}$$

which obey, for instance, $\mathcal{I}w^\dagger_{0,s}\mathcal{I} = w^\dagger_{0,s}$ and $\mathcal{I}w^\dagger_{0,p}\mathcal{I} = -w^\dagger_{0,p}$. The Wannier states are centered at $R=0$, and hence behave as s/p orbitals at the $R=0$ site under inversion. The states in Eq. 12 are simply the induced representations of $G_0 = \{1, \mathcal{I}\}$ formed by the p orbitals at $R = \pm 1$. One can also check that this Wannier basis is orthogonal, e.g. $\{w^\dagger_{0,p}, w^\dagger_{0,s}\} = 0$.

In the order of Eq. 11, the Hamiltonian can be written (in the even parity sector) as

$$\langle i|H_0 + H_{int}|j\rangle = \begin{pmatrix} -t & U/\sqrt{2} & U/\sqrt{2} & & & & \\ U/\sqrt{2} & t & 0 & & & & \\ U/\sqrt{2} & 0 & t & & & & \\ & & & 0 & & & \\ & & & & 0 & & \\ & & & & & 0 & \\ & & & & & & 0 \end{pmatrix}_{ij} \tag{13}$$

whose spectrum is $\pm\sqrt{U^2+t^2}, t, 0, 0, 0, 0,$. The groundstate is at energy $-\sqrt{U^2+t^2}$ and remains gapped for all U. At $U=0$, the groundstate is $c^\dagger_{0,s}c^\dagger_{0,s'}|0\rangle$, but interactions yield the entangled groundstate wavefunction

$$|GS(U)\rangle \propto \sqrt{2}(\sqrt{U^2+t^2}-t)c^\dagger_{0,s}c^\dagger_{0,s'}|0\rangle + U\left(c^\dagger_{1,p}c^\dagger_{1,p'} + c^\dagger_{-1,p}c^\dagger_{-1,p'}\right)|0\rangle \tag{14}$$

which we left normalized, and used the simple identity

$$\begin{aligned} w^\dagger_{0,s}w^\dagger_{0,s'} + w^\dagger_{0,p}w^\dagger_{0,p'} &= \frac{1}{\sqrt{2}}(c^\dagger_{1,p} - c^\dagger_{-1,p})\frac{1}{\sqrt{2}}(c^\dagger_{1,p'} - c^\dagger_{-1,p'}) + \frac{1}{\sqrt{2}}(c^\dagger_{1,p} + c^\dagger_{-1,p})\frac{1}{\sqrt{2}}(c^\dagger_{1,p'} + c^\dagger_{-1,p'}) \\ &= c^\dagger_{1,p}c^\dagger_{1,p'} + c^\dagger_{-1,p}c^\dagger_{-1,p'} \ . \end{aligned} \tag{15}$$

We now show that $|GS(U)\rangle$ has a well-defined many-body RSI. The two-particle product states $c^\dagger_{0,s}c^\dagger_{0,s'}$, $w^\dagger_{0,s}w^\dagger_{0,s'}$, and $w^\dagger_{0,p}w^\dagger_{0,p'}$ each have a definite single-particle RSI (they are non-interacting Wannier product states), but their single-particle RSIs are different. Because $w^\dagger_{0,s}w^\dagger_{0,s'}$ and $w^\dagger_{0,p}w^\dagger_{0,p'}$ have the same *many-body* RSI, they can be brought into superposition, necessarily creating entanglement. To be explicit, the single-particle RSI $\delta = m(s) - m(p)$ and many-body RSI $\Delta = \delta \mod 4$ of the states are

$$\begin{aligned} c^\dagger_{0,s}c^\dagger_{0,s'}, w^\dagger_{0,s}w^\dagger_{0,s'}: &\qquad \delta = 2, \quad \Delta = 2 \mod 4, \\ w^\dagger_{0,p}w^\dagger_{0,p'}: &\qquad \delta = -2, \quad \Delta = 2 \mod 4 \ . \end{aligned} \tag{16}$$

The fact that the two states have different single-particle RSIs means they are adiabatically distinct without interactions. But, because they have the same many-body RSIs, these two states can be adiabatically connected by interactions. As such, the correlated state $|GS(U)\rangle$ has a well-defined many-body RSI given by $\Delta = 2 \mod 4$. In the following section Supplementary Note II C, we give a rigorous argument for the definition of many-body RSIs in 2D PGs.

C. Defining Many-Body RSIs in the Atomic Limit

We will now construct many-body local RSIs at a fixed Wyckoff position $\mathbf{x}$ in 2D many-body atomic states protected by the symmetries of a point group $G_{\mathbf{x}}$. Our construction proceeds as follows. First we define a class of many-body atomic *limit* states with zero correlation length. We then show that, after imposing a cutoff to create open boundary conditions, there is a natural set of quantum numbers protected by $G_{\mathbf{x}}$ which is invariant as the cutoff is changed in a symmetry-preserving fashion. We identity these invariants as many-body local RSIs and we show that they remain adiabatically well-defined beyond the zero correlation length limit. Since the cutoff can be taken to infinity, the many-body local RSIs remain well-defined as the open boundary conditions approach infinite boundary conditions, where we can simultaneously define many-body local RSIs at each Wyckoff position.

Recall that the non-interacting atomic limit is defined by taking the lattice constant to infinity so that all hoppings in the Hamiltonian go to zero (but orbitals on the same site may still be connected by single-particle terms or interactions). The groundstate of such a system is a product state of occupied local orbitals. Our generalization of atomic limits to interacting Hamiltonians is analogous. We consider a general lattice with N_{orb} electron orbitals per unit cell located at positions $\mathbf{r}_\alpha \in \{\mathbf{r}\}$, $\alpha = 1, \ldots, N_{orb}$. Denote the number of orbitals at position $\mathbf{r}$ to be $n_{\mathbf{r}}$. An interacting Hamiltonian in the atomic limit only contains terms that are totally local in $\mathbf{r}$, e.g. onsite non-interacting

potentials $H_{\mathbf{R},\mathbf{r}} \sim c^\dagger_{\mathbf{R},\alpha} c_{\mathbf{R},\beta}$ or onsite interactions $H_{\mathbf{R},\mathbf{r}} \sim c^\dagger_{\mathbf{R},\alpha} c^\dagger_{\mathbf{R},\alpha'} c_{\mathbf{R},\beta} c_{\mathbf{R},\beta'}$ and higher-body contact terms for $\mathbf{r}_\alpha = \mathbf{r}_{\alpha'} = \mathbf{r}_\beta = \mathbf{r}_{\beta'}$. Such a Hamiltonian can be written

$$H_{AL} = \bigoplus_{\mathbf{R}} \bigoplus_{\mathbf{r}\in\{\mathbf{r}\}} H_{\mathbf{R},\mathbf{r}} = \bigoplus_{\mathbf{R}} \bigoplus_{\mathbf{r}\in\{\mathbf{r}\}} T^\dagger_{\mathbf{R}} H_{\mathbf{r}} T_{\mathbf{R}} \tag{17}$$

where we imposed translation invariance in the second equality. Each $H_{\mathbf{r}}$ acts on a strictly local Hilbert space whose Fock space is $2^{n_{\mathbf{r}}}$ dimensional. Because $H_{\mathbf{r}}$ is strictly local, $[H_{\mathbf{r}}, T^\dagger_{\mathbf{R}} H_{\mathbf{r}'} T_{\mathbf{R}}] = 0$ for all $\mathbf{r}, \mathbf{r}', \mathbf{R}$. Thus H_{AL} is composed of commuting terms (different sites are decoupled) and its ground state at fixed density $\nu = N_{occ}/N_{orb},\ N_{occ} \in \mathbb{N}$ can be written

$$|GS\rangle = \prod_{\mathbf{R},\mathbf{r}\in\{\mathbf{r}\}} T^\dagger_{\mathbf{R}} \mathcal{O}_{\mathbf{r}} T_{\mathbf{R}}\ |0\rangle, \qquad [\hat{N}, \prod_{\mathbf{r}\in\{\mathbf{r}\}} \mathcal{O}_{\mathbf{r}}] = N_{occ} \prod_{\mathbf{r}\in\{\mathbf{r}\}} \mathcal{O}_{\mathbf{r}} \tag{18}$$

where each $\mathcal{O}^\dagger_{\mathbf{r}}$ creates a groundstate of $H_{\mathbf{r}}$ and $\hat{N}$ is the number operator. One can think of $H_{\mathbf{r}}$ as being a quantum dot Hamiltonian, and $\mathcal{O}^\dagger_{\mathbf{r}} |0\rangle$ as the groundstate of the quantum dot.

To move away from this strict atomic limit, hopping terms and/or interactions coupling different sites can be added to H_{AL}. As long as the many-body gap does not close, we say that the ground state is a many-body atomic phase since, by construction, it is adiabatically connected to an atomic limit. Note that an obstructed atomic limit[3] is not a trivial atomic limit as defined here: there is an obstruction to deforming it into a zero correlation length state. For example, the dimerized limit of the SSH chain requires strong inter-site hoppings, and cannot be connected to the trivial atomic limit. Indeed, a trivial atomic limit is spatially decoupled and so has no corner or edge states on open boundary conditions.

We now define many-body local RSIs on the many-body atomic limit states Eq. 18. Recall that non-interacting local RSIs[3] are adiabatic invariants (they do not change value unless a single-particle gap closes) protected by point group symmetries, and are defined in single-particle states at fixed filling. We now explicitly show the existence of symmetry-protected adiabatic invariants which are the quantum numbers of certain symmetry operators in *many-body* atomic limit groundstates (Eq. 18). We call these quantum numbers many-body local RSIs.

A key part of our construction is to define the many-body local RSIs on open boundary conditions which break the space group G to $G_{\mathbf{x}}$. This serves to show that they are local (since they only depend on the groundstate within an arbitrary range of $\mathbf{x}$ set by a cutoff) and are protected only by the point group symmetries of $G_{\mathbf{x}}$. However, it is then crucial to show that the many-body local RSIs do not depend on the choice of cutoff. This ensures that the many-body local RSIs are well-defined invariants of the thermodynamic groundstate Eq. 18 since the spatial cutoff can be sent to infinity. They then serve to define invariants on infinite boundary conditions such that the gap is the thermodynamic gap. Define the open boundary conditions by

$$H_{AL,R,\mathbf{x}} = \bigoplus_{|\mathbf{R}+\mathbf{r}-\mathbf{x}|<R} H_{\mathbf{R},\mathbf{r}} \tag{19}$$

for a Wyckoff position $\mathbf{x}$ and cutoff R (see Supplementary Figure 1a). The choice of a circular cutoff is just for convenience: the following argument hold for any cutoff that preserves $G_{\mathbf{x}}$. At cutoff R, the total electron number N_R is given by $[N, \mathcal{O}_{GS,R}] = N_R \mathcal{O}_{GS,R}$ where $\mathcal{O}_{GS,R} = \prod_{|\mathbf{r}-\mathbf{x}|<R} \mathcal{O}_{\mathbf{r}}$. As the cutoff R is taken to infinity, the filling approaches the thermodynamic filling $\nu = N_{occ}/N_{orb}$.

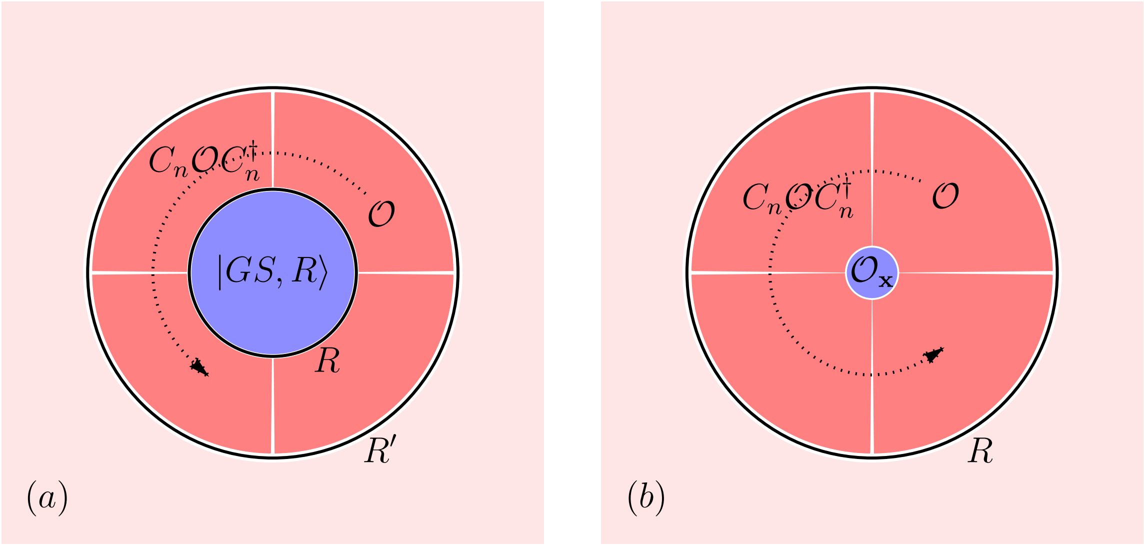

FIG. 1. Supplementary Figure 1. (a) We depict the groundstate $|GS,R\rangle$ on OBCs and the additional symmetry-related operators which are included upon expanding the cutoff to R' (see Eq. 21). The many-body local RSIs are invariant under the expansion. (b) Given a fixed cutoff R, all operators inside of R but not at the C_n-invariant point $\mathbf{x}$ are symmetry-related and so do not contribute to the many-body local RSI (see Eq. 26). Only the operator $O_\mathbf{x}$ at $\mathbf{x}$ transforms locally under $\mathbf{x}$. Its quantum numbers determine the many-body local RSI.

For each cutoff R, denote the ground state of $H_{AL,R,\mathbf{x}}$ by $|GS,R\rangle$ (we omit $\mathbf{x}$ henceforth because it is fixed throughout the calculation). It is important that $|GS,R\rangle$ is non-degenerate and gapped for all R at the exact filling. It is natural to require these two properties because a many-body atomic phase has no edge states so the ground state is unique and is an insulator, so there is a many-body gap. Note that non-degeneracy excludes single-particle obstructed atomic or fragile topological phase (to be addressed in Supplementary Note III C), and spontaneously broken symmetries like a charge density wave. We now construct many-body local RSIs of $H_{AL,R,\mathbf{x}}$ at a Wyckoff position $\mathbf{x}$ protected by the point group symmetries of $G_\mathbf{x}$. We will then use an adiabaticity argument to show that the many-body local RSIs are still well-defined in a many-body atomic phase once weak hoppings and off-site interactions are introduced. Because $|GS,R\rangle$ is non-degenerate (it is a strict atomic limit state), it transforms in a 1D irrep of $G_\mathbf{x}$. Thus $|GS,R\rangle$ has the quantum numbers

$$\begin{aligned} \hat{N}\,|GS,R\rangle &= N\,|GS,R\rangle\,, \\ g\,|GS,R\rangle &= e^{i\lambda[g]}\,|GS,R\rangle\,, \qquad g\in G_\mathbf{x}\,. \end{aligned} \tag{20}$$

However, the quantum numbers $N, e^{i\lambda[g]}$ depend on the cutoff — as we now show. To begin, we consider the spinless rotation groups $G_\mathbf{x} = \{1, C_n, \dots, C_n^{n-1}\}$ with $C_n^n = +1$. (Shortly, we will study spinful electrons which have a rotation operator obeying $C_n^n = (-1)^{\hat{N}}$.) Extending the cutoff $R \to R'$ yields a new ground state which can be written

$$|GS,R'\rangle = \prod_{R<|\mathbf{r}|<R'} \mathcal{O}_\mathbf{r}\ |GS,R\rangle = \prod_{i=0}^{n-1} C_n^i \mathcal{O} C_n^{\dagger i}\ |GS,R\rangle \tag{21}$$

where, up to an irrelevant overall phase, $\mathcal{O} = \prod_{\mathbf{r}\in\mathcal{D}} \mathcal{O}_\mathbf{r}$ is a product of $\mathcal{O}_\mathbf{r}$ in the region $\mathcal{D}$ which obeys $\{R < |\mathbf{r}| < R'\} = \{\mathcal{D}, C_n\mathcal{D}, \dots, C_n^{-1}\mathcal{D}\}$ and $\mathcal{D}\cap C_n^i\mathcal{D} = \emptyset$. To be concrete, a possible choice is $\mathcal{D} = \{\mathbf{r}|R < r < R', 0 \leq \theta < 2\pi/n\}$. Eq. 21 holds because in the strict atomic limit, $C_n\mathcal{O}C_n^\dagger$ has zero overlap with $\mathcal{O}$. We call $N_\mathcal{O}$ the charge (number of

particles) of $\mathcal{O}$. The quantum numbers of the state with cutoff R' are

$$
\begin{aligned}
\hat{N}\left|GS,R'\right\rangle &= (N + nN_{\mathcal{O}})\left|GS,R'\right\rangle \ . \\
C_n\left|GS,R'\right\rangle &= \left(\prod_{i=1}^{n-1} C_n^i \mathcal{O} C_n^{\dagger i}\right) C_n^n \mathcal{O} C_n^{n\dagger}\ C_n\left|GS,R\right\rangle \\
&= e^{i\lambda[C_n]} \prod_{i=1}^{n-1} C_n^i \mathcal{O} C_n^{\dagger i}\ \mathcal{O}\ \left|GS,R\right\rangle \\
&= e^{i\lambda[C_n]}(-1)^{(n-1)N_{\mathcal{O}}^2}\ \left|GS,R'\right\rangle \\
&= e^{i\lambda[C_n]} \begin{cases} (-1)^{N_{\mathcal{O}}}, & n \text{ even} \\ 1, & n \text{ odd} \end{cases} \left|GS,R'\right\rangle
\end{aligned} \tag{22}
$$

where in the last line we used that fact that $\mathcal{O}\, C_n^i \mathcal{O} C_n^{\dagger i} = (-1)^{N_{\mathcal{O}}^2} C_n^i \mathcal{O} C_n^{\dagger i}\, \mathcal{O}$ since $C_n \mathcal{O} C_n^\dagger$ and $\mathcal{O}$ are supported on non-overlapping regions and each contain $N_{\mathcal{O}}$ fermionic operators. We see that both the $\hat{N}$ and C_n eigenvalue (if n is even) change under the expansion of the cutoff. The $(-1)^{N_{\mathcal{O}}}$ factor is simply from the fermionic parity of the operator. In a system of bosons, no $(-1)^{N_{\mathcal{O}}}$ factor appears, and the RSI groups (not considered here) are altered.

However, there are two quantities invariant under expansion of the cutoff for arbitrary $N_{\mathcal{O}}$:

$$
\begin{aligned}
e^{i\frac{2\pi}{n}\hat{N}}\left|GS,R\right\rangle &= e^{i\frac{2\pi}{n}\hat{N}}\left|GS,R'\right\rangle \\
e^{i\frac{\pi}{n}\hat{N}} C_n\left|GS,R\right\rangle &= e^{i\frac{\pi}{n}\hat{N}} C_n\left|GS,R'\right\rangle \quad (n \text{ even}) \\
C_n\left|GS,R\right\rangle &= C_n\left|GS,R'\right\rangle \qquad\qquad (n \text{ odd})
\end{aligned} \tag{23}
$$

corresponding to the total charge mod n and the many-body angular momentum of the $e^{i\frac{\pi}{n}\hat{N}} C_n$[4,5]. (Note that $e^{i\frac{\pi}{n}\hat{N}} C_n$ is effectively a "spinful" rotation since $(e^{i\frac{\pi}{n}\hat{N}} C_n)^n = (-1)^{\hat{N}}$ and C_n is a spin-less operator since $C_n^n = +1$.) Because the eigenvalues of $e^{i\frac{2\pi}{n}\hat{N}}, e^{i\frac{\pi}{n}\hat{N}} C_n$ (or C_n if n is odd) are independent of the cutoff, they are good quantum numbers for all choices of the cutoff. Then we can take $R \to \infty$ at constant density, which is the thermodynamic limit on infinite boundary conditions. Secondly, the eigenvalues of $e^{i\frac{2\pi}{n}\hat{N}}$ and C_n are quantized, so they cannot change if the ground state is perturbed away from the strict atomic limit without closing the gap. Thus small hoppings and off-site interactions can be added without changing the quantum numbers in Eq. 23. Hence we have shown that the eigenvalues of the operators in Eq. 23 are local, symmetry-protected quantum numbers invariant under the adiabatic expansion of the cutoff: they are many-body local RSIs. We remark that the many-body local RSIs are not typical invariants of $\left|GS\right\rangle$ in the thermodynamic limit. Usually, one computes the quantum numbers of a state with the Hilbert space fixed, whereas we demonstrated the existence of quantum numbers which are invariants upon enlarging the Hilbert space (while preserving density and symmetry). It would be desirable to have equivalent expressions for the many-body local RSIs which can be computed on a fixed Hilbert space without requiring OBCs. This is left for future work.

We now determine the group structure formed by the many-body local RSIs (Eq. 23). For even n, we have $(e^{i\frac{\pi}{n}\hat{N}} C_n)^{2n} = +1$, so the eigenvalues of $e^{i\frac{\pi}{n}\hat{N}} C_n$ are $\mathbb{Z}_{2n}$-classified. The eigenvalues of $e^{i\frac{2\pi}{n}\hat{N}}$ are $\mathbb{Z}_n$-classified, but they are not all independent from the eigenvalues of $e^{i\frac{\pi}{n}\hat{N}} C_n$ because $(e^{i\frac{\pi}{n}\hat{N}} C_n)^n = e^{i\pi\hat{N}} = (e^{i\frac{2\pi}{n}\hat{N}})^{n/2}$. Hence $e^{i\frac{2\pi}{n}\hat{N}}$ only provides $n/2$ more independent quantum numbers, and we obtain a $\mathbb{Z}_{2n} \times \mathbb{Z}_{n/2}$ classification for n even. To get a set of $\mathbb{Z}_{2n} \times \mathbb{Z}_{n/2}$ independent quantum numbers, we define the eigenvalues of the symmetry operators in Eq. 23 as

$$
\begin{aligned}
e^{i\frac{\pi}{n}\hat{N}} C_n\left|GS\right\rangle &= e^{i\frac{\pi}{n}\Delta_1}\left|GS\right\rangle, \qquad \Delta_1 \in \mathbb{Z}_{2n}, \qquad n \text{ even} \\
e^{-i\frac{2\pi}{n}\hat{N}}(e^{i\frac{\pi}{n}\hat{N}} C_n)^2\left|GS\right\rangle = C_n^2\left|GS\right\rangle &= e^{i\frac{2\pi}{n/2}\Delta_2}\left|GS\right\rangle, \qquad \Delta_2 \in \mathbb{Z}_{n/2}
\end{aligned} \tag{24}
$$

since $(C_n^2)^{n/2} = +1$. We will refer to Δ_1 and Δ_2 as the many-body local RSIs. Note that Eq. 22 can be used to show directly that Δ_2 is invariant under the cutoff, since $C_n^2\left|GS,R'\right\rangle = e^{i2\lambda[C_n]}(-1)^{2N_{\mathcal{O}}}\left|GS,R'\right\rangle = e^{i2\lambda[C_n]}\left|GS,R'\right\rangle$ with the $N_{\mathcal{O}}$-dependent phase canceling.

For n odd (only $n = 3$ is relevant for the crystalline point groups), the $\mathbb{Z}_n \times \mathbb{Z}_n$ group structure is obvious from Eq. 23 with $e^{i\frac{2\pi}{n}\hat{N}}$ and C_n providing independent $\mathbb{Z}_n$ quantum numbers. We define the eigenvalues as

$$
\begin{aligned}
e^{i\frac{2\pi}{n}\hat{N}}\left|GS\right\rangle &= e^{i\frac{2\pi}{n}\Delta_1}\left|GS\right\rangle, \qquad \Delta_1 \in \mathbb{Z}_n, \qquad n \text{ odd} \\
C_n\left|GS\right\rangle &= e^{i\frac{2\pi}{n}\Delta_2}\left|GS\right\rangle, \qquad \Delta_2 \in \mathbb{Z}_n
\end{aligned} \tag{25}
$$

We will refer to Δ_1 and Δ_2 as the many-body local RSIs.

Having obtained our key results, we offer a brief alternative perspective from the strict atomic limit. At any cutoff R, we can write the wavefunction of the groundstate of the OBC Hamiltonian Eq. 19 respecting the rotation group $G_{\mathbf{x}}$ as

$$|GS,R\rangle = \mathcal{O}_{\mathbf{x}} \prod_{i=1}^{n} \mathcal{O}_i \,|0\rangle\,, \qquad \mathcal{O}_i = C_n^i \mathcal{O} C_n^{\dagger i} \tag{26}$$

in analogy to Eq. 21, where $\mathcal{O} = \prod_{\mathbf{r}\in\mathcal{W}} \mathcal{O}_{\mathbf{r}}$ and $\mathcal{W}$ is the wedge $\{\mathbf{r}|0 < |\mathbf{r}-\mathbf{x}| < R, \arg(\mathbf{r}-\mathbf{x}) \in (0, 2\pi/n]\}$ (see Supplementary Figure 1b). Note that it is important $\mathcal{W}$ not contain $\mathbf{x}$, since $C_n\mathcal{W}$ must not overlap $\mathcal{W}$. For this reason, the operator $\mathcal{O}_{\mathbf{x}}$ is separated out in Eq. 26. Since the many-body local RSIs are constructed so that the operator $\prod_{i=1}^{n} \mathcal{O}_i$, which depends on the cutoff, does not contribute, we observe that the many-body local RSIs of $|GS,R\rangle$ are simply the many-body local RSIs of $\mathcal{O}_{\mathbf{x}}\,|0\rangle$. This statement relies on the strict atomic limit where $\mathcal{O}_{\mathbf{x}}$ can be defined since all operators are onsite, but Eq. 26 provides a useful intuitive picture.

As we have shown, the many-body local RSIs in Eqs. 24 and 25 remain invariant as the cutoff is taken to infinity. In this infinite limit, it is natural to consider the many-body local RSIs of each Wyckoff position in the unit cell. As an example in the space group $p2$, there are four Wyckoff positions $\mathbf{x} = (0,0), (1/2,0), (0,1/2), (1/2,1/2)$ with $G_{\mathbf{x}} = 2$ (in each unit cell). The point groups are generated by the symmetries $C_2, T_1C_2, T_2C_2, T_1T_2C_2$ respectively where T_i is the translation operator along the ith lattice vector. Choosing finite boundary conditions respecting the symmetries of a particular point group will necessarily break the symmetries of the other point groups, and thus our construction of the many-body local RSIs does not immediately imply they can be simultaneously defined.

While we do not provide a rigorous general argument, we claim that the many-body local RSIs are well-defined (for interacting atomic phases) in the infinite limit at each Wyckoff position in the unit cell. Consider the following heuristic argument. On infinite boundary conditions, the many-body local RSIs at each Wyckoff positions are well-defined in the strict atomic limit because the correlation length is zero and each Wyckoff position is decoupled. Hence their many-body local RSIs can be defined at each Wyckoff position. Adding weak hoppings and off-site interactions will make the correlation length nonzero, but the quantization of the RSI eigenvalues means they remain invariant throughout this process. Of course, the formal difficulty is that the eigenvalue of $\hat{N}$, the number of particles, is not well-defined in the infinite limit. We extend all the results of this section to general 2D point groups in Supplementary Note II D. We will give explicit symmetry operators whose quantum numbers (eigenvalues) are many-body local RSIs.

Lastly, we prove that the many-body local RSIs at two symmetry-related positions are identical. This statement holds on infinite boundary conditions (where the full wallpaper group is intact) according to the argument in the preceding paragraph, such that the many-body local RSIs on infinite boundary conditions are understood as the infinite-cutoff limit on OBCs. For instance, 1a sites in different unit cells of a crystal are related by translations and have the same many-body local RSIs. (In concrete terms, we can compute the many-body local RSI at the same Wyckoff position in two different unit cells by choosing two different OBCs which respect the symmetries of each site individually, though it is impossible to respect both at once. The resulting many-body RSIs will be the same since they are related by translations on infinite boundary conditions.) Alternatively, the two sites in the 2c $= \{(1/2,0),(0,1/2)\}$ position in wallpaper group $p4$ are related by C_4 and have the same many-body local RSIs protected by T_1C_2 and T_2C_2 respectively. As such, we can define $\Delta_{2c,1} = \Delta_{(1/2,0),1} = \Delta_{(0,1/2),1} \in \mathbb{Z}_4$. In other words, we can refer to the Wyckoff position 2c rather than either of its individual sites.

We now prove the general case. Consider a wallpaper group with two sites $\mathbf{x},\mathbf{x}'$ related by a symmetry: $\mathbf{x}' = g'\mathbf{x}, g' \in G$. The site symmetry groups $G_{\mathbf{x}}, G_{\mathbf{x}'}$ are isomorphic and related by conjugation: $G_{\mathbf{x}} \cong G'_{\mathbf{x}}$ with $G_{\mathbf{x}'} = \{h' = g'hg'^{\dagger} | h \in G_{\mathbf{x}}, g' \notin G_{\mathbf{x}}\}$. For instance, $\mathbf{x} = (1/2,0), \mathbf{x}' = (0,1/2)$ in $G = p4$ are the two sites of the 2c position with $g' = C_4$ discussed above, or in $G = p2$, we could consider $\mathbf{x} = (0,0), \mathbf{x}' = (1,0)$, which are 1a sites related by a translation $g' = T_1$. In the atomic limit where $\mathcal{O}_{\mathbf{r}}$ is the creation operator of the strictly local Hamiltonian $H_{\mathbf{r}}$, we now prove that $\mathcal{O}_{\mathbf{x}}$ and $\mathcal{O}'_{\mathbf{x}}$ have the same quantum numbers. To do so, define the transformation of $\mathcal{O}_{\mathbf{x}}$ by $h\mathcal{O}_{\mathbf{x}}h^{\dagger} = e^{iO[h]}\mathcal{O}_{\mathbf{x}}$. Then note that

$$h'\mathcal{O}_{\mathbf{x}'}h'^{\dagger} = g'hg'^{\dagger}\mathcal{O}_{\mathbf{x}'}g'h^{\dagger}g'^{\dagger} = e^{iO[g']}g'h\mathcal{O}_{\mathbf{x}}h^{\dagger}g'^{\dagger} = e^{iO[h]}e^{iO[g']}g'\mathcal{O}_{\mathbf{x}}g'^{\dagger} = e^{iO[h]}\mathcal{O}_{\mathbf{x}'} \tag{27}$$

using $g'^{\dagger}\mathcal{O}_{\mathbf{x}'}g' = e^{iO[g']}\mathcal{O}_{\mathbf{x}}$, with $e^{iO[g']}$ being an irrelevant phase factor. We saw earlier in Eq. 26 that the quantum numbers of $\mathcal{O}_{\mathbf{x}}$ completely determined the many-body local RSIs of the groundstate $|GS,R,\mathbf{x}\rangle = \mathcal{O}_{\mathbf{x}}\prod_{j=1}^{n}\mathcal{O}_j\,|0\rangle$. Because the quantum numbers of $\mathcal{O}_{\mathbf{x}}$ and $\mathcal{O}_{\mathbf{x}'}$ are the same, the RSIs of $|GS,R,\mathbf{x}\rangle$ and $|GS,R,\mathbf{x}'\rangle$ must be the same. This holds for all adiabatically connected states, completing the proof.

D. Extension to General Point Groups

In Supplementary Note II C, we gave a detailed derivation of the many-body local RSIs in the spinless point groups generated by C_n. To complete our RSI classification for other point groups with mirrors, time-reversal, and spin-orbit coupling (SOC), we develop the general theory of many-body RSIs. As before, we begin by working in zero correlation length atomic limit states, and then extend to adiabatically connected atomic states. Physically, expanding the cutoff and including more orbitals at a position $\mathbf{x}' \neq \mathbf{x}$ adds C_n-related copies of the orbitals to the Hilbert space. Like in the non-interacting case[3], these orbitals transforms as an irrep of the subgroup $G_{\mathbf{x}'} \in G_{\mathbf{x}}$. For instance with C_n and a mirror M_x, the addition of orbitals in the strict atomic limit described by $\mathcal{O} = \prod_{\mathbf{r}\in\mathcal{D}'} \mathcal{O}_{\mathbf{r}}$ where $\mathcal{D}' = \{R < |\mathbf{r}| < R', -\pi/n < \theta < \pi/n\}$ is mapped to a distinct region by C_n but is preserved under the mirror M taking $y \to -y$. Thus $\mathcal{O}$ transforms under an irrep of the reflection group M. In the rotation group cases we have considered so far with $G_{\mathbf{x}} = n$, the subgroup is trivial ($G_{\mathbf{x}'} = 1$), but in general there can be nontrivial subgroups of $G_{\mathbf{x}}$. There is a further requirement on $\mathcal{O}$: because we are in the strict atomic limit with a non-degenerate ground state, $\mathcal{O}$ must transform in a 1D irrep. Explicitly, for $G_{\mathbf{x}} = \{g_1 H, \dots, g_d H\}$ in a coset construction with $G_{\mathbf{x}'} = H$ and $d = |H|/|G|$, we have

$$h\mathcal{O}h^\dagger = e^{iO[h]}\mathcal{O}, \qquad \forall h \in H \subset G_{\mathbf{x}} \tag{28}$$

and the operators $g_i\mathcal{O}g_i^\dagger$ are supported on different orbitals for all $i = 1, \dots, d$ in the strict atomic limit. The representation $e^{iO[h]}, h \in H$ is a 1D irrep of H. For instance, if $\mathcal{O}$ is a product of orbital creation operators with definite parity under M, then $O[h = M]$ is simply the total many-body parity. Under rotations, $C_n\mathcal{O}C_n^\dagger$ is a new operator supported on different orbitals in the region $C_n\mathcal{D}$.

Now expanding the cutoff to introduce new operators at $g_1\mathbf{x}', \dots, g_d\mathbf{x}'$ (we always take $g_1 = \mathbb{1}$) yields the ground state

$$|GS, R'\rangle = \prod_{i=1}^{d} g_i\mathcal{O}g_i^\dagger \;\; |GS, R\rangle \;\; . \tag{29}$$

Importantly, we must find quantum numbers which are invariant under *all* possible expansions of the cutoff, which can include different orbitals transforming under any of the possible subgroups. To do so systematically, we recall some basic facts from the theory of induced representations. Because $\mathcal{O}$ transforms in a representation of H (in this case, a 1D irrep as required by non-degeneracy), it forms an induced representation of $G_{\mathbf{x}}$. To be explicit, this representation is the following. Define $\mathcal{O}_i = g_i\mathcal{O}g_i^\dagger, i = 1, \dots, d$ and the induced representation $R_{ij}[g]$ by

$$g\mathcal{O}_i g^\dagger = \sum_{j=1}^{d} R_{ij}[g]\mathcal{O}_j, \qquad \forall g \in G_{\mathbf{x}} \; . \tag{30}$$

For the spinless symmetries considered here where $C_n^n = +1$, $R[g]$ is a spinless representation of $G_{\mathbf{x}}$. Since $\mathcal{O}_i$ are distinct operators for each i in the strict atomic limit ($\mathcal{O}_i, \mathcal{O}_j$ are supported on non-overlapping sites for $i \neq j$), $R_{ij}[g]$ is a complex permutation matrix[3] in this basis: there is only one nonzero entry per row. As an example, consider $G_{\mathbf{x}} = 4mm$ which has $G_{\mathbf{x}'} = m$ as a nontrivial subgroup. Expand the cutoff to include the operator $\mathcal{O}$ supported in a finite region $\mathcal{D}'$ centered around the point $(x', 0) \neq \mathbf{q}$. Note that $M \in G_{\mathbf{x}}$ is also a symmetry of $G_{(x,0)}$, where M is a reflection acting as $M\hat{y} = -\hat{y}$, and denote the irrep of $\mathcal{O}$ by $M\mathcal{O}M^\dagger = \pm\mathcal{O}$. Since $(x, 0) \neq \mathbf{x}$, C_4 ensures the groundstate also contains the operators $C_4^i\mathcal{O}C_4^{i\dagger}, i = 1, 2, 3$ at position $(0, x), (-x, 0), (0, -x)$ respectively. From Eq. 30, the induced representation is

$$R[C_4] = \begin{pmatrix} 0 & & & 1 \\ 1 & 0 & & \\ & 1 & 0 & \\ & & 1 & 0 \end{pmatrix}, \quad R[M] = \pm \begin{pmatrix} 1 & & & \\ & 0 & & 1 \\ & & 1 & \\ & 1 & & 0 \end{pmatrix} \; . \tag{31}$$

We now need to compute the representation of g on the groundstate Eq. 29. To do so, we recall that $g\,|GS, R\rangle =$

$e^{i\lambda[g]}|GS,R\rangle$ and $\mathcal{O}_i\mathcal{O}_j = (-1)^{N_\mathcal{O}^2}\mathcal{O}_j\mathcal{O}_i = (-1)^{N_\mathcal{O}}\mathcal{O}_j\mathcal{O}_i$ in which case, upon enlarging the cutoff from R' to R gives

$$\begin{aligned} g\,|GS,R'\rangle &= g\prod_{i=1}^{d}\mathcal{O}_i\; g^\dagger g\,|GS,R\rangle \\ &= e^{i\lambda[g]}\prod_{i=1}^{d}\left(\sum_j R_{ij}[g]\mathcal{O}_j\right)\;|GS,R\rangle \\ &= e^{i\lambda[g]}\left(\det{}_{(-1)^{N_\mathcal{O}}}R[g]\right)\prod_{i=1}^{d}\mathcal{O}_i\;|GS,R\rangle \\ &= e^{i\lambda[g]}\left(\det{}_{(-1)^{N_\mathcal{O}}}R[g]\right)|GS,R'\rangle \end{aligned} \tag{32}$$

where $\det{}_\pm$ is the permanent for $+$ and determinant for $-$. Explicitly for a $d\times d$ matrix A,

$$\det{}_\pm A = \sum_\sigma (\pm1)^{\#_\sigma} A_{1,\sigma_1}\dots A_{d,\sigma_d} \tag{33}$$

where σ are the $d!$ permutations of the numbers $1,\dots,d$ and $\#_\sigma$ is the number of interchanges in the permutation. Because $D[g]$ is a complex permutation matrix with only one nonzero element per row, only one permutation σ' in the sum Eq. 33 over σ is nonzero. Thus

$$\det{}_\pm R[g] = (\pm1)^{\#_{\sigma'}} R[g]_{1,\sigma'_1}\dots R[g]_{d,\sigma'_d} \tag{34}$$

so that only the overall sign in Eq. 32 depends on the parity of $N_\mathcal{O}$ (through the signature $(-1)^{\#_{\sigma'}}$ of g).

We now give an example of this procedure in the point groups generated by C_4 and M. Consider expanding the cutoff to include the new operators $\mathcal{O}_i = C_4^i\mathcal{O}C_4^{\dagger i}$ where $\mathcal{O} = \prod_{\mathbf{r}\in\mathcal{D}'}\mathcal{O}_\mathbf{r}$. There are two possible nontrivial subgroups that $\mathcal{O}$ could transform under: $G_{\mathbf{x}'} = \{1,M\}$ if $\mathcal{D}'$ is centered around e.g. $(x,0)$ or $G_{\mathbf{x}'} = \{1,C_4M\}$ if $\mathcal{D}'$ is centered around e.g. (x,x). Here $x\neq0$ so $\mathcal{D}'$ cannot be invariant under C_4, and thus we do not consider the subgroups with C_4, as in the single-particle case[3]. For both cases, the number of particles increases as $N\to N+4N_\mathcal{O}$ where $N_\mathcal{O}$ is the number of particles in $\mathcal{O}$. Note that $N_\mathcal{O}$ can be even or odd, for instance if $\mathcal{D}$ encloses a single site with an even or odd number of orbitals. We start by considering the subgroup $\{1,M\}$. With Eq. 32, we find that

$$C_4\,|GS,R'\rangle = e^{i\lambda[C_4]}(-1)^{N_\mathcal{O}}\;|GS,R'\rangle \tag{35}$$

since the signature $(-1)^{\#_{\sigma'}} = -1$ of C_4 is negative. This obviously matches Eq. 22, recalling that $e^{i\lambda[C_4]}, e^{i\lambda[M]}$ are the eigenvalues of C_4 and M on $|GS,R\rangle$ respectively. We now need the representation of M. We find

$$M\,|GS,R'\rangle = e^{i\lambda[M]}(-1)^{N_\mathcal{O}}\;|GS,R'\rangle\,,\qquad H=\{1,M\} \tag{36}$$

since $R[M]$ in Eq. 31 also has odd signature. Now we consider the subgroup $\{1,C_4M\}$. The representation matrices are now

$$R[C_4] = \begin{pmatrix} 0 & & & 1\\ 1 & 0 & & \\ & 1 & 0 & \\ & & 1 & 0 \end{pmatrix},\quad R[M] = \pm\begin{pmatrix} & & & 1\\ & & 1 & \\ & 1 & & \\ 1 & & & \end{pmatrix} \tag{37}$$

and notably $R[M]$ now has even signature. So in this case

$$M\,|GS,R'\rangle = e^{i\lambda[M]}\;|GS,R'\rangle\,,\qquad H=\{1,C_4M\}\;. \tag{38}$$

Let us scrutinize Eqs. 35 and 38. On the original groundstate, the mirror eigenvalue is $M\,|GS,R\rangle = e^{i\lambda[M]}\,|GS,R\rangle$. Expanding the cutoff to obtain $|GS,R'\rangle$ can include operators transforming in one of two possible nontrivial subgroups. In both cases, the mirror eigenvalue is well-defined and is given by Eqs. 35 and 38, but crucially the mirror eigenvalue *differs* between the two cases by a factor of $(-1)^{N_\mathcal{O}}$. In order to obtain a many-body local RSI, it is essential to have a symmetry operator whose eigenvalues are invariant under any (symmetric) choice of cutoff, such as $e^{i\frac{\pi}{4}\hat{N}}C_n$. We see

from Eqs. 35 and 38 that, because $N_{\mathcal{O}}$ is unrestricted, we do not obtain another cutoff-independent quantum number from M.

In fact, this is the same as in the non-interacting RSIs. ?? showed that the non-interacting RSIs of $4mm$ can all be obtained by reducing the non-interacting RSIs of 4 in the presence of mirrors, but that mirrors did not introduce any new non-interacting RSIs. We check that for all even n, inducing from the $\{1, M\}$ and $\{1, C_nM\}$ subgroups gives factors of $e^{i\lambda[M]}$ and $e^{i\lambda[M]}(-1)^{N_{\mathcal{O}}}$ as in the $4mm$ example, and thus there are no RSIs protected by M. For n odd, we find that inducing the odd and even mirror irreps from $\{1, M\}$ brings factors of $\pm e^{i\lambda[M]}(-1)^{N_{\mathcal{O}}}$ which also prevents M from protecting a new RSI.

Lastly, we must consider how M restricts the possible eigenvalues of C_n, thereby reducing the C_n-protected many-body local RSIs. First we consider the case of n even. Since $MC_nM^\dagger = C_n^\dagger$ and $M|GS\rangle = e^{i\lambda[M]}|GS\rangle$ on a given groundstate, the only allowed C_n eigenvalues are real: ±1. Then Eq. 24 shows that $\Delta_2 = 0$, reducing the local RSI group from $\mathbb{Z}_{2n} \times \mathbb{Z}_{n/2} \to \mathbb{Z}_{2n}$. If n is odd, then $MC_nM^\dagger = C_n^\dagger$ enforces the C_n eigenvalue to be real, and thus $+1$ is the only possibility since -1 cannot be a C_n eigenvalue for n odd. Eq. 25 then shows $\Delta_2 = 0$, and the many-body local RSI group is reduced from $\mathbb{Z}_n \times \mathbb{Z}_n \to \mathbb{Z}_n$.

We now consider spinless time-reversal. This case is simpler since the only subgroup is $1'$ which with $\mathcal{T}^2 = +1$ has only the trivial irrep, so the induction is identical to the C_n case alone. Thus to derive the many-body local RSI, we only have to consider the constraints $\mathcal{T}$ imposes on the C_n eigenvalues. This is also very simple since, because $\mathcal{T}^2 = +1$, we can choose the overall phase of the groundstate such that $\mathcal{T}|GS, R\rangle = |GS, R\rangle$. Then $e^{i\lambda[C_n]}$ must be real because $[C_n, \mathcal{T}] = 0$. This is the same condition enforced by M, so following the same argument we find $\Delta_2 = 0$.

We remark that in all cases, the many-body local RSIs of all 2D point groups can obtained by reduction from their rotation subgroups.

Supplementary Table III summarizes the full classification obtained from adding mirrors and time-reversal to the rotation groups. We also must remark on the case of a point group $G_{\mathbf{x}} = m$ with only mirrors. This case is somewhat degenerate because M is a quasi-1D symmetry and its Wyckoff positions are extended lines, not points. When expanding the cutoff in 2D, it is possible to include an arbitrary number of each irrep on the mirror axis, and thus our prescription does not yield a many-body local RSI protected by M or by $e^{i\pi\hat{N}}$. In 1D there is no such issue because expanding the cutoff always induces orbitals from off the mirror plane.

PG	RSI Operators on $\|GS\rangle$	Classification
m		$\Delta_1 \in \mathbb{Z}_1$
2	$e^{i\frac{\pi}{2}\hat{N}}C_2 = e^{i\frac{\pi}{2}\Delta_1}$	$\Delta_1 \in \mathbb{Z}_4$
$2mm$	$e^{i\frac{\pi}{2}\hat{N}}C_2 = e^{i\frac{\pi}{2}\Delta_1}$	$\Delta_1 \in \mathbb{Z}_4$
3	$e^{i\frac{2\pi}{3}\hat{N}} = e^{i\frac{2\pi}{3}\Delta_1},\ C_3 = e^{i\frac{2\pi}{3}\Delta_2}$	$\Delta_1 \in \mathbb{Z}_3, \Delta_2 \in \mathbb{Z}_3$
$3m$	$e^{i\frac{2\pi}{3}\hat{N}} = e^{i\frac{2\pi}{3}\Delta_1}$	$\Delta_1 \in \mathbb{Z}_3$
4	$e^{i\frac{\pi}{4}\hat{N}}C_4 = e^{i\frac{\pi}{4}\Delta_1},\ C_4^2 = e^{i\pi\Delta_2}$	$\Delta_1 \in \mathbb{Z}_8, \Delta_2 \in \mathbb{Z}_2$
$4mm$	$e^{i\frac{\pi}{4}\hat{N}}C_4 = e^{i\frac{\pi}{4}\Delta_1}$	$\Delta_1 \in \mathbb{Z}_8$
6	$e^{i\frac{\pi}{6}\hat{N}}C_6 = e^{i\frac{\pi}{6}\Delta_1},\ C_6^2 = e^{i\frac{2\pi}{3}\Delta_2}$	$\Delta_1 \in \mathbb{Z}_{12}, \Delta_2 \in \mathbb{Z}_3$
$6mm$	$e^{i\frac{\pi}{6}\hat{N}}C_6 = e^{i\frac{\pi}{6}\Delta_1}$	$\Delta_1 \in \mathbb{Z}_{12}$

PG	RSI Operators on $\|GS\rangle$	Classification
m		$\Delta_1 \in \mathbb{Z}_1$
$21'$	$e^{i\frac{\pi}{2}\hat{N}}C_2 = e^{i\frac{\pi}{2}\Delta_1}$	$\Delta_1 \in \mathbb{Z}_4$
$2mm1'$	$e^{i\frac{\pi}{2}\hat{N}}C_2 = e^{i\frac{\pi}{2}\Delta_1}$	$\Delta_1 \in \mathbb{Z}_4$
$31'$	$e^{i\frac{2\pi}{3}\hat{N}} = e^{i\frac{2\pi}{3}\Delta_1}$	$\Delta_1 \in \mathbb{Z}_3$
$3m1'$	$e^{i\frac{2\pi}{3}\hat{N}} = e^{i\frac{2\pi}{3}\Delta_1}$	$\Delta_1 \in \mathbb{Z}_3$
$41'$	$e^{i\frac{\pi}{4}\hat{N}}C_4 = e^{i\frac{\pi}{4}\Delta_1}$	$\Delta_1 \in \mathbb{Z}_8$
$4mm1'$	$e^{i\frac{\pi}{4}\hat{N}}C_4 = e^{i\frac{\pi}{4}\Delta_1}$	$\Delta_1 \in \mathbb{Z}_8$
$61'$	$e^{i\frac{\pi}{6}\hat{N}}C_6 = e^{i\frac{\pi}{6}\Delta_1}$	$\Delta_1 \in \mathbb{Z}_{12}$
$6mm1'$	$e^{i\frac{\pi}{6}\hat{N}}C_6 = e^{i\frac{\pi}{6}\Delta_1}$	$\Delta_1 \in \mathbb{Z}_{12}$

TABLE III. Supplementary Table 3. Many-Body Local RSIs without SOC.

We now consider spin-orbit coupling (SOC) where $C_n^n = M^2 = \mathcal{T}^2 = (-1)^{\hat{N}}$. Without M and $\mathcal{T}$, the SOC case can be mapped immediately to the spinless (no SOC) case via $C_n = e^{i\frac{\pi}{n}\hat{N}}\tilde{C}_n$ where $\tilde{C}_n^n = +1$ is a spinless operator. Then Eq. 23 yields the same classification for Eqs. 24 and 25. This mapping would take $\tilde{C}_n^2 \to e^{-i\frac{2\pi}{n}\hat{N}}C_n^2$ for even n, but we find it more convenient to use the operator $e^{+i\frac{2\pi}{n}\hat{N}}C_n^2$ in order to obtain simple expressions matching the conventions of ??. This is clearly equivalent because the difference is a multiple of $e^{i\frac{2\pi}{n}\hat{N}}$ which is also a symmetry operator invariant under expansions of the cutoff, and $(e^{+i\frac{2\pi}{n}\hat{N}}C_n^2)^{n/2} = e^{i2\pi\hat{N}} = +1$. Explicitly, for n even we have (dropping the cutoff dependence R for brevity)

$$\begin{aligned} C_n|GS\rangle &= e^{i\frac{\pi}{n}\Delta_1}|GS\rangle\,, \qquad \Delta_1 \in \mathbb{Z}_{2n} \\ e^{i\frac{2\pi}{n}\hat{N}}C_n^2|GS\rangle &= e^{i\frac{4\pi}{n}\Delta_2}|GS\rangle\,, \qquad \Delta_2 \in \mathbb{Z}_{n/2} \end{aligned} \tag{39}$$

which is to be compared with Eq. 24, and for n odd we have

$$\begin{aligned} e^{i\frac{2\pi}{n}\hat{N}}\,|GS\rangle &= e^{i\frac{2\pi}{n}\Delta_1}\,|GS\rangle\,, \qquad \Delta_1 \in \mathbb{Z}_n \\ e^{-i\frac{\pi}{n}\hat{N}}C_n\,|GS\rangle &= e^{i\frac{2\pi}{n}\Delta_2}\,|GS\rangle\,, \qquad \Delta_2 \in \mathbb{Z}_n \end{aligned} \tag{40}$$

to be compared with Eq. 25. The addition of mirrors and time-reversal with SOC differs from the case without SOC, as is also true in the single-particle case[3]. We first consider adding only mirror symmetry M. Again we must consider the mirror subgroups as discussed in Eq. 36. Following identical steps, we check that M does not protect an additional RSI (exactly like in the spinless groups) and merely reduces the C_n-protected RSIs. We derive these reductions now.

Because $MC_nM^\dagger = C_n^\dagger$, mirrors enforce $C_n\,|GS,R\rangle = \pm\,|GS,R\rangle$ which gives a $\mathbb{Z}_2$ quantum number. Then for even n, we find $(-1)^{\hat{N}} = C_n^n = (\pm 1)^n = +1$ on $|GS,R\rangle$, so the number of particles is even if the groundstate is non-degenerate (as we have assumed throughout). Thus $e^{i\frac{2\pi}{n}\hat{N}}$, which computes the number of particles mod n, only provides a $\mathbb{Z}_{n/2}$ quantum number. The many-body local RSIs are the eigenvalues of

$$\begin{aligned} C_n\,|GS\rangle &= e^{i\pi\Delta_1}\,|GS\rangle\,, \qquad \Delta_1 \in \mathbb{Z}_2 \\ e^{i\frac{2\pi}{n}\hat{N}}C_n^2\,|GS\rangle = e^{i\frac{2\pi}{n}\hat{N}}\,|GS\rangle &= e^{i\frac{2\pi}{n/2}\Delta_2}\,|GS\rangle\,, \qquad \Delta_2 \in \mathbb{Z}_{n/2} \end{aligned} \tag{41}$$

and the many-body local RSI classification is $\mathbb{Z}_2 \times \mathbb{Z}_{n/2}$.

We now consider the case where n is odd. Then because the C_n eigenvalue must be real (other $\mathcal{T}$ would enforce a double-degeneracy in contraction to our assumption of a non-degenerate groundstate), it follows that $(e^{-i\frac{\pi}{n}\hat{N}}C_n)^2 = e^{-i\frac{2\pi}{n}\hat{N}}C_n^2 = e^{-i\frac{2\pi}{n}\hat{N}}$ which reduces the independent RSIs, so we could pick the eigenvalues of $e^{-i\frac{\pi}{n}\hat{N}}C_n$ as the RSIs since $e^{i\frac{2\pi}{n}\hat{N}}$ is determined from them. However, it will be simpler to pick a convention when the eigenvalues of $e^{i\frac{2\pi}{n}\hat{N}}$ are the many-body local RSIs. This is an equivalent choice because

$$e^{-i\frac{\pi}{n}\hat{N}}C_n = \left((e^{-i\frac{\pi}{n}\hat{N}}C_n)^2\right)^{(n+1)/2} = \left(e^{-i\frac{2\pi}{n}\hat{N}}\right)^{(n+1)/2} \tag{42}$$

using $(e^{-i\frac{\pi}{n}\hat{N}}C_n)^n = +1$ and that fact that n is odd so $(n+1)/2$ is an integer. Thus $e^{-i\frac{\pi}{n}\hat{N}}C_n$ is entirely determined by $e^{i\frac{2\pi}{n}\hat{N}}$ and we can choose the many-body local RSIs to be

$$e^{i\frac{2\pi}{n}\hat{N}}\,|GS\rangle = e^{i\frac{2\pi}{n}\Delta_1}\,|GS\rangle\,, \qquad \Delta_1 \in \mathbb{Z}_n\ . \tag{43}$$

We now consider the addition of spinful $\mathcal{T}$ (without mirrors) obeying $\mathcal{T}^2 = (-1)^{\hat{N}}$. Because $\mathcal{O}$ transforms in a 1D irrep since we require the groundstate to be non-degenerate, $N_{\mathcal{O}} = 2p_{\mathcal{O}}$ must be even where $p_{\mathcal{O}}$ is the number of Kramers pairs. Hence $\mathcal{T}^2 = +1$ on the (non-degenerate) groundstate. If $N_{\mathcal{O}}$ were odd, Kramer's theorem would forbid a non-degenerate state. We now use Eq. 32 to determine the C_n eigenvalue recalling $N_{\mathcal{O}}$ is even:

$$C_n\,|GS,R'\rangle = e^{i\lambda[C_n]}\det{}_+R[C_n]\,|GS,R'\rangle = e^{i\lambda[C_n]}\,|GS,R'\rangle \tag{44}$$

where $g\mathcal{O}_ig^\dagger = \sum_j R_{ij}[g]\mathcal{O}_j$ and $R[g]$ is an *spinless* representation since $\mathcal{O}$ has an even number of particles and hence $C_n^n\mathcal{O}_iC_n^{n\dagger} = +\mathcal{O}_i$. Then because $R[C_n]$ is a permutation matrix, $\det_+ R[C_n] = +1$. Thus the C_n eigenvalue of the groundstate is a good many-body local RSI, but $\mathcal{T}$ restricts this eigenvalue to be real. If n is even, $C_n = \pm 1$ is allowed since $(-1)^{\hat{N}}\,|GS,R\rangle = C_n^n\,|GS,R\rangle = (\pm 1)^n\,|GS\rangle = +\,|GS\rangle$. If n is odd, then necessarily $C_n\,|GS\rangle = +\,|GS\rangle$ since the number of particles in $|GS\rangle$ is even due to $\mathcal{T}$ and the requirement of single-degeneracy. We define the many-body local RSIs as

$$\begin{aligned} &C_n\,|GS,R\rangle = e^{i\pi\Delta_1}\,|GS,R\rangle\,, \qquad e^{i\frac{2\pi}{n}\frac{\hat{N}}{2}}\,|GS\rangle = e^{i\frac{2\pi}{n}\Delta_2}\,|GS\rangle\,, \qquad \Delta_1,\Delta_2 \in \mathbb{Z}_2,\mathbb{Z}_n \qquad (n \text{ even}) \\ &e^{i\frac{2\pi}{n}\frac{\hat{N}}{2}}\,|GS\rangle = e^{i\frac{2\pi}{n}\Delta_1}\,|GS\rangle\,, \qquad \Delta_1 \in \mathbb{Z}_n \qquad (n \text{ odd}) \end{aligned} \tag{45}$$

noting that $\hat{N}/2$ counts the number of Kramer's pairs which is defined mod n. We check that adding mirrors gives the identical classification. The results are summarized in Supplementary Table IV.

PG	RSI Operators on $\lvert GS\rangle$	Classification
m		$\Delta_1 \in \mathbb{Z}_1$
2	$C_2 = e^{i\frac{\pi}{2}\Delta_1}$	$\Delta_1 \in \mathbb{Z}_4$
$2mm$	$C_2 = e^{i\pi\Delta_1}$	$\Delta_1 \in \mathbb{Z}_2$
3	$e^{i\frac{2\pi}{3}\hat{N}} = e^{i\frac{2\pi}{3}\Delta_1}, e^{-\frac{i\pi\hat{N}}{3}}C_3 = e^{i\frac{2\pi}{3}\Delta_2}$	$\Delta_1 \in \mathbb{Z}_3, \Delta_2 \in \mathbb{Z}_3$
$3m$	$e^{i\frac{2\pi}{3}\hat{N}} = e^{i\frac{2\pi}{3}\Delta_1}$	$\Delta_1 \in \mathbb{Z}_3$
4	$C_4 = e^{i\frac{\pi}{4}\Delta_1},\ e^{i\frac{2\pi}{4}\hat{N}}C_4^2 = e^{i\pi\Delta_2}$	$\Delta_1 \in \mathbb{Z}_8, \Delta_2 \in \mathbb{Z}_2$
$4mm$	$C_4 = e^{i\pi\Delta_1}, e^{i\frac{2\pi}{4}\hat{N}} = e^{i\pi\Delta_2}$	$\Delta_1 \in \mathbb{Z}_2, \Delta_2 \in \mathbb{Z}_2$
6	$C_6 = e^{i\frac{\pi}{6}\Delta_1},\ e^{i\frac{2\pi}{6}\hat{N}}C_6^2 = e^{i\frac{2\pi}{3}\Delta_2}$	$\Delta_1 \in \mathbb{Z}_{12}, \Delta_2 \in \mathbb{Z}_3$
$6mm$	$C_6 = e^{i\pi\Delta_1}, e^{i\frac{2\pi}{6}\hat{N}} = e^{i\frac{2\pi}{3}\Delta_2}$	$\Delta_1 \in \mathbb{Z}_2, \Delta_2 \in \mathbb{Z}_3$

PG	RSI Operators on $\lvert GS\rangle$	Classification
$m1'$		$\Delta_1 \in \mathbb{Z}_1$
$21'$	$C_2 = e^{i\pi\Delta_1}, e^{i\pi\frac{\hat{N}}{2}} = e^{i\pi\Delta_2}$	$\Delta_1 \in \mathbb{Z}_2, \Delta_2 \in \mathbb{Z}_2$
$2mm1'$	$C_2 = e^{i\pi\Delta_1}, e^{i\pi\frac{\hat{N}}{2}} = e^{i\pi\Delta_2}$	$\Delta_1 \in \mathbb{Z}_2, \Delta_2 \in \mathbb{Z}_2$
$31'$	$e^{i\frac{2\pi}{3}\frac{\hat{N}}{2}} = e^{i\frac{2\pi}{3}\Delta_1}$	$\Delta_1 \in \mathbb{Z}_3$
$3m1'$	$e^{i\frac{2\pi}{3}\frac{\hat{N}}{2}} = e^{i\frac{2\pi}{3}\Delta_1}$	$\Delta_1 \in \mathbb{Z}_3$
$41'$	$C_4 = e^{i\pi\Delta_1}, e^{i\frac{\pi}{2}\frac{\hat{N}}{2}} = e^{i\frac{\pi}{2}\Delta_2}$	$\Delta_1 \in \mathbb{Z}_2, \Delta_2 \in \mathbb{Z}_4$
$4mm1'$	$C_4 = e^{i\pi\Delta_1}, e^{i\frac{\pi}{2}\frac{\hat{N}}{2}} = e^{i\frac{\pi}{2}\Delta_2}$	$\Delta_1 \in \mathbb{Z}_2, \Delta_2 \in \mathbb{Z}_4$
$61'$	$C_6 = e^{i\pi\Delta_1}, e^{i\frac{2\pi}{6}\frac{\hat{N}}{2}} = e^{i\frac{2\pi}{6}\Delta_2}$	$\Delta_1 \in \mathbb{Z}_2, \Delta_2 \in \mathbb{Z}_6$
$6mm1'$	$C_6 = e^{i\pi\Delta_1}, e^{i\frac{2\pi}{6}\frac{\hat{N}}{2}} = e^{i\frac{2\pi}{6}\Delta_2}$	$\Delta_1 \in \mathbb{Z}_2, \Delta_2 \in \mathbb{Z}_6$

TABLE IV. Supplementary Table 4. Many-Body Local RSIs with SOC.

E. Weakly Interacting Limit

In this section, we evaluate the many-body local RSIs when acting on product states, which have been classified in terms of single-particle RSIs[3]. Since a product state is specified by the multiplicities of each of the irreps of the point group, we will obtain expressions for the many-body local RSIs in terms of the single-particle irrep multiplicities. We then compare these expressions to the expressions for the non-interacting RSIs (which are also defined on product states) and determine the reduction of the non-interacting RSI classification due to interactions. These expressions will also give us a way to evaluate the many-body local RSIs in terms of the non-interacting RSIs in the weak coupling limit.

We first study $G_{\mathbf{x}} = 2$ generated by C_2. In a product state with $m(A)$ even irreps and $m(B)$ odd irreps (see the irrep tables in Supplementary Table I), we find

$$e^{i\frac{\pi}{2}\Delta_1}\lvert GS\rangle = e^{i\frac{\pi}{2}\hat{N}}C_2\lvert GS\rangle = e^{i\frac{\pi}{2}(m(A)+m(B))}e^{i\pi m(B)}\lvert GS\rangle = e^{i\frac{\pi}{2}(m(A)-m(B))}\lvert GS\rangle \tag{46}$$

so we see that the many-body local RSI is given by $\Delta_1 = m(A) - m(B) \mod 4$.

Next we study $G_{\mathbf{x}} = 3$ generated by C_3. Following Supplementary Table I, we find that

$$\begin{aligned} e^{i\frac{2\pi}{3}\hat{N}}\lvert GS\rangle &= e^{i\frac{2\pi}{3}(m(A)+m(^1E)+m(^2E))}\lvert GS\rangle, \\ C_n\lvert GS\rangle &= e^{i\frac{2\pi}{3}(m(^2E)-m(^1E))}\lvert GS\rangle \end{aligned} \tag{47}$$

so $\Delta_1 = m(A) + m(^1E) + m(^2E) \mod 3$ and $\Delta_2 = m(^2E) - m(^1E) \mod 3$ are good many-body local RSIs giving a $\mathbb{Z}_3 \times \mathbb{Z}_3$ classification. Formulae for the other spinless groups follow identically. In particular, we find that all values of the many-body local RSIs in all the spinless groups can be obtained in the non-interacting limit from product states.

However, with SOC and M or $\mathcal{T}$, we find that not all possible many-body RSIs can be obtained in the single-particle limit. Recall that M or $\mathcal{T}$ impose $C_n\lvert GS\rangle = \pm\lvert GS\rangle$. Only $+$ sign is possible for single-particle states since M and $\mathcal{T}$ pair the C_n eigenvalues with their complex conjugates into double irreps. For example, consider $21'$ with SOC whose single irrep is $^1\overline{\mathrm{E}}^2\overline{\mathrm{E}}$ with the representation $D[C_2] = i\sigma_z$. From Supplementary Table IV, the many-body local RSIs of $21'$ are $C_2\lvert GS\rangle = e^{i\pi\Delta_1}\lvert GS\rangle$ and $e^{i\pi\hat{N}/2}\lvert GS\rangle = e^{i\pi\Delta_2}\lvert GS\rangle$. All possible non-interacting product states are built from the operators in the form $c^\dagger_{+i}c^\dagger_{-i}$ which carry the $^1\overline{\mathrm{E}}^2\overline{\mathrm{E}}$ irrep. But since $C_2c^\dagger_{+i}c^\dagger_{-i}C_2^\dagger = (+i)(-i)c^\dagger_{+i}c^\dagger_{-i} = +c^\dagger_{+i}c^\dagger_{-i}$, we find that $\Delta_1 = 0 \mod 2$ on all such states. Since $[\hat{N}, c^\dagger_{+i}c^\dagger_{-i}] = 2c^\dagger_{+i}c^\dagger_{-i}$, we see that $\Delta_2 = m(^1\overline{\mathrm{E}}^2\overline{\mathrm{E}}) \mod 2$ which simply counts the number of Kramers pairs. However, it is simple to write down a state with $\Delta_1 = 1 \mod 2$. For instance, we take $\lvert GS\rangle = \frac{1}{\sqrt{2}}(c^\dagger_{+i,1}c^\dagger_{+i,2} + c^\dagger_{-i,1}c^\dagger_{-i,2})\lvert 0\rangle$ whose terms individually would be degenerate by Kramers theorem. We compute

$$\begin{aligned} C_2\lvert GS\rangle &= \frac{1}{\sqrt{2}}((+i)^2c^\dagger_{+i,1}c^\dagger_{+i,2} + (-i)^2c^\dagger_{-i,1}c^\dagger_{-i,2})\lvert 0\rangle = -\lvert GS\rangle \\ \mathcal{T}\lvert GS\rangle &= \frac{1}{\sqrt{2}}(c^\dagger_{-i,1}c^\dagger_{-i,2} + (-1)^2c^\dagger_{+i,1}c^\dagger_{+i,2})\lvert 0\rangle = \lvert GS\rangle \end{aligned} \tag{48}$$

which shows that $|GS\rangle$ has $\Delta_1 = 1 \mod 2$ and is allowed to be non-degenerate since $\mathcal{T}$ squares to $+1$ on $|GS\rangle$. Because we showed above that $\Delta_1 = 0$ in all product states, the many-body local RSI $\Delta_1 = 1 \mod 2$ proves that $|GS\rangle$ cannot be adiabatically connected to any product state. The fact that our classification includes states with $\Delta_1 \neq 0$, which is impossible in any non-interacting atomic limit state, underscores our non-perturbative construction of the many-body local RSIs.

TABLE V: Supplementary Table 5. Many-Body Local RSIs of Product States in terms of Irrep Multiplicities

RSIs	No SOC $(C_n^n = M_i^2 = +1)$		SOC $(C_n^n = M_i^2 = (-1)^{\tilde{N}})$	
	No TRS	TRS $(\mathcal{T}^2 = +1)$	No TRS	TRS $(\mathcal{T}^2 = (-1)^{\tilde{N}})$
m				
2	$\Delta_1 = m(A) - m(B) \mod 4$	$\Delta_1 = m(A) - m(B) \mod 4$	$\Delta_1 = m(^1\overline{\mathrm{E}}) - m(^2\overline{\mathrm{E}}) \mod 4$	$\Delta_1 = 0 \mod 2$, $\Delta_2 = m(^1\overline{\mathrm{E}}{}^2\overline{\mathrm{E}}) \mod 2$
$2mm$	$\Delta_1 = m(A_1)+m(A_2)-m(B_1)-m(B_2) \mod 4$	$\Delta_1 = m(A_1)+m(A_2)-m(B_1)-m(B_2) \mod 4$	$\Delta_1 = 0 \mod 2$	$\Delta_1 = 0 \mod 2$, $\Delta_2 = m(\overline{\mathrm{E}}) \mod 2$
4	$\Delta_1 = m(A) - 3m(B) - m(^1\mathrm{E}) + 3m(^2\mathrm{E}) \mod 8$<br>$\Delta_2 = m(^1\mathrm{E}) - m(^2\mathrm{E}) \mod 2$	$\Delta_1 = m(A) - 3m(B) + 2m(^1\mathrm{E}{}^2\mathrm{E}) \mod 8$	$\Delta_1 = m(^1\overline{\mathrm{E}}_1) - m(^2\overline{\mathrm{E}}_1) - m(^2\overline{\mathrm{E}}_2) + m(^1\overline{\mathrm{E}}_2) \mod 8$<br>$\Delta_2 = m(^1\overline{\mathrm{E}}_1) - m(^1\overline{\mathrm{E}}_2) \mod 2$	$\Delta_1 = 0 \mod 2$<br>$\Delta_2 = m(^1\overline{\mathrm{E}}_1{}^2\overline{\mathrm{E}}_1) + m(^1\overline{\mathrm{E}}_2{}^2\overline{\mathrm{E}}_2) \mod 4$
$4mm$	$\Delta_1 = m(A_1)+m(A_2)-3m(B_1)-3m(B_2) + 2m(E) \mod 8$	$\Delta_1 = m(A_1)+m(A_2)-3m(B_1)-3m(B_2) + 2m(E) \mod 8$	$\Delta_1 = 0 \mod 2$, $\Delta_2 = m(\overline{\mathrm{E}}_1) + m(\overline{\mathrm{E}}_2) \mod 2$	$\Delta_1 = 0 \mod 2$, $\Delta_2 = m(E_1) + m(E_2) \mod 4$
3	$\Delta_1 = m(A) + m(^1E) + m(^2E) \mod 3$<br>$\Delta_2 = m(^2E) - m(^1E) \mod 3$	$\Delta_1 = m(A) + 2m(^1E\,^2E) \mod 3$	$\Delta_1 = m(\overline{\mathrm{E}}) + m(^1\overline{\mathrm{E}}) + m(^2\overline{\mathrm{E}}) \mod 3$<br>$\Delta_2 = m(\overline{\mathrm{E}}) - m(^1\overline{\mathrm{E}}) \mod 3$	$\Delta_1 = m(\overline{\mathrm{E}}\overline{\mathrm{E}}) + m(^1\overline{\mathrm{E}}{}^2\overline{\mathrm{E}}) \mod 3$
$3m$	$\Delta_1 = m(A_1) + m(A_2) + 2m(E) \mod 3$	$\Delta_1 = m(A_1) + m(A_2) + 2m(E) \mod 3$	$\Delta_1 = -m(\overline{\mathrm{E}}_1) + m(^1\overline{\mathrm{E}}) + m(^2\overline{\mathrm{E}}) \mod 3$	$\Delta_1 = m(\overline{\mathrm{E}}_1) + m(^1\overline{\mathrm{E}}{}^2\overline{\mathrm{E}}) \mod 3$
6	$\Delta_1 = m(A) - 5m(B) - m(^1\mathrm{E}_2) + 3m(^2\mathrm{E}_2) + 5m(^1\mathrm{E}_1) - 3m(^2\mathrm{E}_1) \mod 12$<br>$\Delta_2 = 2m(^1\mathrm{E}_2) + m(^2\mathrm{E}_2) + 2m(^1\mathrm{E}_1) + m(^2\mathrm{E}_1) \mod 3$	$\Delta_1 = m(A) - 5m(B) + 2m(^1\mathrm{E}_2{}^2\mathrm{E}_2) + 2m(^1\mathrm{E}_1{}^2\mathrm{E}_1) \mod 12$	$\Delta_1 = m(^2\overline{\mathrm{E}}_3) - m(^1\overline{\mathrm{E}}_3) - 3m(^2\overline{\mathrm{E}}_1) + 3m(^1\overline{\mathrm{E}}_1) - 5m(^2\overline{\mathrm{E}}_2) + 5m(^1\overline{\mathrm{E}}_2) \mod 12$<br>$\Delta_2 = m(^2\overline{\mathrm{E}}_3) - m(^2\overline{\mathrm{E}}_1) - m(^1\overline{\mathrm{E}}_1) + m(^2\overline{\mathrm{E}}_2) \mod 3$	$\Delta_1 = 0 \mod 2$<br>$\Delta_2 = m(^1\overline{\mathrm{E}}_1{}^2\overline{\mathrm{E}}_1) + m(^1\overline{\mathrm{E}}_2{}^2\overline{\mathrm{E}}_2) + m(^1\overline{\mathrm{E}}_3{}^2\overline{\mathrm{E}}_3) \mod 6$
$6mm$	$\Delta_1 = m(A_1) + m(A_2) - 5m(B_1) - 5m(B_2) + 2m(\mathrm{E}_1) + 2m(\mathrm{E}_2) \mod 12$	$\Delta_1 = m(A_1) + m(A_2) - 5m(B_1) - 5m(B_2) + 2m(\mathrm{E}_1) + 2m(\mathrm{E}_2) \mod 12$	$\Delta_1 = 0 \mod 2$, $\Delta_2 = m(\overline{\mathrm{E}}_1) + m(\overline{\mathrm{E}}_2) + m(\overline{\mathrm{E}}_3) \mod 3$	$\Delta_1 = 0 \mod 2$, $\Delta_2 = m(\overline{\mathrm{E}}_1) + m(\overline{\mathrm{E}}_2) + m(\overline{\mathrm{E}}_3) \mod 6$

Having derived expressions for the many-body local RSIs (see Supplementary Table V) in terms of the irrep multiplicities in the weak coupling limit (some of which are identically zero without interactions), we now show that they can be expressed in terms of the single-particle RSIs first derived in ??. This is a good consistency check of our definition of the many-body RSIs which is a priori much different than the group theoretical construction of ??. It will also be instructive to see how the single-particle RSIs collapse to many-body RSIs as weak interactions are turned on. In Supplementary Table VI, we collect these reductions explicitly. If a gap is not closed, then the many-body local RSIs computed from the single-particle RSIs describe the interacting groundstate of the system.

We now give an example of one of the calculations in Supplementary Table VI. Let us study PG $41'$ without SOC. This group has 3 co-irreps which are presented by

$$
\begin{aligned}
A: &\quad D_A[C_4] = 1, D_A[\mathcal{T}] = K \\
B: &\quad D_B[C_4] = -1, D_B[\mathcal{T}] = K \\
{}^1E\,{}^2E: &\quad D_{^1E^2E}[C_4] = \begin{pmatrix} i & 0 \\ 0 & -i \end{pmatrix}, D_{^1E^2E}[\mathcal{T}] = \begin{pmatrix} 0 & 1 \\ 1 & 0 \end{pmatrix} K
\end{aligned}
\tag{49}
$$

where K is complex conjugation. The non-interacting RSIs are invariant under the induction/reduction of $E \uparrow 4 = A \oplus B \oplus {}^1E^2E^3$. We find a $\mathbb{Z}^2$ classification

$$
\delta_1 = -m(A) + m(^1E^2E), \quad \delta_2 = -m(A) + m(B) \ . \tag{50}
$$

From Supplementary Table V, we see that the many-body local RSI is

$$\Delta_1 = m(A) - 3m(B) + 2m({}^1\mathrm{E}^2\mathrm{E}) \mod 8 \tag{51}$$

when evaluated on a product state. Now observe that

$$2\delta_1 - 3\delta_2 \mod 8 = -2m(A) + 2m({}^1E^2E) + 3m(A) - 3m(B) \mod 8 = \Delta_1 \ . \tag{52}$$

Identical calculations hold in the other groups.

TABLE VI: Supplementary Table 6. Many-Body Local RSI Reduction to Single-Particle RSIs in Product States

RSIs	No SOC ($C_n^n = M_i^2 = \mathcal{T}^2 = +1$)		SOC ($C_n^n = M_i^2 = \mathcal{T}^2 = (-1)^{\hat{N}}$)	
	No TRS	TRS	No TRS	TRS
m				
2	$\Delta_1 = -\delta_1 \mod 4$	$\Delta_1 = -\delta_1 \mod 4$	$\Delta_1 = -\delta_1 \mod 4$	$\Delta_1 = 0,\ \Delta_2 = \delta_1$
$2mm$	$\Delta_1 = -\delta_1 \mod 4$	$\Delta_1 = -\delta_1 \mod 4$	$\Delta_1 = 0$	$\Delta_1 = 0,\ \Delta_2 = \delta_1 \mod 2$
4	$\Delta_1 = 3\delta_1 - 3\delta_2 - \delta_3 \mod 8$<br>$\Delta_2 = \delta_1 - \delta_3 \mod 2$	$\Delta_1 = 2\delta_1 - 3\delta_2 \mod 8$	$\Delta_1 = 3\delta_1 - 3\delta_2 - \delta_3 \mod 8$<br>$\Delta_2 = \delta_1 - \delta_3 \mod 2$	$\Delta_1 = 0$<br>$\Delta_2 = \delta_1 - 2\delta_2 \mod 4$
$4mm$	$\Delta_1 = 2\delta_1 - 3\delta_2 \mod 8$	$\Delta_1 = 2\delta_1 - 3\delta_2 \mod 8$	$\Delta_1 = 0,\ \Delta_2 = \delta_1 \mod 2$	$\Delta_1 = 0,\ \Delta_2 = \delta_1 - 2\delta_2 \mod 4$
3	$\Delta_1 = \delta_1 + \delta_2 \mod 3$<br>$\Delta_2 = \delta_2 - \delta_1 \mod 3$	$\Delta_1 = -\delta_1 \mod 3$	$\Delta_1 = \delta_1 + \delta_2 \mod 3$<br>$\Delta_2 = \delta_2 - \delta_1 \mod 3$	$\Delta_1 = -\delta_1 \mod 3$
$3m$	$\Delta_1 = -\delta_1 \mod 3$	$\Delta_1 = -\delta_1 \mod 3$	$\Delta_1 = \delta_1 \mod 3$	$\Delta_1 = -\delta_1 \mod 3$
6	$\Delta_1 = -\delta_1 - 5\delta_3 + 3\delta_5 + 5\delta_4 - 3\delta_2 \mod 12$<br>$\Delta_2 = 2\delta_1 + \delta_5 + 2\delta_4 + \delta_2 \mod 3$	$\Delta_1 = 2\delta_1 + 2\delta_2 - 5\delta_3 \mod 12$	$\Delta_1 = -\delta_1 - 5\delta_3 + 3\delta_5 + 5\delta_4 - 3\delta_2 \mod 12$<br>$\Delta_2 = 2\delta_1 + \delta_5 + 2\delta_4 + \delta_2 \mod 3$	$\Delta_1 = 0$<br>$\Delta_2 = \delta_1 + \delta_2 - 3\delta_3 \mod 6$
$6mm$	$\Delta_1 = 2\delta_1 + 2\delta_2 - 5\delta_3 \mod 12$	$\Delta_1 = 2\delta_1 + 2\delta_2 - 5\delta_3 \mod 12$	$\Delta_1 = 0, \Delta_2 = \delta_1 + \delta_2 \mod 3$	$\Delta_1 = 0,\ \Delta_2 = \delta_1 + \delta_2 - 3\delta_3 \mod 6$

We see that in all cases, the many-body local RSIs can be written in terms of single-particle RSIs. As we will show in Supplementary Note III A, certain single-particle fragile topology can be trivialized by adding interactions due to the reduction of single-particle RSIs to many-body local RSIs because of the mod factors in Supplementary Table VI. Convenient expressions for the single-particle RSIs in terms of the momentum space irreps may be found in the appendices of ?? and thus Supplementary Table VI gives a direct map from single-particle topology to many-body fragile topology in the weak coupling limit.

III. SUPPLEMENTARY NOTE 3

A. Fragile Topology and Constraints on Particle Number

In this Supplementary Note, we define and study many-body fragile topology using many-body local RSIs. In Supplementary Note III B, we derive constraints between the many-body RSIs and the minimum number of particles necessary to obtain the many-body local RSIs in a many-body atomic state. In many-body fragile topological phases, the many-body local RSIs can be uniquely defined at each Wyckoff position through the addition of many-body atomic states (Supplementary Note III C). Such phases are indicated when an inequality comparing RSIs to the $U(1)$ particle number in any many-body atomic state is violated, indicating an obstruction to adiabatic deformation into a many-body atomic state. We enumerate inequality criteria in all 2D wallpaper groups with and without SOC and time-reversal in Supplementary Note III D.

B. Orbital Number Constraints

In this section, we produce constraints between the many-body local RSIs and the total particle number per unit cell in many-body atomic states. In particular, we find that nonzero many-body local RSIs lower bound the particle

number. The inequalities derived here are crucial for determining many-body fragile topological indices. We consider a fixed Wyckoff position $\mathbf{x}$ and let N denote the total charge of $|GS, R\rangle$, the state with OBCs respecting $G_{\mathbf{x}}$. Although N depends on R, the lower bound is in terms of local RSIs and hence is independent of R. Shrinking R to include only orbitals at $\mathbf{x}$ produces a lower bound for the total charge N at $\mathbf{x}$.

We can systematically develop inequalities thanks to the general form of the many-body local RSI symmetry operators derived in Supplementary Note II C. To begin, we consider the spinless rotation groups which for even n have the many-body local RSIs

$$e^{i\frac{\pi}{n}\hat{N}}C_n |GS\rangle = e^{i\frac{\pi}{n}\Delta_1}|GS\rangle, \qquad C_n^2 |GS\rangle = e^{i\frac{2\pi}{n/2}\Delta_2}|GS\rangle \tag{53}$$

with $\Delta_1 \in \mathbb{Z}_{2n}, \Delta_2 \in \mathbb{Z}_{n/2}$. Noting that $(e^{i\frac{\pi}{n}\hat{N}}C_n)^2(C_n^2)^\dagger = e^{i\frac{2\pi}{n}\hat{N}}$, we see immediately that $N = \Delta_1 - 2\Delta_2 \mod n$ and thus $N \geq (\Delta_1 - 2\Delta_2 \mod n)$ where $(a \mod n)$ is taken to be non-negative. However, this inequality is not tight and can be improved. To see that it is not tight, consider $G_{\mathbf{x}} = 2$ in Supplementary Table V where $\Delta_1 = m(A) - m(B) \mod 4$ when acting on product states ($\Delta_2 = 0$ is trivial) and the inequality is $N \geq \Delta_1 \mod 2$. The only state with zero particles is the vacuum with $\Delta_1 = 0 \mod 4$ and the only one-particle states are those with an A irrep or a B irrep which have $\Delta_1 = \pm 1 \mod 4$. Thus $\Delta_1 = 2 \mod 4$ can only be obtained with 2 or more particles, e.g. $m(A) = 2$ or $m(B) = 2$, even though $\Delta_1 \mod 2 = 0$.

To tighten the bound, we prove the following. If $\Delta_1 - 2\Delta_2 = 0 \mod n$ but $\Delta_1 \neq 0$, then $N \geq n$. The proof is by contradiction. Because $\Delta_1 - 2\Delta_2 = N \mod n$, if $\Delta_1 - 2\Delta_2 = 0 \mod n$ then N must be a multiple of n. Assume for contradiction that $N = 0$, so the groundstate $|0\rangle$ is the vacuum. Then $e^{i\frac{\pi}{n}\hat{N}}C_n|0\rangle = +|0\rangle$ implying that $\Delta_1 = 0$, reaching a contradiction. Thus N is a nonzero multiple of n, so $N \geq n$.

We now discuss the case of odd n whose many-body local RSIs are $e^{\frac{2\pi i}{n}\hat{N}}|GS\rangle = e^{\frac{2\pi i}{n}\Delta_1}|GS\rangle$ and $C_n|GS\rangle = e^{\frac{2\pi i}{n}\Delta_2}|GS\rangle$. As before, we have the simple inequality $N \geq \Delta_1 \mod n$ which is not tight at $N = n$ (for example with $n = 3$, a product state with $m(A) = 1, m({}^1\mathrm{E}) = 2$ has $\Delta_1 = 0$ from Supplementary Table V). We improve bound with the same contraction argument as before: if $\Delta_1 = 0 \mod n$ but $\Delta_2 \neq 0$, then $N \mod n = 0$ but $N \neq 0$ or else $\Delta_2 = 0$.

It is now convenient to define the function

$$\mathrm{mod}_n(\Delta|\Delta') = \begin{cases} n, & \Delta \mod n = 0, \quad \Delta' \neq 0 \\ (\Delta \mod n) \in (0, \dots, n-1), & \text{otherwise} \end{cases}. \tag{54}$$

The preceding paragraphs can now be stated $N \geq \mathrm{mod}_n(\Delta_1 + 2\Delta_2|\Delta_1)$ for n even and $N \geq \mathrm{mod}_n(\Delta_1|\Delta_2)$ for n odd in the spinless rotation groups. Since we found in Supplementary Note II D that all the many-body local RSIs in the spinless 2D point groups can be obtained from their rotation subgroups by reduction $\Delta_1 \to \Delta_1, \Delta_2 \to 0$, we then obtain the bounds $N \geq \mathrm{mod}_n(\Delta_1|\Delta_1)$ in all the spinless groups with mirrors and/or time-reversal.

We now consider the spinful rotation groups where the RSIs are (see Eq. 39) $C_n|GS\rangle = e^{i\frac{\pi}{n}\Delta_1}|GS\rangle$ and $e^{i\frac{2\pi}{n}\hat{N}}C_n^2|GS\rangle = e^{i\frac{4\pi}{n}\Delta_2}|GS\rangle$ for even n. Since $e^{i\frac{2\pi}{n}\hat{N}}C_n^2(C_n^\dagger)^2|GS\rangle = e^{i\frac{2\pi}{n}\hat{N}}|GS\rangle = e^{i\frac{2\pi}{n}(2\Delta_2 - \Delta_1)}|GS\rangle$, we see that $N = 2\Delta_2 - \Delta_1 \mod n$, and by the same argument as above, we tighten the bound but noting that if $2\Delta_2 - \Delta_1 = 0 \mod n$ but $\Delta_1 \neq 0$, then $N \geq n$. The bound for groups with mirrors follows by reduction of the RSIs as shown now. Adding mirrors reduces the local RSIs (see Eq. 24) such that $N = 2\Delta_2 \mod n$. If n is odd, Eq. 40 immediately gives $N = \Delta_1 \mod n$ and $N \geq n$ if $\Delta_1 = 0 \mod n$ and $\Delta_2 \neq 0$. With mirrors, Δ_2 reduces to zero (see Eq. 43).

We now consider the addition of time-reversal symmetry. In the spinful groups with SOC, the many-body RSI $N/2 = \Delta_2 \mod n$ counts the number Kramers pairs, and $\Delta_2 = 0 \mod n, \Delta_1 \neq 0$ implies $N/2 \geq n$ for n even. For n odd, there is only one many-body local RSI Δ_1 which counts the number of Kramers pairs.

With these results, we compile the local RSI lower bounds in Supplementary Table VII.

TABLE VII: Supplementary Table 7. Local $U(1)$ Particle-number Constraints from many-body local RSIs. All mod functions are defined to be non-negative.

	No SOC ($C_n^n = M_i^2 = +1$)		SOC ($C_n^n = M_i^2 = -1$)	
	No TRS	TRS ($\mathcal{T}^2 = +1$)	No TRS	TRS ($\mathcal{T}^2 = -1$)
m				
2	$N \geq \mathrm{mod}_2(\Delta_1\|\Delta_1)$	$N \geq \mathrm{mod}_2(\Delta_1\|\Delta_1)$	$N \geq \mathrm{mod}_2(\Delta_1\|\Delta_1)$	$N/2 \geq \mathrm{mod}_2(\Delta_2\|\Delta_1)$
$2mm$	$N \geq \mathrm{mod}_2(\Delta_1\|\Delta_1)$	$N \geq \mathrm{mod}_2(\Delta_1\|\Delta_1)$	$N \geq \mathrm{mod}_2(0\|\Delta_1)$	$N/2 \geq \mathrm{mod}_2(\Delta_2\|\Delta_1)$
4	$N \geq \mathrm{mod}_4(\Delta_1 - 2\Delta_2\|\Delta_1)$	$N \geq \mathrm{mod}_4(\Delta_1\|\Delta_1)$	$N \geq \mathrm{mod}_4(2\Delta_2 - \Delta_1\|\Delta_1)$	$N/2 \geq \mathrm{mod}_4(\Delta_2\|\Delta_1)$
$4mm$	$N \geq \mathrm{mod}_4(\Delta_1\|\Delta_1)$	$N \geq \mathrm{mod}_4(\Delta_1\|\Delta_1)$	$N \geq \mathrm{mod}_4(2\Delta_2\|\Delta_1)$	$N/2 \geq \mathrm{mod}_4(\Delta_2\|\Delta_1)$

3	$N \geq \text{mod}_3(\Delta_1\|\Delta_2)$	$N \geq \text{mod}_3(\Delta_1\|\Delta_1)$	$N \geq \text{mod}_3(\Delta_1\|\Delta_2)$	$N/2 \geq \text{mod}_3(\Delta_1\|\Delta_1)$
$3m$	$N \geq \text{mod}_3(\Delta_1\|\Delta_1)$	$N \geq \text{mod}_3(\Delta_1\|\Delta_1)$	$N \geq \text{mod}_3(\Delta_1\|\Delta_1)$	$N/2 \geq \text{mod}_3(\Delta_1\|\Delta_1)$
6	$N \geq \text{mod}_6(\Delta_1 - 2\Delta_2\|\Delta_1)$	$N \geq \text{mod}_6(\Delta_1\|\Delta_1)$	$N \geq \text{mod}_6(2\Delta_2 - \Delta_1\|\Delta_1)$	$N/2 \geq \text{mod}_6(\Delta_2\|\Delta_1)$
$6mm$	$N \geq \text{mod}_6(\Delta_1\|\Delta_1)$	$N \geq \text{mod}_6(\Delta_1\|\Delta_1)$	$N \geq \text{mod}_6(2\Delta_2\|\Delta_1)$	$N/2 \geq \text{mod}_6(\Delta_2\|\Delta_1)$

C. Defining Many-Body Local RSIs in Fragile States

We now explain how to define the many-body local RSIs of a (many-body) fragile topological state following the method of the Main Text. First we recall that the many-body local RSIs are defined on symmetry-respecting OBCs in many-body trivial atomic limits. Crucially, such phases are non-degenerate on OBCs, while fragile phases (or obstructed atomic limit phases) *are* degenerate at filling $\nu = N_{occ}/N_{orb}$. Thus we cannot *directly* calculate the many-body local RSIs of fragile states by placing them on OBCs.

Instead, we define many-body local RSIs using the definition of a many-body fragile phase: although one cannot adiabatically deform such a state to a many-body atomic state at fixed particle number, an adiabatic deformation is possible when coupled to a many-body atomic limit (thereby increasing the particle number). This definition encompasses single-particle fragile phases[3]. To start with, we assume PBCs so we only need to consider the non-triviality of the bulk (not the edges or corners). Explicitly, we denote the fragile state by $|F\rangle = F^\dagger |0\rangle$ which is trivialized through the addition of a trivial atomic limit state $|A\rangle = A^\dagger |0\rangle$. By trivialized, we mean that $A^\dagger F^\dagger |0\rangle$ can be adiabatically deformed to a many-body trivial atomic state $|A'\rangle$ at fixed particle number. If necessary, new orbitals may be added to the Hilbert space to define A. This is only required in a tight-binding model where there is a finite local Hilbert space (as opposed to a continuum model). The set of many-body local RSIs of $|A\rangle$ and $|A'\rangle$ are well-defined and are denoted by Δ_A and $\Delta_{A'}$ respectively. Then because the many-body local RSI groups are abelian (they are additive under stacking as discussed in the Main Text), we define the RSIs of the fragile state by $\Delta_F = \Delta_{A'} - \Delta_A$. Note that Δ_F is invariant under $A^\dagger \to A^\dagger B^\dagger$ for any many-body atomic state $B^\dagger$ because $\Delta_{A'B} - \Delta_{AB} = \Delta_{A'} + \Delta_B - \Delta_A - \Delta_B = \Delta_{A'} - \Delta_A$. It appears that Δ_F depends on the atomic state $|A\rangle$ used to trivialize $|F\rangle$ rather than being an intrinsic property of $|F\rangle$. However, we can prove that Δ_F is unique and does not depend on A because the RSI groups are abelian. Consider two distinct operators $A^\dagger$, $\tilde{A}^\dagger$ for which $F^\dagger A^\dagger |0\rangle, F^\dagger \tilde{A}^\dagger |0\rangle$ are both many-body atomic with corresponding RSIs $\Delta_F = \Delta_{FA} - \Delta_A, \Delta_{\tilde{F}} = \Delta_{F\tilde{A}} - \Delta_{\tilde{A}}$ respectively. Then $F^\dagger A^\dagger \tilde{A}^\dagger |0\rangle$ is many-body atomic, and its many-body local RSIs $\Delta_{FAA'} - \Delta_{AA'}$ are equal to Δ_F and $\Delta_{\tilde{F}}$, since the RSIs of the fragile state are invariant under the addition of atomic states as mentioned in the prior paragraph. Hence $\Delta_F = \Delta_{\tilde{F}}$. It is worth underscoring that A' must be a trivial atomic state rather than an obstructed atomic state. To understand this requirement, recall that in non-interacting Hamiltonians, the orbitals used to trivialize fragile bands may result in obstructed atomic bands. That is insufficient in our framework for computing the many-body local RSIs on OBCs because obstructed atomic bands host corner states. Momentarily, we will discuss how adding *more* (appropriately chosen) atomic orbitals can resolve the obstruction and thereby obtain a trivial atomic limit.

Although the many-body local RSIs defined here do not depend on which many-body atomic operator A is used to trivialize $|F\rangle$, it is necessary to find a known one in order to actually perform a computation. We now outline a strategy to find A in the band theory limit where single-particle fragile states have been classified[3,6]. On PBCs, fragile phases can be represented in the form $(\rho \ominus \rho') \uparrow G$ where ρ, ρ' represent atomic orbitals in the unit cell (see Refs.[3,6] for details and the Main Text for an example). The obstruction to trivialization is represented by $\ominus\rho'$, and can be removed on PBCs by coupling orbitals ρ' to the fragile band (without closing a gap). However, it is then possible that ρ is an obstructed atomic state: it is consistent with the symmetries of an atomic state, but not one that appears in the tight-binding orbital basis. On OBCs, such an obstructed atomic state has a filling anomaly[7] which enforces degeneracy, preventing us from calculating the many-body local RSIs. However, we will now show that an obstructed atomic state can be connected to a trivial atomic state (at different particle number) by taking *more* orbitals in the choice of A, so that non-degenerate OBCs are obtained upon truncating the PBC model. We explain this first on PBCs by showing how to resolve the bulk obstruction to an atomic limit. Afterwards, we give a complementary discussion on OBCs with a numerical example.

On PBCs, an obstructed atomic limit is defined by the representation $\rho_O \uparrow G$ which is compatible with atomic orbitals, but *not* those which appear in the basis of the tight-binding model. This obstruction is removed simply by adding atomic orbitals in the representation ρ_O to the tight-binding basis. This is intuitive because the "obstruction" of an obstructed atomic limit state is related to the basis of atomic orbitals in the tight-binding Hamiltonian. By adding the correct orbitals to this basis, the obstruction is resolved. Explicitly, we consider an obstructed atomic

insulator adiabatically tuned to the flat band limit where the Hamiltonian can be written on PBCs as

$$H_{OAI} = -t \sum_{\mathbf{R},n\in\rho_O} w^\dagger_{\mathbf{R},n} w_{\mathbf{R},n} \tag{55}$$

where $w^\dagger_{\mathbf{R},n}$ are the Wannier functions carrying the representation of the nth orbital in ρ_O. In H_{OAI}, the obstructed valence bands are at energy $-t < 0$, and the conduction bands are at energy 0 (thus their creation operators do not appear in the Hamiltonian). To trivialize the obstructed atomic limit, we add atomic $c^\dagger_{\mathbf{R},n}$ orbitals transforming in the same representation at $w^\dagger_{\mathbf{R},n}$. We propose the new Hamiltonian $H(\theta)$ on PBCs

$$\begin{aligned} H(\theta) &= -t\cos\theta \sum_{\mathbf{R},n\in\rho_O} w^\dagger_{\mathbf{R},n} w_{\mathbf{R},n} + t\cos\theta \sum_{\mathbf{R},n\in\rho_O} c^\dagger_{\mathbf{R},n} c_{\mathbf{R},n} + t\sin\theta \left(\sum_{\mathbf{R},n\in\rho_O} w^\dagger_{\mathbf{R},n} c_{\mathbf{R},n} + h.c. \right) \\ &= -t \sum_{\mathbf{R},n\in\rho_O} \begin{pmatrix} w^\dagger_{\mathbf{R},n} & c^\dagger_{\mathbf{R},n} \end{pmatrix} (\sigma_z \cos\theta + \sigma_x \sin\theta) \begin{pmatrix} w_{\mathbf{R},n} \\ c_{\mathbf{R},n} \end{pmatrix} \end{aligned} \tag{56}$$

whose spectrum is $\pm t, 0$ for all θ. Note that the coupling $w^\dagger_{\mathbf{R},n} c_{\mathbf{R},n}$ is symmetry-preserving. At $\theta = 0$, $H(0)$ has the same spectrum as H_{OAI} but with additional conduction bands of $c^\dagger_{\mathbf{R},n}$ orbitals at energy $+t$. Tuning θ to π does not close the gap (the spectrum is independent of θ), and at $\theta = \pi$ where the Wannier-orbital coupling $t\sin\theta$ vanishes, the Wannier states $w^\dagger_{\mathbf{R},n}$ are now in the conduction band at energy $+t$ and the trivial orbitals $c^\dagger_{\mathbf{R},n}$ are in the valence band. Hence this model tunes between the obstructed atomic state in the original Hilbert space and a trivial atomic limit in the expanded Hilbert space without a gap closing on PBCs.

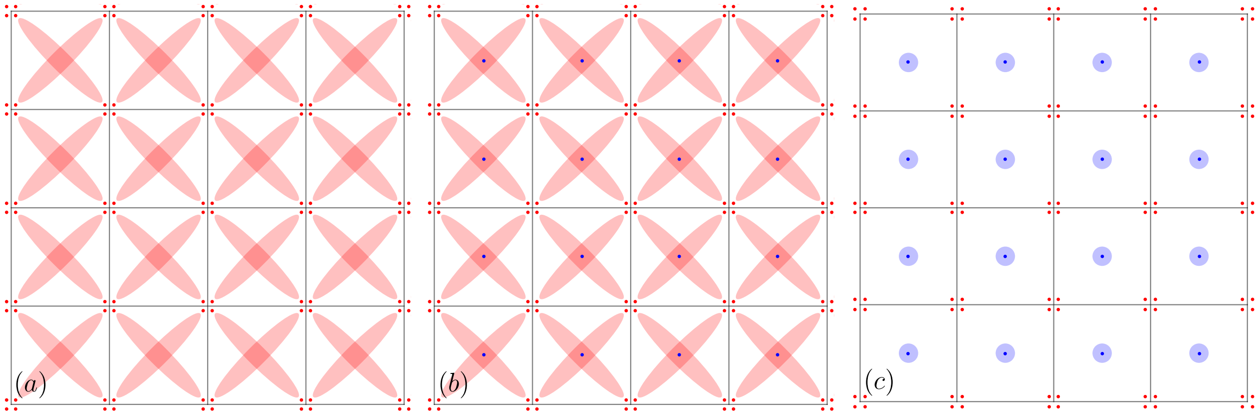

FIG. 2. Supplementary Figure 2. Flat band obstructed model and auxiliary atomic orbitals. (a) We show the original 4-orbital model. The four small red points by the 1a position denote the $A, B, {}^1E^2E$ orbitals of the tight-binding basis. The A_{1b} Wannier function is shown as a large four-pronged wavefunction to emphasize its support on the four neighboring 1a sites. On OBCs, the spatial extend of the Wannier function is responsible for corner states. (b) The auxiliary A_{1b} atomic orbital is added to the tight-binding basis shown in blue. It transforms in the same way as the A_{1b} Wannier state. (c) By coupling the Wannier state to the auxiliary atomic state as in Eq. 56, there is an adiabatic path on PBCs between the initial red Wannier state supported over multiple unit cells, and the final blue atomic orbitals which is delta-function supported.

We have shown how to resolve the bulk obstruction to a trivial atomic limit on PBCs by adiabatically deforming the obstructed Wannier functions into auxiliary atomic orbitals $c^\dagger_{\mathbf{R},n}$ via Eq. 56. The many-body local RSIs are manifestly well defined in the trivial atomic limit (upon imposing OBCs), so we have shown by construction that the many-body local RSIs of obstructed states may be defined following this procedure. However, it is instructive to track the evolution of $H(\theta)$ on OBCs. We do so by using a flat band obstructed atomic limit Hamiltonian with space group $p41'$ defined in **??**. The model of **??** is a four-orbital model with a basis of $A, B, {}^1E^2E$ orbitals at the 1a position depicted in Supplementary Figure 2a. The model (see Eq. 57) is constructed to have a compact Wannier state in the A_{1b} irrep (an s-orbital at the $(1/2, 1/2)$ position) at energy $E = -1$. This Wannier state is obstructed because the orbital basis consists of orbitals at the 1a position (not at 1b). The three flat conduction bands are at energy $E = 0$. Thus the bulk gap $E \in (-1, 0)$ realizes an obstructed atomic phase in the four band model. We now

add a A_{1b} *atomic* orbital to the Hilbert space which will trivialize the obstructed phase. Explicitly, single-particle Hamiltonian is written in the ordered basis $A_{1a}, B_{1a}, ({}^1E^2E)_{1a}, A_{1b}$ as

$$h(\theta) = -U(\mathbf{k})U(\mathbf{k})^\dagger \cos\theta + V(\mathbf{k})V(\mathbf{k})^\dagger \cos\theta + (U(\mathbf{k})V(\mathbf{k})^\dagger + V(\mathbf{k})U(\mathbf{k})^\dagger)\sin\theta$$

$$U(\mathbf{k}) = \frac{1}{4}\begin{pmatrix}1\\1\\1\\1\\0\end{pmatrix} + \frac{1}{4}e^{-i\mathbf{k}\cdot\mathbf{a}_1}\begin{pmatrix}1\\-1\\-i\\i\\0\end{pmatrix} + \frac{1}{4}e^{-i\mathbf{k}\cdot(\mathbf{a}_1+\mathbf{a}_2)}\begin{pmatrix}1\\1\\-1\\-1\\0\end{pmatrix} + \frac{1}{4}e^{-i\mathbf{k}\cdot\mathbf{a}_2}\begin{pmatrix}1\\-1\\i\\-i\\0\end{pmatrix}, \quad V(\mathbf{k}) = e^{-i\mathbf{k}\cdot(\mathbf{a}_1+\mathbf{a}_2)/2}\begin{pmatrix}0\\0\\0\\0\\1\end{pmatrix} \tag{57}$$

so that $U(\mathbf{k})$ creates a compact Wannier function at 1b $= (1/2, 1/2)$ supported on the nearest four 1a sites, and $V(\mathbf{k})$ creates a trivial atomic orbital 1b $= (1/2, 1/2)$ (see **??** for details) shown in Supplementary Figure 2b. By coupling the Wannier state to the atomic state (on PBCs at fixed filling $\nu = 1/5$, noting that the addition of the A_{1b} orbital means there are five orbitals in the unit cell), we trivialize the obstructed state by adiabatically connecting it to the trivial atomic phase symbolized in Supplementary Figure 2c. We now discuss this model numerically on OBCs to understand how the filling anomaly is resolved.

When discussing OBCs, the precise choice of boundaries is important if there are orbitals at different Wyckoff positions in the unit cell. We depict three choices in Supplementary Figure 3. Both Supplementary Figure 3a and Supplementary Figure 3c depict C_4-preserving boundaries centered at the 1a and 1b positions respectively, while Supplementary Figure 3b shows a choice of boundaries that breaks C_4 but preserves the unit cell. Let us discuss the geometry of these cases in detail. To preserve C_4 symmetry at the 1a position (as in Supplementary Figure 3a), we center the boundary around 1a and choose a C_4 symmetric cutoff. Necessarily, there will be $1 + 4\mathbb{N}$ 1a sites, since the 1a site at the center transforms to itself, whereas all other 1a sites come in quartets. Similarly since the 1b position is off the center, there must be $4\mathbb{N}$ 1b positions. In Supplementary Figure 3a, we chose $L^2 = 25 = 1 + 6 \times 4$ 1a sites, and $N_{1b} \equiv (L-1)^2 = 16 = 4 \times 4$ 1b sites for $L = 5$ odd. In Supplementary Figure 3c where the 1b site is at the center, we chose $L^2 = 16 = 4 \times 4$ 1a sites, and $N_{1b} \equiv (L+1)^2 = 25 = 1 + 6 \times 4$ 1b sites for $L = 4$ even. When computing the many-body local RSIs, we must preserve the symmetries that protect them on OBCs, so Supplementary Figure 3a,c are suitable while Supplementary Figure 3b is not. We remark that the $L \times L$ OBC of Supplementary Figure 3b is a commonly chosen boundary truncation in numerics because it preserves the unit cell, and hence the *filling*, for any L although it breaks the C_4 symmetry.

We now discuss the OBC spectrum for the C_4-preserving OBCs in Supplementary Figure 3a,c. We showed in this Supplementary Note that by adding auxiliary orbitals, we were able to connect the obstructed atomic limit to a trivial atomic limit of A_{1b} orbitals on PBCs. Thus on OBCs, we always focus on the gap above occupying N_{1b} states, which corresponds to the trivial atomic limit of A_{1b} orbitals where we first defined many-body local RSIs (see Supplementary Note II C). We will see that at this particle number $N = N_{1b}$, there is no filling anomaly (which we discuss in detail momentarily). By tuning θ in Eq. 56, we study the adiabatic connection between the trivial atomic orbitals of $H(\pi)$ and the initial obstructed Wannier states of $H(0)$.

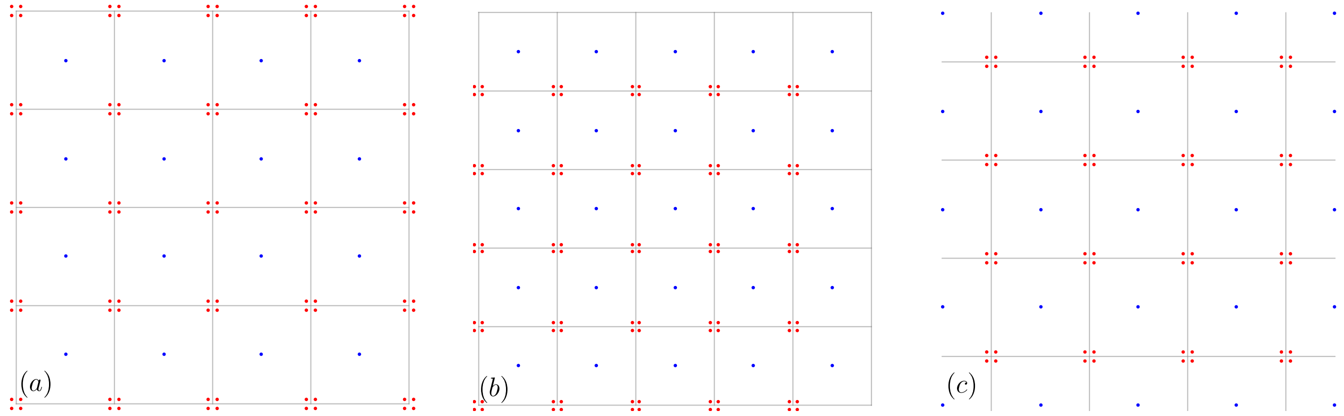

FIG. 3. Supplementary Figure 3. Different OBC boundary conditions. (a) We preserve the C_4 symmetry of the 1a position as in Supplementary Figure 4a below. (b) C_4 symmetry is broken, but the unit cell is preserved such that there are the same number of 1a and 1b positions. (c) We preserve the C_4 symmetry of the 1b position as in Supplementary Figure 4c below.

Supplementary Figure 4 shows the OBC spectrum of $H(\theta)$ (Eq. 56) for OBCs centered at 1a and 1b, explicitly

verifying the adiabatic path at constant particle number N_b between the obstructed phase of the original Hilbert space (which is no longer obstructed when the A_{1b} orbital is added to the Hilbert space) and the trivial phase. Let us elaborate on how this has resolved the filling anomaly of the original four orbital model in Eq. 55. One important feature of Eq. 55 is that all orbitals are at the 1a position, so a C_4-preserving boundary condition as in Supplementary Figure 3a will preserve the unit cell. Then at filling $\nu = 1/4$ (the bulk filling of the obstructed Wannier state) the groundstate is degenerate due to the filling anomaly. To verify this, we check in Supplementary Figure 4a for $H(\theta = 0)$ (where the auxiliary A_{1b} orbitals are decoupled and do not effect the numerics) that occupying L^2 states (which is 1/4 of the $4L^2$ orbitals at $1a$) leads to a degenerate groundstate occupying half of the four corner states at $E = -.25$. However, we showed that on these OBCs, the trivial atomic limit of $H(\theta = \pi)$ occurs at occupying $N_{1b} = (L-1)^2$ states. We check that this corresponds to full filling of the $E = -1$ states and is non-degenerate with a gap above to the edge states at $E = -.5$. Of course, we could have chosen a different C_4-respecting boundary cutoff that also included the nearest 1b sites outside of the L^2 1a sites (in Supplementary Figure 3a, this would correspond to having $6^2 = 36$ 1b sites in 1b, enlarged from $4^2 = 16$). Then the trivial atomic limit would be obtained at occupying $(L+1)^2$ states, which we verify in Supplementary Figure 4a at $\theta = 0$ corresponds to occupying all the edge and corner states in addition to the bulk states, leading to a non-degenerate state with a gap to the bulk states at $E = 0$. We see that it is the combination of symmetry-preserving boundary conditions and the number of occupying states fixed by the unit cell geometry of the auxiliary orbital that resolves the OBC filling anomaly.

It is interesting to note that the edge states and corner states, which appear in the $E \in (-1, 0)$ bulk gap at $\theta = 0$ due to the obstructed Wannier state, do not contribute to the many-body local RSI. This is because they are strictly supported on the boundary and can be represented as $\prod_{i=1}^4 C_4^i \mathcal{O}_{bdy} C_4^{i\dagger}$ where $\mathcal{O}_{bdy}$ is the creation operator for all the boundary states on a single edge and corner. The many-body local RSIs are $\Delta_{1a} = (0 \mod 8, 0 \mod 2), \Delta_{1b} = (1 \mod 8, 0 \mod 2), \Delta_{2c} = 0 \mod 4$ from Supplementary Table V and can be obtained immediately from the atomic limit state at $\theta = \pi$.

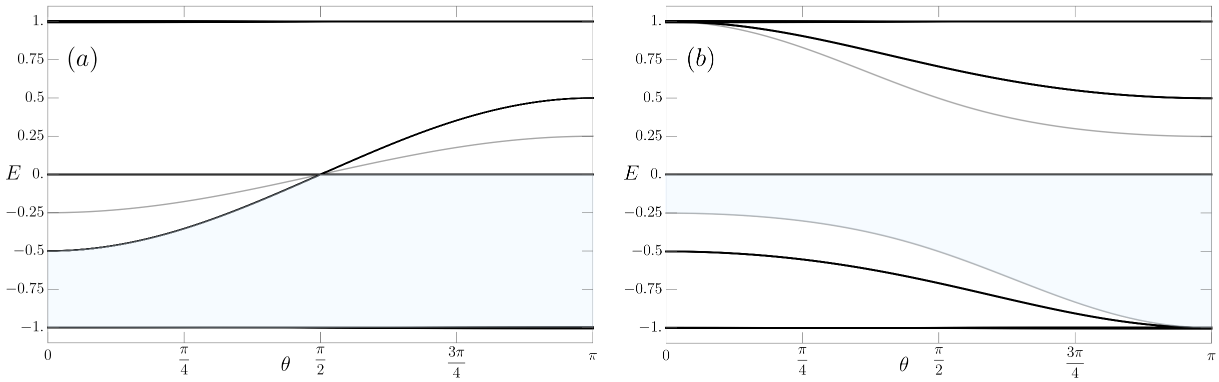

FIG. 4. Supplementary Figure 4.Adiabatic connection between an obstructed atomic phase and trivial atomic phase via the addition of trivial orbitals to the Hilbert space. Corner states originating from $E = -1/4$ appear in gray, and the edge states originating from $E = -1/2$ are dark gray. (a) We fix the center to be at the 1a position and choose OBCs to have L^2 1a sites (each with 4 orbitals) and $N_{1b} = (L-1)^2$ 1b sites for $L = 23$ odd. The gap above the $N_{1b} = 484$ state is shaded light blue. (b) We fix the center to be at the 1b position and choose OBCs to have L^2 1a sites (each with 4 orbitals) and $N_{1b} = (L+1)^2$ 1b sites for $L = 22$ even. The gap above the $N_{1b} = 529$ state is shaded light blue.

D. Fragile Inequalities

We now study how the particle number constraints in Supplementary Table VII at each Wyckoff in the unit cell can be used to diagnose many-body fragile topology. In the non-interacting case, the classification of fragile phases has an affine monoid structure[3,6] because the single-particle RSIs could be $\mathbb{Z}$-valued, i.e. there were infinite possible RSIs. In this (single-particle) case, the orbital number/RSI constraints analogous to those in Supplementary Table VII included inequality constraints and $\mathbb{Z}_2$ constraints.

In the interacting case where the many-body RSIs only take finitely many values, the fragile criteria are actually easier to derive. Our strategy is to devise inequality criteria which hold in all many-body *atomic* states in terms of the local bounds RSIs (protected by the PG symmetries) and the total particle number (protected by the global $U(1)$ symmetry). If the a given set of local RSIs *violate* the inequality criteria, then there is an obstruction to deformation into a many-body atomic state, proving many-body fragile topology.

In a many-body atomic limit, the number of particles $N_{\mathbf{x}}$ at each site $\mathbf{x} = \mathbf{R}+\mathbf{r}$ is well-defined by $[\hat{N}, \mathcal{O}_{\mathbf{R},\mathbf{r}}] = N_{\mathbf{x}}\mathcal{O}_{\mathbf{R},\mathbf{r}}$ using the notation in Supplementary Note II C where $\mathcal{O}_{\mathbf{R},\mathbf{r}}$ is the creation operator of the groundstate of $H_{\mathbf{R},\mathbf{r}}$. By translation symmetry, $N_{\mathbf{R}+\mathbf{r}} = N_{\mathbf{r}}$ and we need only consider a single unit cell. At integer filling $\nu = N_{occ}/N_{orb}$, the total number of particles per unit cell N_{occ} is obtained by adding up the states at all the Wyckoff positions in the unit cell:

$$N_{occ} = \sum_{\mathbf{x}} m_{\mathbf{x}} N_{\mathbf{x}} \geq \sum_{\text{high-symmetry } \mathbf{x}} m_{\mathbf{x}} N_{\mathbf{x}} \qquad \text{(many-body atomic)} \tag{58}$$

where $m_{\mathbf{x}}$ is the multiplicity of the Wyckoff position $\mathbf{x}$ (the many-body local RSIs at each point in the Wyckoff position are equal by Eq. 27 because they are related by symmetries in G). Note that the sum in the second equality of Eq. 58 is over high-symmetry Wannier positions where the local RSIs are nontrivial, i.e. $G_{\mathbf{x}} \neq 1$. Using Supplementary Table VII, $N_{\mathbf{x}}$ can be lower bounded by the many-body RSIs at $\mathbf{x}$, which remain well-defined in many-body fragile topological phases. N_{occ} is also a well-defined many-body quantity: it is the $U(1)$ number density $N_{occ} = N/L^2$ where N is the total number of particles in the groundstate and L^2 is the number of unit cells. Schematically, we write

$$N_{occ} \geq \sum_{\text{high-symmetry } \mathbf{x}} m_{\mathbf{x}} N_{\mathbf{x}} \geq \sum_{\text{high-symmetry } \mathbf{x}} m_{\mathbf{x}} \, \text{bound}_{\mathbf{x}}(\Delta) \tag{59}$$

with explicit expressions for the many-body local RSIs bound $\text{bound}_{\mathbf{x}}(\Delta)$ are tabulated in Supplementary Table VII. Eq. 59 is obeyed in all many-body atomic states. If Eq. 59 is *violated*, then the many-body local RSIs impose an obstruction to deformation into a many-body atomic state, which defines many-body fragile topology. Hence the many-body fragile topological indices are inequalities corresponding to the violation of Eq. 59:

$$N_{occ} < \sum_{\text{high-symmetry } \mathbf{x}} m_{\mathbf{x}} \, \text{bound}_{\mathbf{x}}(\Delta) \qquad \text{(Many-body fragile)} \ . \tag{60}$$

In Supplementary Tables VIII and IX, we write down the fragile criteria of Eq. 60 in all space groups.

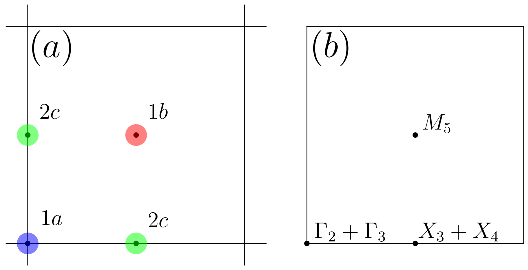

FIG. 5. Supplementary Figure 5. (a) The high-symmetry Wyckoff positions in the unit cell are 1a ($4mm$) in blue, 1b ($4mm$) in red and 2c ($2mm$) in green. The 1a and 1b Wyckoff positions have multiplicity 1, and 2c has multiplicity 2. (b) We show the band structure of a single-particle fragile phase with is also many-body fragile. The high-symmetry points in the BZ are Γ ($4mm$), M ($4mm$), and X ($2mm$).

We now give an example in the wallpaper group $p4mm$ which is generated by C_4, M_y, and translations. The high-symmetry Wyckoff positions are shown in Supplementary Figure 5. We do not show the mirror-symmetric lines. The 1a and 1b positions have $G_{\mathbf{x}} = 4mm$, and the 2c position has $G_{\mathbf{x}} = 2mm$. The many-body RSI bounds for $m_{\mathbf{x}} N_{\mathbf{x}}$ from Supplementary Table VII are

$$\begin{aligned} 4mm: &\qquad N_{1a} \geq \text{mod}_4(\Delta_{1a,1}|\Delta_{1a,1}), \\ 4mm: &\qquad N_{1b} \geq \text{mod}_4(\Delta_{1b,1}|\Delta_{1b,1}), \\ 2mm: &\qquad 2N_{2c} \geq 2\text{mod}_2(\Delta_{2c,1}|\Delta_{2c,1}) \ . \end{aligned} \tag{61}$$

Hence in any many-body atomic state,

$$N_{occ} \geq \text{mod}_4(\Delta_{1a,1}|\Delta_{1a,1}) + \text{mod}_4(\Delta_{1b,1}|\Delta_{1b,1}) + 2\text{mod}_2(\Delta_{2c,1}|\Delta_{2c,1}) \ . \tag{62}$$

Violating this inequality is an obstruction to the many-body atomic limit, so we deduce

$$N_{occ} < \text{mod}_4(\Delta_{1a,1}) + \text{mod}_4(\Delta_{1b,1}) + 2\text{mod}_2(\Delta_{2c,1}) \qquad \Longrightarrow \text{ many-body fragile topology} \ . \tag{63}$$

We now give an example of a single-particle state which is many-body fragile. ?? contains a list of fragile roots that generate all fragile band structures in a given space group. We choose the fragile band structure $\mathcal{B} = \Gamma_2 + \Gamma_3 + M_5 + X_3 + X_4$ which has $N_{occ} = 2$. The Γ_2, Γ_3, and M_5 irreps are isomorphic to the B_1, B_2, and E irreps of $4mm$ respectively in momentum space (see Supplementary Table I), and the X_3, X_4 irreps are isomorphic to the B_1, B_2 irreps of PG $2mm$ (see Supplementary Table I). The single-particle RSIs of ?? are computed from $\mathcal{B}$ to be

$$(\delta_1^{1a}, \delta_2^{1a}) = (1,1), \quad (\delta_1^{1b}, \delta_2^{1b}) = (0,1), \quad \delta_1^{2c} = -1 \tag{64}$$

and from Supplementary Table VI, we compute the many-body RSIs by reducing the single-particle RSIs

$$\Delta_1^{1a} = 7 \mod 8, \quad \Delta_1^{1b} = 5 \mod 8, \quad \Delta_1^{2c} = 1 \mod 4\ . \tag{65}$$

We evaluate the many-body fragile criteria in Eq. 63,

$$N_{occ} < \text{mod}_4(\Delta_{1a,1}|\Delta_{1a,1}) + \text{mod}_4(\Delta_{1b,1}|\Delta_{1b,1}) + 2\text{mod}_2(\Delta_{2c,1}|\Delta_{2c,1}) = 3 + 1 + 2(1) = 6 \tag{66}$$

from which we see that the state $\mathcal{B}$ is many-body fragile since $N_{occ} = 2$. We can reproduce this calculation directly in real space checking that

$$\mathcal{B} = (E^{1a} \oplus B_1^{1a} \oplus B_2^{1b} \ominus B_1^{2c}) \uparrow p4mm \tag{67}$$

using the band representations on the Bilbao Crstallographic Server (`https://www.cryst.ehu.es/cgi-bin/cryst/programs/bandrep.pl`). We can directly determine the many-body RSIs in real space using Supplementary Table V:

$$\Delta_1^{1a} = -3 + 2 \mod 8, \quad \Delta_1^{1b} = -3 \mod 8, \quad \Delta^{2c} = 1 \mod 4\ . \tag{68}$$

which matches the momentum space result. The intuition from the real space representation in Eq. 67 is that the fragile topology results from the $\ominus B_1^{2c}$ obstruction which – in this particular state – cannot be removed by an interaction-enabled conversion. Supplementary Note V A details an exactly solvable Hamiltonian where the $\ominus$ irrep obstruction can be removed by interactions, as diagnosed by the many-body local RSIs.

TABLE VIII: Supplementary Table 8. Fragile Criteria (No SOC)

SG	WP	$N_{occ} < \dots$
$p1$	$a(1)$	
$p1'$	$a(1')$	
$p2$	$1a(2)$	$\text{mod}_2(\Delta_{1a,1}\|\Delta_{1a,1})$
	$1b(2)$	$+\text{mod}_2(\Delta_{1b,1}\|\Delta_{1b,1})$
	$1c(2)$	$+\text{mod}_2(\Delta_{1c,1}\|\Delta_{1c,1})$
	$1d(2)$	$+\text{mod}_2(\Delta_{1d,1}\|\Delta_{1d,1})$
$p21'$	$1a(21')$	$\text{mod}_2(\Delta_{1a,1}\|\Delta_{1a,1})$
	$1b(21')$	$+\text{mod}_2(\Delta_{1b,1}\|\Delta_{1b,1})$
	$1c(21')$	$+\text{mod}_2(\Delta_{1c,1}\|\Delta_{1c,1})$
	$1d(21')$	$+\text{mod}_2(\Delta_{1d,1}\|\Delta_{1d,1})$
pm	$1a(m)$	
	$1b(m)$	
$pm1'$	$1a(m1')$	
	$1b(m1')$	
pg		
$pg1'$		
cm	$2a(m)$	
$cm1'$	$2a(m1')$	
$p2mm$	$1a(2mm)$	$\text{mod}_2(\Delta_{1a,1}\|\Delta_{1a,1})$
	$1b(2mm)$	$+\text{mod}_2(\Delta_{1b,1}\|\Delta_{1b,1})$
	$1c(2mm)$	$+\text{mod}_2(\Delta_{1c,1}\|\Delta_{1c,1})$
	$1d(2mm)$	$+\text{mod}_2(\Delta_{1d,1}\|\Delta_{1d,1})$
$p2mm1'$	$1a(2mm1')$	$\text{mod}_2(\Delta_{1a,1}\|\Delta_{1a,1})$
	$1b(2mm1')$	$+\text{mod}_2(\Delta_{1b,1}\|\Delta_{1b,1})$
	$1c(2mm1')$	$+\text{mod}_2(\Delta_{1c,1}\|\Delta_{1c,1})$
	$1d(2mm1')$	$+\text{mod}_2(\Delta_{1d,1}\|\Delta_{1d,1})$
$p2mg$	$2a(2)$	$2\text{mod}_2(\Delta_{2a,1}\|\Delta_{2a,1})$
	$2b(2)$	$+2\text{mod}_2(\Delta_{2b,1}\|\Delta_{2b,1})$
	$4c(m)$	
$p2mg1'$	$2a(21')$	$2\text{mod}_2(\Delta_{2a,1}\|\Delta_{2a,1})$
	$2b(21')$	$+2\text{mod}_2(\Delta_{2b,1}\|\Delta_{2b,1})$
	$4c(m1')$	
$p2gg$	$2a(2)$	$2\text{mod}_2(\Delta_{2a,1}\|\Delta_{2a,1})$
	$2b(2)$	$+2\text{mod}_2(\Delta_{2b,1}\|\Delta_{2b,1})$
$p2gg1'$	$2a(21')$	$2\text{mod}_2(\Delta_{2a,1}\|\Delta_{2a,1})$
	$2b(21')$	$+2\text{mod}_2(\Delta_{2b,1}\|\Delta_{2b,1})$
$c2mm$	$2a(2mm)$	$2\text{mod}_2(\Delta_{2a,1}\|\Delta_{2a,1})$
	$2b(2mm)$	$+2\text{mod}_2(\Delta_{2b,1}\|\Delta_{2b,1})$
	$4c(2)$	$+4\text{mod}_2(\Delta_{4c,1}\|\Delta_{4c,1})$
$c2mm1'$	$2a(2mm1')$	$2\text{mod}_2(\Delta_{2a,1})$
	$2b(2mm1')$	$+2\text{mod}_2(\Delta_{2b,1})$
	$4c(21')$	$+4\text{mod}_2(\Delta_{4c,1})$

$p4$	$1a(4)$	$\mathrm{mod}_4(\Delta_{1a,1}-2\Delta_{1a,2}\vert\Delta_{1a,1})$
	$1b(4)$	$+\mathrm{mod}_4(\Delta_{1b,1}-2\Delta_{1b,2}\vert\Delta_{1b,1})$
	$2c(2)$	$+2\mathrm{mod}_2(\Delta_{2c,1}\vert\Delta_{2c,1})$
$p41'$	$1a(41')$	$\mathrm{mod}_4(\Delta_{1a,1}\vert\Delta_{1a,1})$
	$1b(41')$	$+\mathrm{mod}_4(\Delta_{1b,1}\vert\Delta_{1b,1})$
	$2c(21')$	$+2\mathrm{mod}_2(\Delta_{2c,1}\vert\Delta_{2c,1})$
$p4mm$	$1a(4mm)$	$\mathrm{mod}_4(\Delta_{1a,1}\vert\Delta_{1a,1})$
	$1b(4mm)$	$+\mathrm{mod}_4(\Delta_{1b,1}\vert\Delta_{1b,1})$
	$2c(2mm)$	$+2\mathrm{mod}_2(\Delta_{2c,1}\vert\Delta_{2c,1})$
$p4mm1'$	$1a(4mm1')$	$\mathrm{mod}_4(\Delta_{1a,1}\vert\Delta_{1a,1})$
	$1b(4mm1')$	$+\mathrm{mod}_4(\Delta_{1b,1}\vert\Delta_{1b,1})$
	$2c(2mm1')$	$+2\mathrm{mod}_2(\Delta_{2c,1}\vert\Delta_{2c,1})$
$p4gm$	$2a(4)$	$2\mathrm{mod}_4(\Delta_{2a,1}-2\Delta_{2a,2}\vert\Delta_{2a,1})$
	$2b(2mm)$	$+2\mathrm{mod}_4(\Delta_{2b,1}\vert\Delta_{2b,1})$
$p4gm1'$	$2a(41')$	$2\mathrm{mod}_4(\Delta_{2a,1}\vert\Delta_{2a,1})$
	$2b(2mm1')$	$+2\mathrm{mod}_4(\Delta_{2b,1}\vert\Delta_{2b,1})$
$p3$	$1a(3)$	$\mathrm{mod}_3(\Delta_{1a,1}\vert\Delta_{1a,2})$
	$1b(3)$	$+\mathrm{mod}_3(\Delta_{1b,1}\vert\Delta_{1b,2})$
	$1c(3)$	$+\mathrm{mod}_3(\Delta_{1c,1}\vert\Delta_{1c,2})$
$p31'$	$1a(31')$	$\mathrm{mod}_3(\Delta_{1a,1}\vert\Delta_{1a,1})$
	$1b(31')$	$+\mathrm{mod}_3(\Delta_{1b,1}\vert\Delta_{1b,1})$
	$1c(31')$	$+\mathrm{mod}_3(\Delta_{1c,1}\vert\Delta_{1c,1})$
$p3m1$	$1a(3m)$	$\mathrm{mod}_3(\Delta_{1a,1}\vert\Delta_{1a,1})$
	$1b(3m)$	$+\mathrm{mod}_3(\Delta_{1b,1}\vert\Delta_{1b,1})$
	$1c(3m)$	$+\mathrm{mod}_3(\Delta_{1c,1}\vert\Delta_{1c,1})$
$p3m1'$	$1a(3m1')$	$\mathrm{mod}_3(\Delta_{1a,1}\vert\Delta_{1a,1})$
	$1b(3m1')$	$+\mathrm{mod}_3(\Delta_{1b,1}\vert\Delta_{1b,1})$
	$1c(3m1')$	$+\mathrm{mod}_3(\Delta_{1c,1}\vert\Delta_{1c,1})$
$p31m$	$1a(3m)$	$\mathrm{mod}_3(\Delta_{1a,1}\vert\Delta_{1a,1})$
	$2b(3)$	$+2\mathrm{mod}_3(\Delta_{2b,1}\vert\Delta_{2b,2})$
$p31m1'$	$1a(3m1')$	$\mathrm{mod}_3(\Delta_{1a,1}\vert\Delta_{1a,1})$
	$2b(31')$	$+2\mathrm{mod}_3(\Delta_{2b,1}\vert\Delta_{2b,1})$
$p6$	$1a(6)$	$\mathrm{mod}_6(\Delta_{1a,1}-2\Delta_{1a,2}\vert\Delta_{1a,1})$
	$2b(3)$	$+2\mathrm{mod}_3(\Delta_{2b,1}\vert\Delta_{2b,2})$
	$3c(2)$	$+3\mathrm{mod}_2(\Delta_{3c,1}\vert\Delta_{3c,1})$
$p61'$	$1a(61')$	$\mathrm{mod}_6(\Delta_{1a,1}\vert\Delta_{1a,1})$
	$2b(31')$	$+2\mathrm{mod}_3(\Delta_{2b,1}\vert\Delta_{2b,1})$
	$3c(2)$	$+3\mathrm{mod}_2(\Delta_{3c,1}\vert\Delta_{3c,1})$
$p6mm$	$1a(6mm)$	$\mathrm{mod}_6(\Delta_{1a,1}\vert\Delta_{1a,1})$
	$2b(3m)$	$+2\mathrm{mod}_3(\Delta_{2b,1}\vert\Delta_{2b,1})$
	$3c(2mm)$	$+3\mathrm{mod}_2(\Delta_{3c,1}\vert\Delta_{3c,1})$
$p6mm1'$	$1a(6mm1')$	$\mathrm{mod}_6(\Delta_{1a,1}\vert\Delta_{1a,1})$
	$2b(3m1')$	$+2\mathrm{mod}_3(\Delta_{2b,1}\vert\Delta_{2b,1})$
	$3c(2mm1')$	$+3\mathrm{mod}_2(\Delta_{3c,1}\vert\Delta_{3c,1})$

TABLE IX: Supplementary Table 9. Fragile Criteria (with SOC)

SG	WP	$N_{occ} < \dots$
$p1$	$a(1)$	
$p1'$	$a(1')$	
$p2$	$1a(2)$	$\mathrm{mod}_2(\Delta_{1a,1}\vert\Delta_{1a,1})$
	$1b(2)$	$+\mathrm{mod}_2(\Delta_{1b,1}\vert\Delta_{1b,1})$
	$1c(2)$	$+\mathrm{mod}_2(\Delta_{1c,1}\vert\Delta_{1c,1})$
	$1d(2)$	$+\mathrm{mod}_2(\Delta_{1d,1}\vert\Delta_{1d,1})$
$p21'$	$1a(21')$	$2\mathrm{mod}_2(\Delta_{1a,2}\vert\Delta_{1a,1})$
	$1b(21')$	$+2\mathrm{mod}_2(\Delta_{1b,2}\vert\Delta_{1b,1})$
	$1c(21')$	$+2\mathrm{mod}_2(\Delta_{1c,2}\vert\Delta_{1c,1})$
	$1d(21')$	$+2\mathrm{mod}_2(\Delta_{1d,2}\vert\Delta_{1d,1})$
pm	$1a(m)$	
	$1b(m)$	
$pm1'$	$1a(m1')$	
	$1b(m1')$	
pg		
$pg1'$		
cm	$2a(m)$	
$cm1'$	$2a(m1')$	
$p2mm$	$1a(2mm)$	$\mathrm{mod}_2(0\vert\Delta_{1a,1})$
	$1b(2mm)$	$+\mathrm{mod}_2(0\vert\Delta_{1b,1})$
	$1c(2mm)$	$+\mathrm{mod}_2(0\vert\Delta_{1c,1})$
	$1d(2mm)$	$+\mathrm{mod}_2(0\vert\Delta_{1d,1})$
$p2mm1'$	$1a(2mm1')$	$2\mathrm{mod}_2(\Delta_{1a,2}\vert\Delta_{1a,1})$
	$1b(2mm1')$	$+2\mathrm{mod}_2(\Delta_{1b,2}\vert\Delta_{1b,1})$
	$1c(2mm1')$	$+2\mathrm{mod}_2(\Delta_{1c,2}\vert\Delta_{1c,1})$
	$1d(2mm1')$	$+2\mathrm{mod}_2(\Delta_{1d,2}\vert\Delta_{1d,1})$
$p2mg$	$2a(2)$	$2\mathrm{mod}_2(\Delta_{2a,1}\vert\Delta_{2a,1})$
	$2b(2)$	$+2\mathrm{mod}_2(\Delta_{2b,1}\vert\Delta_{2b,1})$
	$4c(m)$	
$p2mg1'$	$2a(21')$	$4\mathrm{mod}_2(\Delta_{2a,2}\vert\Delta_{2a,1})$
	$2b(21')$	$+4\mathrm{mod}_2(\Delta_{2b,2}\vert\Delta_{2b,1})$
	$4c(m1')$	
$p2gg$	$2a(2)$	$2\mathrm{mod}_2(\Delta_{2a,1}\vert\Delta_{2a,1})$
	$2b(2)$	$+2\mathrm{mod}_2(\Delta_{2b,1}\vert\Delta_{2b,1})$
$p2gg1'$	$2a(21')$	$4\mathrm{mod}_2(\Delta_{2a,2}\vert\Delta_{2a,1})$
	$2b(21')$	$+4\mathrm{mod}_2(\Delta_{2b,2}\vert\Delta_{2b,1})$
$c2mm$	$2a(2mm)$	$2\mathrm{mod}_2(0\vert\Delta_{2a,1})$
	$2b(2mm)$	$+2\mathrm{mod}_2(0\vert\Delta_{2b,1})$
	$4c(2)$	$+4\mathrm{mod}_2(\Delta_{4c,1}\vert\Delta_{4c,1})$
$c2mm1'$	$2a(2mm1')$	$4\mathrm{mod}_2(\Delta_{2a,2}\vert\Delta_{2a,1})$
	$2b(2mm1')$	$+4\mathrm{mod}_2(\Delta_{2b,2}\vert\Delta_{2b,1})$
	$4c(21')$	$+8\mathrm{mod}_2(\Delta_{4c,2}\vert\Delta_{4c,1})$
$p4$	$1a(4)$	$\mathrm{mod}_4(2\Delta_{1a,2}-\Delta_{1a,1}\vert\Delta_{1a,1})$
	$1b(4)$	$+\mathrm{mod}_4(2\Delta_{1b,2}-\Delta_{1b,1}\vert\Delta_{1b,1})$
	$2c(2)$	$+2\mathrm{mod}_2(\Delta_{2c,1}\vert\Delta_{2c,1})$
$p41'$	$1a(41')$	$2\mathrm{mod}_4(2\Delta_{1a,2}\vert\Delta_{1a,1})$
	$1b(41')$	$+2\mathrm{mod}_4(2\Delta_{1b,2}\vert\Delta_{1b,1})$
	$2c(21')$	$+4\mathrm{mod}_4(2\Delta_{2c,2}\vert\Delta_{2c,1})$

$p4mm$	$1a(4mm)$	$\text{mod}_4(2\Delta_{1a,2}\vert\Delta_{1a,1})$
	$1b(4mm)$	$\text{mod}_4(2\Delta_{1b,2}\vert\Delta_{1b,1})$
	$2c(2mm)$	$2\text{mod}_4(0\vert\Delta_{2c,1})$
$p4mm1'$	$1a(4mm1')$	$\text{mod}_4(\Delta_{1a,2}\vert\Delta_{1a,1})$
	$1b(4mm1')$	$+\text{mod}_4(\Delta_{1b,2}\vert\Delta_{1b,1})$
	$2c(2mm1')$	$+2\text{mod}_4(\Delta_{2c,2}\vert\Delta_{2c,1})$
$p4gm$	$2a(4)$	$2\text{mod}_4(2\Delta_{2a,2}-\Delta_{2a,2}\vert\Delta_{2a,2})$
	$2b(2mm)$	$2\text{mod}_4(0\vert\Delta_{2b,2})$
$p4gm1'$	$2a(41')$	$4\text{mod}_4(\Delta_{2a,2}\vert\Delta_{2a,1})$
	$2b(2mm1')$	$+4\text{mod}_4(\Delta_{2b,2}\vert\Delta_{2b,1})$
$p3$	$1a(3)$	$\text{mod}_3(\Delta_{1a,1}\vert\Delta_{1a,2})$
	$1b(3)$	$+\text{mod}_3(\Delta_{1b,1}\vert\Delta_{1b,2})$
	$1c(3)$	$+\text{mod}_3(\Delta_{1c,1}\vert\Delta_{1c,2})$
$p31'$	$1a(31')$	$2\text{mod}_3(\Delta_{1a,1}\vert\Delta_{1a,1})$
	$1b(31')$	$+2\text{mod}_3(\Delta_{1b,1}\vert\Delta_{1b,1})$
	$1c(31')$	$+2\text{mod}_3(\Delta_{1c,1}\vert\Delta_{1c,1})$
$p3m1$	$1a(3m)$	$\text{mod}_3(\Delta_{1a,1}\vert\Delta_{1a,1})$
	$1b(3m)$	$+\text{mod}_3(\Delta_{1b,1}\vert\Delta_{1b,1})$
	$1c(3m)$	$+\text{mod}_3(\Delta_{1c,1}\vert\Delta_{1c,1})$
$p3m1'$	$1a(3m1')$	$2\text{mod}_3(\Delta_{1a,1}\vert\Delta_{1a,1})$
	$1b(3m1')$	$+2\text{mod}_3(\Delta_{1b,1}\vert\Delta_{1b,1})$
	$1c(3m1')$	$+2\text{mod}_3(\Delta_{1c,1}\vert\Delta_{1c,1})$
$p31m$	$1a(3m)$	$\text{mod}_3(\Delta_{1a,1}\vert\Delta_{1a,1})$
	$2b(3)$	$+2\text{mod}_3(\Delta_{2b,1}\vert\Delta_{2b,2})$
$p31m1'$	$1a(3m1')$	$2\text{mod}_3(\Delta_{1a,1}\vert\Delta_{1a,1})$
	$2b(31')$	$+4\text{mod}_3(\Delta_{2b,1}\vert\Delta_{2b,1})$
$p6$	$1a(6)$	$\text{mod}_6(2\Delta_{1a,2}-\Delta_{1a,1}\vert\Delta_{1a,1})$
	$2b(3)$	$+2\text{mod}_3(\Delta_{2b,1}+\Delta_{2b,2})$
	$3c(2)$	$+3\text{mod}_2(\Delta_{3c,1})$
$p61'$	$1a(61')$	$2\text{mod}_6(\Delta_{1a,2}\vert\Delta_{1a,1})$
	$2b(31')$	$+4\text{mod}_3(2\Delta_{2b,1}\vert\Delta_{2b,1})$
	$3c(2)$	$+6\text{mod}_2(\Delta_{3c,2}\vert\Delta_{3c,1})$
$p6mm$	$1a(6mm)$	$\text{mod}_6(2\Delta_{1a,2}\vert\Delta_{1a,1})$
	$2b(3m)$	$+2\text{mod}_3(\Delta_{2b,1}\vert\Delta_{2b,1})$
	$3c(2mm)$	$+3\text{mod}_2(0\vert\Delta_{3c,1})$
$p6mm1'$	$1a(6mm1')$	$2\text{mod}_6(\Delta_{1a,2}\vert\Delta_{1a,1})$
	$2b(3m1')$	$+4\text{mod}_3(\Delta_{2b,1}\vert\Delta_{2b,1})$
	$3c(2mm1')$	$+6\text{mod}_6(\Delta_{3c,2}\vert\Delta_{3c,1})$

IV. SUPPLEMENTARY NOTE 4

A. Stable Topology and Global RSIs

In this Supplementary Note, we study many-body stable topology using global many-body RSIs, which are defined using the same operators in Supplementary Note II C but evaluated on periodic boundary conditions. Supplementary Note IV B proves that the global many-body RSIs defined in this way vanish in all many-body atomic and many-body fragile topological states. Supplementary Note IV C then evaluates the global many-body RSIs on general product states, finding that they can be nonzero in symmetry-indicated Chern insulators.

B. Global Many-body RSIs in Many-body Atomic and Fragile States

In this section, we show that global many-body RSIs, defined in the Main Text, vanish in all many-body atomic states. Since the RSIs of many-body fragile states are the differences of the RSIs of many-body atomic states, this result shows that the global RSIs vanish there as all. We can state both results succinctly as: global many-body RSIs vanish if the many-body local RSIs are well-defined. We first prove our result in the spinless rotation groups, and then we discuss the addition of mirrors, time-reveral, and SOC. In the following, we fix a specific Wyckoff position $\mathbf{x}$ with point group $G_{\mathbf{x}}$. We then define global many-body RSIs at that Wyckoff position. In a given wallpaper group, there are global many-body RSIs defined at each Wyckoff position in the unit cell. For instance in $p2$, the global many-body RSI $\Delta^G_{1a,1}$ at $\mathbf{x}$ =1a with $G_{1a} = \{1, C_2\}$ is computed from $e^{i\frac{\pi}{2}\hat{N}}C_2 \left|GS, PBC\right\rangle = e^{i\frac{\pi}{2}\Delta^G_{1a,1}} \left|GS, PBC\right\rangle$, whereas the global many-body RSI at $\mathbf{x}$ =1b with $G_{1b} = \{1, T_1C_2\}$ is computed from $e^{i\frac{\pi}{2}\hat{N}}T_1C_2 \left|GS, PBC\right\rangle = e^{i\frac{\pi}{2}\Delta^G_{1b,1}} \left|GS, PBC\right\rangle$. We see that $\Delta^G_{1a,1}$ and $\Delta^G_{1b,1}$ are related by the total many-body momentum of the state. In the following, we consider a fixed Wyckoff position with C_n understood to be the rotation centered at that Wyckoff position. For convenience, we choose the origin so that $\mathbf{x} = (0, 0)$, so the formulae we prove are for the 1a many-body global RSIs $\Delta^G_{1a,i}$, but the many-body global RSIs at other Wyckoff positions can be obtained using the total many-body momentum from translations.

The crucial feature of calculating the many-body global RSIs on periodic boundary conditions (PBCs) is that multiple points in the large spatial torus are invariant under the same rotation symmetry, unlike for OBCs where only a single point (the rotation center) is invariant. All other effective rotation symmetries like T_1C_n can be obtained from C_n with translation operators, or equivalently by shifting the origin. Thus it is enough to consider only C_n. For

each rotation C_n, we denote the set of points

$$\mathbf{x}^G = \{\mathbf{x}|C_n^m\mathbf{x} = \mathbf{x} \mod L_1\mathbf{a}_1, L_2\mathbf{a}_2 \; \forall \; 1 \leq m < n\} \; . \tag{69}$$

Supplementary Figure 6 depicts the various cases with $\mathbf{x}^G$ shown by colored dots. Mathematically, for periodic boundary conditions on an $L_1\mathbf{a}_1 \times L_2\mathbf{a}_2$ lattice, a C_n-invariant point $\mathbf{r}$ is defined by $C_n\mathbf{r} = \mathbf{r} \mod L_1\mathbf{a}_1, L_2\mathbf{a}_2$. We also require that L_1, L_2 are defined such that the high-symmetry points in the Brillouin zone exist, e.g. L_1, L_2 must be even with C_2 symmetry and multiples of three with C_3 symmetry.

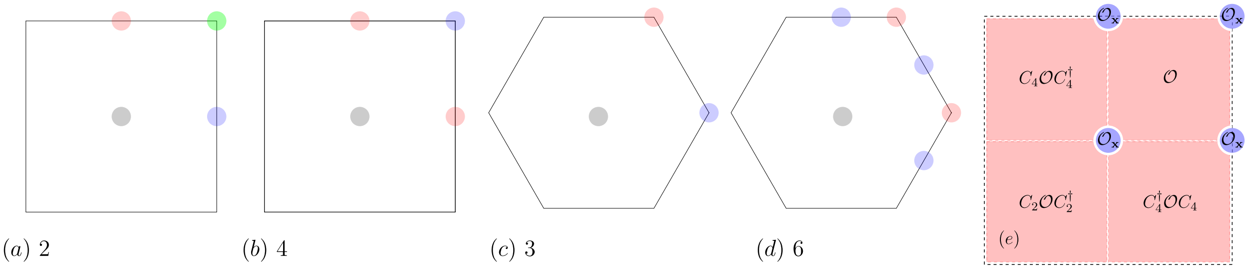

(a) 2 (b) 4 (c) 3 (d) 6

FIG. 6. Supplementary Figure 6. We show the Wyckoff positions $\mathbf{x}^G$ that contribute to the global RSI at the 1a position in the PGs $2, 3, 4, 6$. The plots here show the entire sample on periodic boundary conditions, and we assume the center of the C_n operator is at the 1a position (center of the sample). Adding mirrors and TRS simply reduces the RSI expressions. Points of the same color are mapped to each other by the elementary rotation of the group. (a) There are four points symmetric under C_2. (b) There are two points (gray, blue) symmetric under C_4, and the red points are exchanged under C_4. (c) There are three points symmetric under C_3. (d) There is one point (gray) symmetric under C_6, two points (red) that are exchanged under C_6, and three points (blue) that are permuted under C_6. (e) We depict the partitioning of a many-body atomic limit state on a spatial torus (PBCs) in rotation-related operators $\mathcal{O}$ and the finite $\mathcal{O}_\mathbf{x}$ operators at fixed points of the rotation $\mathbf{x} \in \mathbf{x}^G$.

Following identical steps as in Supplementary Note II C, we consider many-body atomic limit states on periodic boundary conditions. In the notation of Supplementary Note II C, they can be written as

$$|GS, PBC\rangle = \prod_{\mathbf{R},\mathbf{r}} \mathcal{O}_{\mathbf{R},\mathbf{r}} |0\rangle = \prod_{\mathbf{x}\in\mathbf{x}^G} \mathcal{O}_\mathbf{x} \prod_{i=1}^{n} C_n^i \mathcal{O} C_n^{i\dagger} \; |0\rangle \tag{70}$$

where $\mathcal{O} = \prod_{\mathbf{R}+\mathbf{r}\in\mathcal{D}} \mathcal{O}_{\mathbf{R},\mathbf{r}}$ (see Supplementary Note II C) and $\mathcal{D}$ is $1/n$th of the total space excluding $\mathbf{x}^G$, and is depicted as one of the shaded red squares in Supplementary Figure 6(e) for $C_n = C_4$. Supplementary Note II C showed explicitly that the RSI operators $Q_i = e^{i\frac{\pi}{n}\hat{N}} C_n, C_n^2$ for n even and $Q_i = C_n, e^{i\frac{2\pi}{n}\hat{N}}$ for n odd commute with $\prod_{i=1}^n C_n^i \mathcal{O} C_n^{i\dagger}$ as shown in Eq. 22. In the context of Supplementary Note II C, the factor $\prod_{i=1}^n C_n^i \mathcal{O} C_n^{i\dagger}$ appeared when increasing the size of the cutoff, under which the RSI operators are invariant. Analogously on PBCs, increasing L_1, L_2 increases the number of particle in $\prod_{i=1}^n C_n^i \mathcal{O} C_n^{i\dagger}$, but crucially does not change the number of sites in $\mathbf{x}^G$. Because the RSI operators commute with $\prod_{i=1}^n C_n^i \mathcal{O} C_n^{i\dagger}$, we have

$$\begin{aligned} Q_i |GS, PBC\rangle &= Q_i \prod_{\mathbf{x}\in\mathbf{x}^G} \mathcal{O}_\mathbf{x} \; Q_i^\dagger Q_i \prod_{i=1}^{n} C_n^i \mathcal{O} C_n^{i\dagger} Q_i^\dagger |0\rangle \\ &= \left(Q_i \prod_{\mathbf{x}\in\mathbf{x}^G} \mathcal{O}_\mathbf{x} \; Q_i^\dagger \right) \prod_{i=1}^{n} C_n^i \mathcal{O} C_n^{i\dagger} |0\rangle \; . \end{aligned} \tag{71}$$

Thus we only need to compute the object in parentheses for each rotation group. We can perform the computation systematically using the translation operators which yield $\mathcal{O}_\mathbf{x} = T_\mathbf{x} \mathcal{O}_{(0,0)} T_\mathbf{x}^\dagger$ since $\mathbf{x} \in \mathbf{x}^G$ are lattice vectors. Then we have

$$C_n \mathcal{O}_\mathbf{x} C_n^\dagger = C_n T_\mathbf{x} \mathcal{O}_{(0,0)} T_\mathbf{x}^\dagger C_n = T_{C_n\mathbf{x}} C_n \mathcal{O}_{(0,0)} C_n^\dagger T_{C_n\mathbf{x}}^\dagger = e^{i\lambda[C_n]} \mathcal{O}_{C_n\mathbf{x}} \tag{72}$$

where $\lambda[C_n] \in \frac{2\pi}{n}\mathbb{Z}_n$ is the angular momentum of $\mathcal{O}_{(0,0)}$. It follows that

$$\begin{aligned} e^{i\frac{\pi}{n}\hat{N}} \prod_{\mathbf{x}\in\mathbf{x}^G} \mathcal{O}_\mathbf{x} \; e^{-i\frac{\pi}{n}\hat{N}} &= e^{i\frac{\pi}{n} n_G N_\mathcal{O}} \prod_{\mathbf{x}\in\mathbf{x}^G} \mathcal{O}_\mathbf{x} \\ C_n \prod_{\mathbf{x}\in\mathbf{x}^G} \mathcal{O}_\mathbf{x} \; C_n^\dagger &= e^{i n_G \lambda[C_n]} \prod_{\mathbf{x}\in\mathbf{x}^G} \mathcal{O}_{C_n\mathbf{x}} = e^{i n_G \lambda[C_n]} \left(\det{}_{(-1)^{N_\mathcal{O}}} R[C_n] \right) \prod_{\mathbf{x}\in\mathbf{x}^G} \mathcal{O}_\mathbf{x} \end{aligned} \tag{73}$$

where n_G is the number of sites in $\mathbf{x}^G$ (the multiplicity of $\mathbf{x}^G$ on PBCs), $N_{\mathcal{O}}$ is the total charge of $\mathcal{O}_{(0,0)}$, and $R[C_n]$ is the permutation matrix defined by $R_{ij}[C_n] = 1$ if $C_n \mathbf{x}_i = \mathbf{x}_j$ for $\mathbf{x}^G = \mathbf{x}_1, \dots, \mathbf{x}_{n^G}$ (see Eq. 33). From Supplementary Figure 6 which marks points in the same color if they are permuted to each other under C_n, we find

$$R[C_2] = \begin{pmatrix} 1 & & & \\ & 1 & & \\ & & 1 & \\ & & & 1 \end{pmatrix}, \quad R[C_3] = \begin{pmatrix} 1 & & \\ & 1 & \\ & & 1 \end{pmatrix}, \quad R[C_4] = \begin{pmatrix} 1 & & & \\ & 1 & & \\ & & 0 & 1 \\ & & 1 & 0 \end{pmatrix}, \quad R[C_6] = \begin{pmatrix} 1 & & & & & \\ & 0 & 1 & & & \\ & 1 & 0 & & & \\ & & & 0 & 1 & \\ & & & & 0 & 1 \\ & & & 1 & & 0 \end{pmatrix} \tag{74}$$

for which it is easy to check that $\det{}_{\pm} R[C_2] = \det{}_{\pm} R[C_3] = +1$ and $\det{}_{\pm} R[C_4] = \det{}_{\pm} R[C_6] = \pm 1$. We now compute the global RSIs Δ_1^G using Eq. 73:

$$\begin{aligned}
C_2: \quad & e^{i\frac{\pi}{2}\Delta_1^G} \prod_{\mathbf{x}\in\mathbf{x}^G} \mathcal{O}_{\mathbf{x}} = e^{i\frac{\pi}{2}\hat{N}} C_2 \prod_{\mathbf{x}\in\mathbf{x}^G} \mathcal{O}_{\mathbf{x}} \, (e^{i\frac{\pi}{2}\hat{N}} C_2)^\dagger = e^{i\frac{\pi}{2}4N_{\mathcal{O}} + i4\lambda[C_2]} \prod_{\mathbf{x}\in\mathbf{x}^G} \mathcal{O}_{\mathbf{x}} = + \prod_{\mathbf{x}\in\mathbf{x}^G} \mathcal{O}_{\mathbf{x}} \\
C_3: \quad & e^{i\frac{2\pi}{3}\Delta_1^G} \prod_{\mathbf{x}\in\mathbf{x}^G} \mathcal{O}_{\mathbf{x}} = C_3 \prod_{\mathbf{x}\in\mathbf{x}^G} \mathcal{O}_{\mathbf{x}} \, C_3^\dagger = e^{i3\lambda[C_3]} \prod_{\mathbf{x}\in\mathbf{x}^G} \mathcal{O}_{\mathbf{x}} = + \prod_{\mathbf{x}\in\mathbf{x}^G} \mathcal{O}_{\mathbf{x}} \\
C_4: \quad & e^{i\frac{\pi}{4}\Delta_1^G} \prod_{\mathbf{x}\in\mathbf{x}^G} \mathcal{O}_{\mathbf{x}} = e^{i\frac{\pi}{4}\hat{N}} C_4 \prod_{\mathbf{x}\in\mathbf{x}^G} \mathcal{O}_{\mathbf{x}} \, (e^{i\frac{\pi}{4}\hat{N}} C_4)^\dagger = e^{i\frac{\pi}{4}4N_{\mathcal{O}} + i4\lambda[C_4]} (-1)^{N_{\mathcal{O}}} \prod_{\mathbf{x}\in\mathbf{x}^G} \mathcal{O}_{\mathbf{x}} = + \prod_{\mathbf{x}\in\mathbf{x}^G} \mathcal{O}_{\mathbf{x}} \\
C_6: \quad & e^{i\frac{\pi}{6}\Delta_1^G} \prod_{\mathbf{x}\in\mathbf{x}^G} \mathcal{O}_{\mathbf{x}} = e^{i\frac{\pi}{6}\hat{N}} C_6 \prod_{\mathbf{x}\in\mathbf{x}^G} \mathcal{O}_{\mathbf{x}} \, (e^{i\frac{\pi}{6}\hat{N}} C_6)^\dagger = e^{i\frac{\pi}{6}6N_{\mathcal{O}} + i6\lambda[C_6]} (-1)^{N_{\mathcal{O}}} \prod_{\mathbf{x}\in\mathbf{x}^G} \mathcal{O}_{\mathbf{x}} = + \prod_{\mathbf{x}\in\mathbf{x}^G} \mathcal{O}_{\mathbf{x}}
\end{aligned} \tag{75}$$

showing that $\Delta_1^G = 0$ in all cases, using $n\lambda[C_n] = 0 \mod 2\pi$. Next we show that the Δ_2^G global RSIs also vanish:

$$\begin{aligned}
C_3: \quad & e^{i\frac{2\pi}{3}\Delta_2^G} \prod_{\mathbf{x}\in\mathbf{x}^G} \mathcal{O}_{\mathbf{x}} = e^{i\frac{2\pi}{3}\hat{N}} \prod_{\mathbf{x}\in\mathbf{x}^G} \mathcal{O}_{\mathbf{x}} \, e^{-i\frac{2\pi}{3}\hat{N}} = e^{i\frac{2\pi}{3}3N_{\mathcal{O}}} \prod_{\mathbf{x}\in\mathbf{x}^G} \mathcal{O}_{\mathbf{x}} = + \prod_{\mathbf{x}\in\mathbf{x}^G} \mathcal{O}_{\mathbf{x}} \\
C_4: \quad & e^{i\pi\Delta_2^G} \prod_{\mathbf{x}\in\mathbf{x}^G} \mathcal{O}_{\mathbf{x}} = C_4^2 \prod_{\mathbf{x}\in\mathbf{x}^G} \mathcal{O}_{\mathbf{x}} C_4^{2\dagger} = (e^{i4\lambda[C_4]} (-1)^{N_{\mathcal{O}}})^2 \prod_{\mathbf{x}\in\mathbf{x}^G} \mathcal{O}_{\mathbf{x}} = + \prod_{\mathbf{x}\in\mathbf{x}^G} \mathcal{O}_{\mathbf{x}} \\
C_6: \quad & e^{i\frac{2\pi}{3}\Delta_2^G} \prod_{\mathbf{x}\in\mathbf{x}^G} \mathcal{O}_{\mathbf{x}} = C_6^2 \prod_{\mathbf{x}\in\mathbf{x}^G} \mathcal{O}_{\mathbf{x}} \, C_6^{2\dagger} = (e^{i6\lambda[C_6]} (-1)^{N_{\mathcal{O}}})^2 \prod_{\mathbf{x}\in\mathbf{x}^G} \mathcal{O}_{\mathbf{x}} = + \prod_{\mathbf{x}\in\mathbf{x}^G} \mathcal{O}_{\mathbf{x}}
\end{aligned} \tag{76}$$

showing that $\Delta_2^G = 0$ in all cases. Note that the C_2 case is trivial because there is no Δ_2^G invariant.

Thus in all spinless rotation groups with PBCs preserving the high-symmetry points, we have shown that the global many-body RSI vanish in many-body atomic limits. In the spinless groups, this immediately extends to all the points groups adding mirrors and/or time-reversal because the RSIs in these cases can be obtained from the rotation subgroups by reduction (see Supplementary Note II D). Similarly, the rotation groups with SOC are isomorphic to the rotation groups without SOC and thus all the global many-body RSIs vanish there as well. With SOC, adding mirrors simply reduces the RSIs of the rotation groups, but we must consider the SOC groups with time-reversal separately. In these groups, there is always a many-body local RSI $\Delta_2 = N/2 \mod n$ which is not obtained by reduction. Δ_2 is the eigenvalue of $e^{i\frac{2\pi}{n}\frac{\hat{N}}{2}}$, which intuitively counts the number of Kramers pairs (since $\mathcal{T}^2 = -1$) mod n. However, the global many-body RSI $e^{i\frac{2\pi}{n}\frac{\hat{N}}{2}} |GS, PBC\rangle = e^{i\frac{2\pi}{n}\Delta_2^G} |GS, PBC\rangle$ is always trivial as we now show. This follows because $\mathcal{O}_{\mathbf{x}}$ must always create an even number of electrons since the state is non-degenerate, and $\mathbf{x}^G$ always contains a multiple of n points with C_n symmetry. Thus $e^{i\frac{2\pi}{n}\frac{\hat{N}}{2}} |GS, PBC\rangle = +|GS, PBC\rangle$.

C. Many-Body Global RSIs in the Band Theory Limit

We now show that nontrivial many-body global RSIs can be obtained from non-interacting Chern insulators. A similar calculation has been formed in **??**, but to be self-contained we give the full details in our formalism here. First we define the momentum operators

$$c^\dagger_{\mathbf{k},\alpha} = \frac{1}{\sqrt{L_1 L_2}} \sum_{\mathbf{R}} e^{-i\mathbf{k}\cdot(\mathbf{R}+\mathbf{r}_\alpha)} c^\dagger_{\mathbf{R},\alpha}, \qquad \mathbf{k} = k_1 \mathbf{b}_1 + k_2 \mathbf{b}_2 \tag{77}$$

where $\mathbf{b}_i \cdot \mathbf{a}_j = \delta_{ij}$, $\alpha = 1, \ldots, N_{orb}$ is the orbital index, $\mathbf{r}_\alpha$ is the location of the α orbital, and the sum is over lattice vectors $\mathbf{R} = m\mathbf{a}_1 + n\mathbf{a}_2, m = 0, \ldots, L_1 - 1, n = 0, \ldots, L_2 - 1$. The momenta (defined mod 2π) are

$$k_1 = 0, \frac{2\pi}{L_1}, \ldots, \frac{2\pi}{L_1}(L_1 - 1), \quad k_2 = 0, \frac{2\pi}{L_2}, \ldots, \frac{2\pi}{L_2}(L_2 - 1). \tag{78}$$

In general, we require that $L_1, L_2 \in \mathbb{N}$ are chosen such that all high symmetry momenta appear in Eq. 78, e.g. L_1, L_2 are even in systems with a C_2 symmetry. The Bloch states transform as eigenstates under translation via

$$T_i c^\dagger_{\mathbf{k},\alpha} T_i^\dagger = e^{i\mathbf{k}\cdot\mathbf{a}_i} c^\dagger_{\mathbf{k},\alpha} \tag{79}$$

and under the (symmorphic) PG symmetries, e.g. rotations, g as

$$g c^\dagger_{\mathbf{k},\alpha} g^\dagger = \sum_\beta c^\dagger_{g\mathbf{k},\beta} D_{\beta\alpha}[g] \tag{80}$$

where $D[g]$ is the $N_{orb} \times N_{orb}$ representation matrix of g on the orbitals. For spinless/spinfull electrons, $D[C_n^n] = D[M^2] = D[\mathcal{T}^2] = \pm 1$.

In non-interacting Hamiltonian, the eigenstate creation operators are $\gamma^\dagger_{\mathbf{k},n} = \sum_\alpha c^\dagger_{\mathbf{k},\alpha} U_{\alpha n}(\mathbf{k})$ where $U_{\alpha n}(\mathbf{k})$ are the elements of an $N_{orb} \times N_{orb}$ unitary matrix which rotates between the orbital index α and the band index n. The energies determined by the Hamiltonian are $[H, \gamma^\dagger_{\mathbf{k},n}] = E_n(\mathbf{k})\gamma^\dagger_{\mathbf{k},n}$. In an insulator, there is a gap between the N_{occ} occupied bands and the $N_{orb} - N_{occ}$ unoccupied bands. We define the rectangular $N_{orb} \times N_{occ}$ matrix $[U(\mathbf{k})]_{\alpha n}$, $\alpha = 1, \ldots, N_{orb}, n = 1, \ldots, N_{occ}$ to be the eigenvectors of the occupied bands. It obeys $U^\dagger(\mathbf{k})U(\mathbf{k}) = \mathbb{1}_{N_{occ}\times N_{occ}}$ and $U(\mathbf{k})U^\dagger(\mathbf{k}) = P(\mathbf{k})$ where $P(\mathbf{k})^2 = P(\mathbf{k})$ is the rank N_{occ} projector[8]. Symmetry enforces $E_n(g\mathbf{k}) = E_n(\mathbf{k})$ and

$$U(g\mathbf{k}) = D[g]U(\mathbf{k})B_g^\dagger(\mathbf{k}), \qquad B_g(\mathbf{k}) = U^\dagger(g\mathbf{k})D[g]U(\mathbf{k}) \tag{81}$$

where $B_g(\mathbf{k})$ is called the sewing matrix[9] and is an $N_{occ} \times N_{occ}$ unitary matrix with nonzero elements only between states of the same energy. At high symmetry points $\mathbf{K} = g\mathbf{K} \mod 2\pi\mathbf{b}_i$ for $g \in G_\mathbf{K}$, $B_g(\mathbf{K})$ is the representation matrix of the little group $G_\mathbf{K}$[8]. A simple calculation gives

$$\begin{aligned} g\gamma^\dagger_{\mathbf{k},n} g^\dagger &= \sum_\alpha g c^\dagger_{\mathbf{k},\alpha} g^\dagger U_{\alpha n}(\mathbf{k}) \\ &= \sum_\beta c^\dagger_{g\mathbf{k},\beta} [D[g]U(\mathbf{k})]_{\beta n} \\ &= \sum_\beta c^\dagger_{g\mathbf{k},\beta} [D[g]U(\mathbf{k})B_g^\dagger(\mathbf{k})B_g(\mathbf{k})]_{\beta n} \\ &= \sum_\beta c^\dagger_{g\mathbf{k},\beta} [U(g\mathbf{k})B_g(\mathbf{k})]_{\beta n} \\ &= \sum_m \gamma^\dagger_{g\mathbf{k},m} [B_g(\mathbf{k})]_{mn} \end{aligned} \tag{82}$$

using Eq. 81. With this result, we can evaluate the action of a symmetry operators on any state. However, it will be useful to express Eq. 82 in a more general form:

$$g\gamma^\dagger_{\mathbf{k},n} g^\dagger = \sum_{\mathbf{k}'m} \gamma^\dagger_{\mathbf{k}',m} [\mathcal{B}_g]_{\mathbf{k}'m,\mathbf{k}n}, \qquad [\mathcal{B}_g]_{\mathbf{k}'m,\mathbf{k}n} = \delta_{\mathbf{k}',g\mathbf{k}} [B_g(\mathbf{k})]_{mn} \tag{83}$$

such that $\mathcal{B}_{\mathbf{k}'m,\mathbf{k}n}[g]$ is the $L_1L_2N_{occ} \times L_1L_2N_{occ}$ representation matrix of g on *all* the occupied states (it is a space group representation).

We now consider the groundstate of a band insulator denoted

$$|GS\rangle = \prod_{\mathbf{k},n} \gamma^\dagger_{\mathbf{k},n} \, |0\rangle \tag{84}$$

where the product is taken over the $L_1L_2N_{occ}$ occupied states in the BZ. Eq. 83 now immediately gives

$$g\,|GS\rangle = \det \mathcal{B}_g \; |GS\rangle \tag{85}$$

because there are $L_1L_2N_{occ}$ anti-commuting $\gamma^\dagger_{\mathbf{k},n}$ operators which fully anti-symmetrize the $L_1L_2N_{occ} \times L_1L_2N_{occ}$ matrix $\mathcal{B}_g$. It is easy to evaluate $\det \mathcal{B}_g$ since $\mathcal{B}_g$ is block-diagonalized into representations, and the determinant of a direct sum is the product of their determinants. We now calculate the representations at all points in the BZ, which divide into high-symmetry points with nontrivial little groups (of which there are only a finite number in each space group) and non-high-symmetry points where the little group is trivial (of which there is a macroscopic number). We will see that the high-symmetry point irrep determine the Chern number mod n and the non-high-symmetry points contribute overall phases which are canceled due to our formula for the global RSI.

Let us study $G = p2$ with C_2 and translations. All states off the high-symmetry momenta with $\mathbf{k} \neq -\mathbf{k} \mod BZ$ transform in a 2D representation of C_2 (if there are accidental degeneracies, we allow for a sum of 2D representations), since C_2 exchanges the distinct states $\mathbf{k}, -\mathbf{k}$ with an phase factor $e^{i\theta}$ determined by the eigenvectors which cancels in the determinant:

$$C_2 \begin{pmatrix} \gamma^\dagger_{\mathbf{k},n} \\ \gamma^\dagger_{-\mathbf{k},n} \end{pmatrix} C_2^\dagger = \begin{pmatrix} e^{i\theta}\gamma^\dagger_{-\mathbf{k},n} \\ e^{-i\theta}\gamma^\dagger_{\mathbf{k},n} \end{pmatrix} = \begin{pmatrix} 0 & e^{i\theta} \\ e^{-i\theta} & 0 \end{pmatrix} \begin{pmatrix} \gamma^\dagger_{\mathbf{k},n} \\ \gamma^\dagger_{-\mathbf{k},n} \end{pmatrix}, \qquad \det \begin{pmatrix} 0 & e^{i\theta} \\ e^{-i\theta} & 0 \end{pmatrix} = -1 \ . \tag{86}$$

From the group theory perspective, states off high-symmetry momenta have a trivial little group, and their representation can be expressed as $E \uparrow G = A \oplus B$ where E is the irrep of the trivial group. Note that $\det A \oplus B = (+1)(-1) = -1$ in agreement with Eq. 86. Thus we have shown that all states off the high-symmetry momenta contribute a factor of (-1) per pair. The only remaining states at the four high-symmetry momenta given by

$$\Gamma = (0,0), \quad X = \pi \mathbf{b}_1, \quad Y = \pi \mathbf{b}_2, \quad M = \pi \mathbf{b}_1 + \pi \mathbf{b}_2 \tag{87}$$

whose irreps are denoted $\Gamma_1, \Gamma_2, \dots, M_1, M_2$ with $\rho_1 \cong A, \rho_2 \cong B$ so only the Γ_2, X_2, Y_2, M_2 irrep contribute nontrivial factors to $\det \mathcal{B}_g$. Letting $m(\rho)$ denote the multiplicity of the ρ irrep in $|GS\rangle$, we now have proven

$$C_2 |GS\rangle = (-1)^{m(\Gamma_2)+m(X_2)+m(Y_2)+m(M_2)}(-1)^{(L_1L_2-4)N_{occ}/2} |GS\rangle \tag{88}$$

since there are $(L_1L_2 - 4)N_{occ}/2$ pairs of states off the high-symmetry momenta. We can now evaluate the global many-body RSI Δ_1 according to $e^{i\frac{\pi}{2}\hat{N}} C_2 |GS\rangle = e^{i\frac{\pi}{2}\Delta_1} |GS\rangle$:

$$\begin{aligned} e^{i\frac{\pi}{2}\hat{N}} C_2 |GS\rangle &= (-1)^{m(\Gamma_2)+m(X_2)+m(Y_2)+m(M_2)}(-1)^{(L_1L_2-4)N_{occ}/2} e^{i\frac{\pi}{2}L_1L_2N_{occ}} |GS\rangle \\ &= (-1)^{m(\Gamma_2)+m(X_2)+m(Y_2)+m(M_2)} e^{i\frac{\pi}{2}L_1L_2N_{occ}} e^{i\frac{\pi}{2}L_1L_2N_{occ}} |GS\rangle \\ &= (-1)^{m(\Gamma_2)+m(X_2)+m(Y_2)+m(M_2)} |GS\rangle \end{aligned} \tag{89}$$

where we used that L_1 and L_2 must be even for the $X, Y,$ and M points to exist, and that there are $N = N_{occ}L_1L_2$ electrons in the groundstate. We see that the $e^{i\frac{\pi}{2}\hat{N}}$ operator which naturally appears in the definition of the many-body global RSI cancels the size-dependent phase factor. Finally, the band theory invariant $m(\Gamma_2) + m(X_2) + m(Y_2) + m(M_2) = \theta_2 \mod 2$ is called a symmetry indicator and is known to obey $\theta_2 = C \mod 2$ where C is the Chern number[4]. Thus Eq. 89 proves

$$\Delta_1^G = 2C \mod 4 \ . \tag{90}$$

We observe that although the $e^{i\frac{\pi}{2}\hat{N}} C_2$ operator gives $\Delta_1^G \in \mathbb{Z}_4$, only even $\Delta_1^G = 2C \mod 4$ is possible in band insulators.

We now return to the general case of Eq. 85. In the spinless groups, we only need to study the rotations C_n since, as proved in Supplementary Note II D, the RSIs with mirrors and/or time-reversal are obtained by reduction from the rotation groups. To evaluate $\det \mathcal{B}_g$, we consider the possible C_n representations induced from each $\mathbf{k}$ point. We now enumerate the possibilities.

In $p4$, the Γ, M points have $G_\Gamma = G_M = 4$, the X, Y points are interchanged by C_4 and have $G_X, G_Y = 2$, and all other points have $G_\mathbf{k} = 1$, the trivial group. The irrep inductions at the high-symmetry points are

$$\begin{aligned} &\Gamma_1 \downarrow 4 = A, \quad \Gamma_2 \downarrow 4 = B, \quad \Gamma_3 \downarrow 4 = {}^2E, \quad \Gamma_4 \downarrow 4 = {}^1E \\ &M_1 \downarrow 4 = A, \quad M_2 \downarrow 4 = B, \quad M_3 \downarrow 4 = {}^2E, \quad M_4 \downarrow 4 = {}^1E \\ &X_1 \downarrow 4 = A \oplus B, \qquad X_2 \downarrow 4 = {}^1E \oplus {}^2E \end{aligned} \tag{91}$$

and at every non-high-symmetry point, the four C_4 related states induce $A \oplus B \oplus {}^1E \oplus {}^2E$ with $\det(A \oplus B \oplus {}^1E \oplus {}^2E) = (+1)(-1)(-i)(+i) = -1$. Thus we find

$$\begin{aligned} C_4 |GS\rangle &= e^{i\frac{2\pi}{4}(2m(\Gamma_2)+2m(M_2)+m(\Gamma_3)+m(M_3)-m(\Gamma_4)-m(M_4)+2m(X_1))}(-1)^{(L_1L_2-4)N_{occ}/4} |GS\rangle \\ e^{i\frac{\pi}{4}\hat{N}} C_4 |GS\rangle &= e^{i\frac{2\pi}{4}(2m(\Gamma_2)+2m(M_2)+m(\Gamma_3)+m(M_3)-m(\Gamma_4)-m(M_4)+2m(X_1))} e^{i\frac{\pi}{4}(L_1L_2-4)N_{occ}+i\frac{\pi}{4}L_1L_2N_{occ}} |GS\rangle \\ &= e^{i\frac{2\pi}{4}(2m(\Gamma_2)+2m(M_2)+m(\Gamma_3)+m(M_3)-m(\Gamma_4)-m(M_4)+2m(X_1))} e^{i\pi N_{occ}} |GS\rangle \end{aligned} \tag{92}$$

where we used that $L_1L_2 \in 4\mathbb{N}$ for the high-symmetry points to exist. We now also use $N_{occ} = m(X_1) + m(X_2)$ to find $e^{i\frac{2\pi}{4}2m(X_1)}e^{i\pi N_{occ}} = e^{i\frac{2\pi}{4}(2m(X_2))}$ yielding

$$e^{i\frac{\pi}{4}\hat{N}}C_4\,|GS\rangle = e^{i\frac{2\pi}{4}(2m(\Gamma_2)+2m(M_2)+m(\Gamma_3)+m(M_3)-m(\Gamma_4)-m(M_4)+2m(X_2))}\,|GS\rangle = e^{i\frac{2\pi}{4}C}\,|GS\rangle \tag{93}$$

where the relation between the irrep multiplicities and the Chern number was first proved in **??** using the Wilson loop. Thus we obtain

$$\Delta_1^G = 2C \mod 8, \quad \Delta_2^G = C \mod 2 \tag{94}$$

where the second equality follows from $e^{i\pi\Delta_2}\,|GS\rangle = C_4^2\,|GS\rangle = (e^{i\frac{\pi}{4}\hat{N}}C_4)^2\,|GS\rangle$ since $\hat{N}$ is a multiple of 4 on $|GS\rangle$.

We now study $G = p6$ with three high-symmetry points where the irrep inductions are

$$\begin{aligned}
&\Gamma_1 \downarrow 6 = A,\ \Gamma_2 \downarrow 6 = B,\ \Gamma_3 \downarrow 6 = {}^2E_1,\ \Gamma_4 \downarrow 6 = {}^2E_2,\ \Gamma_5 \downarrow 6 = {}^1E_1,\ \Gamma_5 \downarrow 6 = {}^1E_2\\
&K_1 \downarrow 6 = A \oplus B,\ K_2 \downarrow 6 = {}^2E_1 \oplus {}^2E_2,\ K_3 \downarrow 6 = {}^1E_1 \oplus {}^1E_2\\
&M_1 \downarrow 6 = A \oplus {}^1E_1 \oplus {}^2E_1,\ M_2 \downarrow 6 = B \oplus {}^1E_2 \oplus {}^2E_2
\end{aligned} \tag{95}$$

and at every non-high-symmetry point, the six C_6-related states induce $A \oplus B \oplus {}^1E_1 \oplus {}^1E_2 \oplus {}^1E_2 \oplus {}^2E_2$ with $\det(A \oplus B \oplus {}^1E_1 \oplus {}^1E_2 \oplus {}^1E_2 \oplus {}^2E_2) = -1$. We compute

$$\begin{aligned}
C_6\,|GS\rangle &= e^{i\frac{2\pi}{6}(3m(\Gamma_2)+m(\Gamma_4)+2m(\Gamma_5)-2m(\Gamma_3)-m(\Gamma_6)+3m(K_1)-m(K_2)+m(K_3)+3m(M_2))}(-1)^{(L_1L_2-6)N_{occ}/6}\,|GS\rangle\\
e^{i\frac{\pi}{6}\hat{N}}C_6\,|GS\rangle &= e^{i\frac{2\pi}{6}(3m(\Gamma_2)+m(\Gamma_4)+2m(\Gamma_5)-2m(\Gamma_3)-m(\Gamma_6)+3m(K_1)-m(K_2)+m(K_3)+3m(M_2))}e^{i\frac{\pi}{6}(2L_1L_2-6)N_{occ}}\,|GS\rangle
\end{aligned} \tag{96}$$

and now use that L_1L_2 must be a multiple of 6 for the K and M points to exist and that $N_{occ} = m(K_1) + m(K_2) + m(K_3)$. Plugging in yields

$$\begin{aligned}
e^{i\frac{\pi}{6}\hat{N}}C_6\,|GS\rangle &= e^{i\frac{2\pi}{6}(3m(\Gamma_2)+m(\Gamma_4)+2m(\Gamma_5)-2m(\Gamma_3)-m(\Gamma_6)+3m(K_1)-m(K_2)+m(K_3)+3m(M_2))}e^{i\frac{2\pi}{6}3(m(K_1)+m(K_2)+m(K_3))}\,|GS\rangle\\
&= e^{i\frac{2\pi}{6}(3m(\Gamma_2)+m(\Gamma_4)+2m(\Gamma_5)-2m(\Gamma_3)-m(\Gamma_6)+2m(K_2)-2m(K_3)+3m(M_2))}\,|GS\rangle\\
&= e^{i\frac{2\pi}{6}C}\,|GS\rangle
\end{aligned} \tag{97}$$

using the results of **??** in the last line. Thus we obtain

$$\Delta_1^G = 2C \mod 12, \quad \Delta_2^G = C \mod 3 \tag{98}$$

again using $(e^{i\frac{\pi}{6}\hat{N}}C_6)^2\,|GS\rangle = C_6^2\,|GS\rangle$ since $|GS\rangle$ contains a multiple of 6 electrons to obtain the last equality.

Finally, we consider $G = p3$ which has an odd rotation. The irrep reductions are

$$\begin{aligned}
\Gamma_1 \downarrow 6 = A, &\quad \Gamma_2 \downarrow 6 = {}^2E, \quad \Gamma_3 \downarrow 6 = {}^1E\\
K_1 \downarrow 6 = A, &\quad K_2 \downarrow 6 = {}^2E, \quad K_3 \downarrow 6 = {}^1E\\
K_1' \downarrow 6 = A, &\quad K_2' \downarrow 6 = {}^1E, \quad K_3' \downarrow 6 = {}^2E
\end{aligned} \tag{99}$$

and at every non-high-symmetry point, the three C_3 related states induce $A\oplus{}^1E\oplus{}^2E$ which has $\det(A\oplus{}^1E\oplus{}^2E) = +1$. It is now direct to compute

$$C_3\,|GS\rangle = e^{i\frac{2\pi}{3}(m(\Gamma_2)-m(\Gamma_3)+m(K_2)-m(K_3)-m(K_2')+m(K_3'))}\,|GS\rangle = e^{i\frac{2\pi}{3}C}\,|GS\rangle \tag{100}$$

which, using the results of **??**, leads to

$$\Delta_1^G = C \mod 3, \qquad \Delta_2^G = 0 \mod 3 \tag{101}$$

where we used that $e^{i\frac{2\pi}{3}\hat{N}}\,|GS\rangle = e^{i\frac{2\pi}{3}\Delta_2}\,|GS\rangle = +\,|GS\rangle$ since must contain a multiple of 3 electrons. This is because L_1, L_2 must be multiples of 3 for the K points to be defined.

We summarize the results of this section as follows. We have proven

$$\begin{aligned}
\Delta_1^G = 2C \mod 2n, &\qquad \Delta_2^G = C \mod n/2, \qquad G_{\mathbf{x}} = C_n, \quad n \text{ even}\\
\Delta_1^G = C \mod n, &\qquad \Delta_2^G = 0 \mod n, \qquad G_{\mathbf{x}} = C_n, \quad n = 3 \text{ odd}\ .
\end{aligned} \tag{102}$$

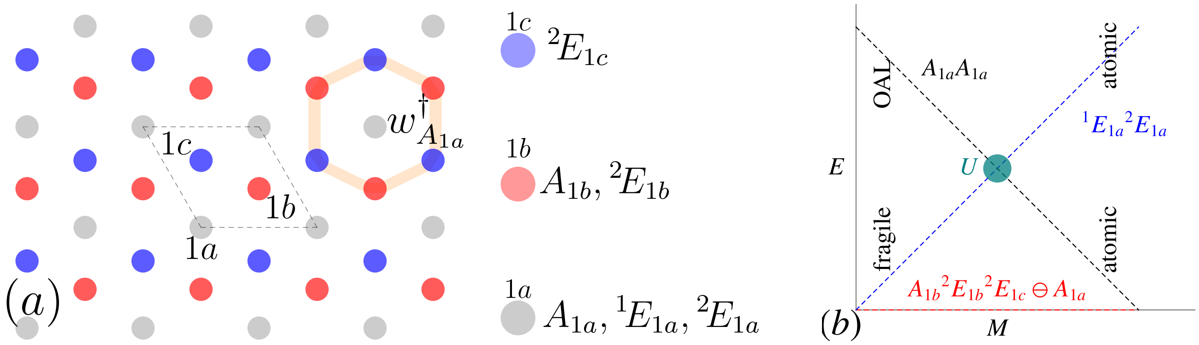

FIG. 7. Supplementary Figure 7. (a) We show the atomic orbitals in the space group $p3$ appearing on the Wyckoff positions 1a (grey), 1b (red), and 1c (blue) which are shown within a dashed unit cell. The fragile bands and OAL complement are formed from the three orbitals at 1b and 1c. The 1a orbitals are decoupled form the rest, and are only acted on by the interaction term. (b) We show the single-particle phase diagram. The fragile bands (red) are fixed at zero energy. The trivial atomic $^1E_{1a}{}^2E_{1a}$ bands (blue) increase in energy as a function of M and the trivial $A_{1a}A_{1a}$ bands (black), one of which is the obstructed atomic Wannier state, decrease in energy. For $M < 1/2$, the $^1E_{1a}{}^2E_{1a}$ do not trivial the fragile topology, but for $M > 1/2$, the $A_{1a}A_{1a}$ bands remove the fragile obstruction. The band crossing is opened by the interaction U.

in a convention where C_n is a rotation about the origin. Because mirrors and time-reversal set $C = 0$ and the many-body global RSIs of general spinless point groups are reduced from those of their rotation subgroups, Eq. 102 completes our classification. With SOC and time-reversal, there is a global many-body RSI Δ_2 which counts the Kramers pairs mod n and is not reduced from the C_n subgroup, but $\Delta_2^G = 0$ is easily proven because all irreps are $2D$ because of spinful time-reversal, and the number of points in the BZ is $L_1 L_2$ which is a multiple of n since we require the high-symmetry points to exist. Thus in all point groups (with and without SOC) with mirrors and/or time-reversal, all the global many-body RSIs vanish on non-interacting states. However, the global many-body RSIs in these point groups do not necessarily vanish on correlated states and therefore diagnose topology which is only possible with strong interactions.

In wallpaper groups with multiple Wyckoff positions, the global many-body RSIs can be evaluated with Eq. 102 and the total many-body momentum, which is zero in a 2D band insulator. For instance in $p4$, consider the Wyckoff positions 1a$= (0,0)$ and 1b$= (1/2,1/2)$ whose point groups are generated by C_4 and T_1C_4, respectively. Because T_1 acts trivially on the filled band groundstate, we have $(\Delta_{1a,1}, \Delta_{1a,2}) = (\Delta_{1b,1}, \Delta_{1b,2}) = (2C, C)$. At the 2c $= \{(1/2,0),(0,1/2)\}$ position, the site symmetry groups are generated by T_1C_2 and T_2C_2 respectively. Evaluating the global many-body RSI at 2c is easily accomplished by computing

$$\begin{aligned} e^{i\frac{\pi}{2}\hat{N}} T_1 C_2 \left|GS, PBC\right\rangle &= (e^{i\frac{\pi}{4}\hat{N}} C_4)^2 T_1 \left|GS, PBC\right\rangle = e^{i2\frac{\pi}{4}\Delta^G_{1a,1}} \left|GS, PBC\right\rangle \\ e^{i\frac{\pi}{2}\hat{N}} T_2 C_2 \left|GS, PBC\right\rangle &= (e^{i\frac{\pi}{4}\hat{N}} C_4)^2 T_2 \left|GS, PBC\right\rangle = e^{i2\frac{\pi}{4}\Delta^G_{1a,1}} \left|GS, PBC\right\rangle \ . \end{aligned} \tag{103}$$

In a general wallpaper group, only the many-body global RSI at the 1a positions needs to be evaluated, and the rest are determined by the many-body translation operators.

V. SUPPLEMENTARY NOTE 5

A. Details of the Exactly Solvable Hamiltonian

In this Supplementary Note, we present a model with single-particle fragile topology that is trivialized by the introduction of interactions. In Supplementary Note V B we construct a non-interacting Hamiltonian with two phases, fragile and trivial, as we compute using single-particle RSIs and band representations. However, we show that the interacting RSIs are trivial in both phases. To illustrate this physically, in Supplementary Note V C we add an interaction term that adiabatically connects the two single-particle phases. We can show this explicitly because the Hamiltonian is exactly solvable.

B. Construction of the Fragile Flat Band Hamiltonian

Out starting point is a non-interacting Hamiltonian on a triangular lattice with 6 bands which has the space group $G = p3$ consisting of translations and a C_3 rotation. The lattice and atomic orbitals are shown in Supplementary Figure 7. We choose $\mathbf{a}_1 = (1,0)/\sqrt{\frac{\sqrt{3}}{2}}, \mathbf{a}_2 = C_3\mathbf{a}_1$ which satisfy $\mathbf{a}_1 \times \mathbf{a}_2 = 1$. The Wyckoff positions are

$$1\text{a} \ = (0,0), \quad 1\text{b} \ = \frac{2}{3}\mathbf{a}_1 + \frac{1}{3}\mathbf{a}_2, \quad 1\text{c} \ = \frac{1}{3}\mathbf{a}_1 + \frac{2}{3}\mathbf{a}_2 \tag{104}$$

and each has PG 3. We choose our model to be spinless and denote the irreps $A, {}^1E, {}^2E$ of PG 3 by

$$D_A[C_3] = 1, \quad D_{^1E}[C_3] = \omega, \quad D_{^2E}[C_3] = \omega^*, \qquad \omega = \exp\left(i\frac{2\pi}{3}\right) \ . \tag{105}$$

We begin the construction of our Hamiltonian by creating the orbitals $A_{1b}, {}^2E_{1b}, {}^2E_{1c}$ where $\rho_{\mathbf{x}}$ denotes the ρ irrep of C_3 at Wyckoff position $\mathbf{x}$. From these orbitals, we construct the following Wannier function at $\mathbf{R} = 0$:

$$w^\dagger_{0,A_{1a}} = \frac{1}{3}\sum_{j=0}^{2} C_3^j (c^\dagger_{0,A_{1b}} + c^\dagger_{0,{}^2E_{1b}} + c^\dagger_{0,{}^2E_{1c}}) C_3^{\dagger j} \tag{106}$$

where C_3 denotes a rotation about the origin. Note that

$$C_3 w^\dagger_{0,A_{1a}} C_3^\dagger = \frac{1}{3}\sum_{j=1}^{3} C_3^j (c^\dagger_{0,A_{1b}} + c^\dagger_{0,{}^2E_{1b}} + c^\dagger_{0,{}^2E_{1c}}) C_3^{\dagger j} = (+1) w^\dagger_{0,A_{1a}} \tag{107}$$

so this state transforms like an A irrep at the $1a$ position, justifying our notation. Secondly, we are justified in calling $w^\dagger_{0,A_{1a}}$ a Wannier function because it obeys the orthogonality condition

$$\{w^\dagger_{\mathbf{R},A_{1a}}, w_{\mathbf{R}',A_{1a}}\} = \delta_{\mathbf{R},\mathbf{R}'} \tag{108}$$

where $w^\dagger_{\mathbf{R},A_{1a}}$ is formed from $w^\dagger_{0,A_{1a}}$ by translation. Eq. 108 may be proved as follows. Clearly $\{w^\dagger_{\mathbf{R},A_{1a}}, w_{\mathbf{R}',A_{1a}}\} = 0$ if $\mathbf{R} \neq \mathbf{R}'$ are *not* nearest neighbors. Let us then compute $\{w^\dagger_{0,A_{1a}}, w_{\mathbf{a}_1,A_{1a}}\}$, which comes from contributions in the $\mathbf{R} = 0$ and $\mathbf{R} = -\mathbf{a}_2$ unit cells. We find

$$\begin{aligned}\{w^\dagger_{0,A_{1a}}, w_{\mathbf{a}_1,A_{1a}}\} &= \frac{1}{9}\Big(\{c^\dagger_{0,A_{1b}} + c^\dagger_{0,{}^2E_{1b}} + {\omega^*}^2 c^\dagger_{-\mathbf{a}_2,{}^2E_{1b}}, c^\dagger_{0,A_{1b}} + \omega c^\dagger_{0,{}^2E_{1b}} + \omega c^\dagger_{-\mathbf{a}_2,{}^2E_{1b}}\}\Big)\\ &= \frac{1}{9}(1+\omega+\omega^*) = 0 \ .\end{aligned} \tag{109}$$

We can check all other anti-commutation relations with other nearest neighbors identically, and we find that they are zero by construction. Invoking translation symmetry, this proves Eq. 108.

We now develop a single-particle Hamiltonian whose bands form $w^\dagger_{\mathbf{R},A_{1a}}$. Performing a Fourier transform, we find the momentum-space eigenvector

$$U_{A_{1a}}(\mathbf{k}) = \frac{1}{3}\begin{pmatrix}1\\1\\1\end{pmatrix} + \frac{1}{3}\begin{pmatrix}1\\ \omega^*\\0\end{pmatrix} e^{-i\mathbf{k}\cdot(-\mathbf{a}_1)} + \frac{1}{3}\begin{pmatrix}1\\ \omega\\ \omega^*\end{pmatrix} e^{-i\mathbf{k}\cdot(-\mathbf{a}_1-\mathbf{a}_2)} + \frac{1}{3}\begin{pmatrix}0\\0\\ \omega\end{pmatrix} e^{-i\mathbf{k}\cdot(-\mathbf{a}_2)} \tag{110}$$

in the basis $(A_{1b}, {}^2E_{1b}, {}^2E_{1c})^T$ which satisfies

$$w^\dagger_{\mathbf{R},A_{1a}} = \int \frac{d^2k}{(2\pi)^2} e^{i\mathbf{R}\cdot\mathbf{k}} U^\alpha_{A_{1a}}(\mathbf{k}) c^\dagger_{\mathbf{k},\alpha} \tag{111}$$

and the sum over $\alpha = A_{1b}, {}^2E_{1b}, {}^2E_{1c}$ is implied. Here $\mathbf{k} = k_1\mathbf{b}_1 + k_2\mathbf{b}_2, \mathbf{b}_i \cdot \mathbf{a}_j = \delta_{ij}$ is a momentum in the Brillouin zone defined by $k_1, k_2 \in (-\pi, \pi)$ and $c^\dagger_{\mathbf{k},\alpha}$ is the electron creation operator at momentum $\mathbf{k}$ at orbital α. If we choose this Wannier band to be a *conduction* band at energy $1 - M > 0$, then the single-particle Hamiltonian is proportional to a projection matrix:

$$(1-M)h_f(\mathbf{k}) = (1-M)U_{A_{1a}}(\mathbf{k})U^\dagger_{A_{1a}}(\mathbf{k}) \tag{112}$$

and thus has three perfectly flat bands. Note that $h_f(\mathbf{k})$ projects out the fragile bands at zero energy, whose orthonormal eigenvectors $U_{f,1}(\mathbf{k}), U_{f,2}(\mathbf{k})$ are orthogonal to $U_{A_{1a}}(\mathbf{k})$. By construction from the $w^\dagger_{\mathbf{R},A_{1a}}$ states, $h_f(\mathbf{k})$ has only nearest neighbor hoppings in real space. The complement of the fragile bands are the obstructed atomic limit band.

Using the Bilbao Crystallographic server (`https://www.cryst.ehu.es/cgi-bin/cryst/programs/bandrep.pl`), we compute the band representation of the unoccupied Wannier band at energy $1-M>0$ to be

$$\Gamma_1 + K_1 + K_1' = A_{1a} \uparrow G \tag{113}$$

as expected by construction. The band representation of the two occupied *valence* bands is

$$2\Gamma_3 + 2K_2 + K_2' + K_3' = (A_{1b} \oplus {}^2E_{1b} \oplus {}^2E_{1c}) \uparrow G \ominus A_{1a} \uparrow G \tag{114}$$

which is single-particle fragile. We expected the occupied bands to have fragile topology because the conduction bands is an *obstructed* atomic limit (OAL), and the difference of two atomic bands can be fragile. Shortly, we show that the valence bands are fragile using their single-particle RSIs.

First, we add three more uncoupled orbitals to the Hamiltonian to be used when we add interactions. We add $A, {}^1E, {}^2E$ orbitals at the $1a$ position, giving 6 orbitals total in the unit cell. In order to preserve the fragile topology of the occupied valence bands, we must put the A_{1a} orbital in the conduction band, and we add the ${}^1E_{1a}, {}^2E_{1a}$ orbitals to the valence band. Thus there are 4 occupied bands and 6 orbitals, so our model is at filling $\nu = 2/3$. In summary, the single-particle Hamiltonian in the basis $A_{1b}, {}^2E_{1b}, {}^2E_{1c}, A_{1a}, {}^1E_{1a}, {}^2E_{1a}$ reads:

$$h_0(\mathbf{k}) = \begin{pmatrix} (1-M)h_f(\mathbf{k}) & & & \\ & 1-M & & \\ & & M & \\ & & & M \end{pmatrix}, \qquad \text{spec } h_0(\mathbf{k}) = \{E(\mathbf{k})\} = \{0,0,M,M,1-M,1-M\} \tag{115}$$

where h_f is a 3×3 matrix and we emphasize that all bands are exactly flat. We show the single particle spectrum in Supplementary Figure 7b. When $M \in (0,1/2)$, the groundstate is fragile at filling 2/3 (we work at fixed particle number). It consists of the two zero energy flat bands, and the M energy trivial ${}^1E_{1a}, {}^2E_{1a}$ bands. The band representation is

$$\mathcal{B}_{val} = \Gamma_2 + 3\Gamma_3 + 3K_2 + K_3 + 2K_2' + 2K_3' = (A_{1b} \oplus {}^2E_{1b} \oplus {}^2E_{1c} \oplus {}^1E_{1a} \oplus {}^2E_{1a}) \uparrow G \ominus A_{1a} \uparrow G \tag{116}$$

which, using the momentum space tables of **??**, gives the non-interacting RSIs

$$\text{fragile:} \qquad \delta_{1a,1} = 2, \delta_{1a,2} = 2, \qquad \delta_{1b,1} = -1, \delta_{1b,2} = 0, \qquad \delta_{1c,1} = 0, \delta_{1c,2} = 1, \qquad M \in [0,1/2)\ . \tag{117}$$

Checking the inequality criteria in **??**, for instance

$$N_{occ} = 4 < \delta_{1a,1} + \delta_{1a,2} - 2\delta_{1b,1} + \delta_{1b,2} + \delta_{1c,1} + \delta_{1c,2} = 2+2+2+1 = 7, \tag{118}$$

we get a further confirmation of the fragile topology of the occupied bands. Note that these RSIs can be directly computed using the Wannier representation $A_{1b} \oplus {}^2E_{1b} \oplus {}^2E_{1c} \ominus A_{1a} \oplus {}^1E_{1a} \oplus {}^2E_{1a}$ and the definitions

$$\delta_{\mathbf{x},1} = -m(A_{\mathbf{x}}) + m({}^1E_{\mathbf{x}}), \quad \delta_{\mathbf{x},2} = -m(A_{\mathbf{x}}) + m({}^2E_{\mathbf{x}}), \qquad \mathbf{x} = 1a,\ 1b,\ 1c\ . \tag{119}$$

At $M = 1/2$, there is a gap closing at $E = 1/2$ where the Hamiltonian undergoes a phase transition. For $M \in (1/2, 1]$, the occupied bands are the two 0 energy fragile bands and the two $1a$ bands at energy $1-M$. Because the fragile bands and their OAL complement are all occupied, this phase is trivial. Technically, an infinitesimally small perturbation to h_f which couples the OAL and fragile bands is needed to mix and trivialize them. This can be confirmed from the single-particle RSIs, which are

$$\text{trivial:} \qquad \delta_{1a,1} = -1, \delta_{1a,2} = -1, \qquad \delta_{1b,1} = -1, \delta_{1b,2} = 0, \qquad \delta_{1c,1} = 0, \delta_{1c,2} = 1, \qquad M \in (1/2, 1] \tag{120}$$

which can be obtained directly from the atomic limit state $A_{1a} \oplus A_{1b} \oplus {}^2E_{1b} \oplus {}^2E_{1c}$ using Eq. 119. Hence it is manifestly trivial.

We will now show that the gap closing at $M = 1/2$ which separates the single-particle phases can be circumvented when interactions are included.

Consulting Supplementary Table VI, we find that the interacting RSIs are the same *in both phases.* Explicitly,

$$(\Delta_{1a,1}, \Delta_{1a,2}) = (1,0), \quad (\Delta_{1b,1}, \Delta_{1b,2}) = (2,1), \quad (\Delta_{1c,1}, \Delta_{1c,2}) = (1,1) \tag{121}$$

Despite the nontrivial single-particle topology, the interacting RSIs are many-body trivial. This is shown using Supplementary Table VIII where the fragile inequality is

$$\text{fragile if: } 4 = N_{occ} < \text{mod}_3(\Delta_{1a,1}|\Delta_{1a,2}) + \text{mod}_3(\Delta_{1b,1}|\Delta_{1b,2}) + \text{mod}_3(\Delta_{1c,1}|\Delta_{1c,2}) = 1+2+1 = 4 \tag{122}$$

which is *false.* Thus the interacting RSIs imply that even single-particle fragile state can be connected to a trivial state. The phase diagram is summarized in Supplementary Figure 8(a). We now show this explicitly by introducing an interaction term that adiabatically connects this state to a trivial atomic limit.

C. Trivialization by Interactions

Before we discuss the interaction term, we will find a convenient expression for the non-interacting Hamiltonian. In momentum space, we have

$$H_0 = \sum_{\mathbf{k}} c^\dagger_{\mathbf{k},\alpha} h^\alpha_\beta(\mathbf{k}) c^\beta_{\mathbf{k}}, \qquad c^\dagger_{\mathbf{k},\alpha} = \frac{1}{\sqrt{\mathcal{N}}} \sum_{\mathbf{R}} e^{-i\mathbf{k}\cdot(\mathbf{R}+\boldsymbol{\delta}_\alpha)} c^\dagger_{\mathbf{R},\alpha} \tag{123}$$

with $\mathcal{N}$ defined as the number of unit cells and the orbital basis is ordered $\alpha = A_{1b}, {}^2E_{1b}, {}^2E_{1c}, A_{1a}, {}^1E_{1a}, {}^2E_{1a}$. In the single-particle eigenbasis, defined by the unitary transformation of the electron operators of the obstructed atomic phase $\gamma^\dagger_{\mathbf{k}} = U^\alpha_{A_{1a}}(\mathbf{k}) c^\dagger_{\mathbf{k},\alpha}$, the Hamiltonian can be rewritten

$$H_0 = \sum_{\mathbf{k}} \left[(1-M)\gamma^\dagger_{\mathbf{k}}\gamma_{\mathbf{k}} + (1-M) c^\dagger_{\mathbf{k},A_{1a}} c_{\mathbf{k},A_{1a}} + M c^\dagger_{\mathbf{k},{}^1E_{1a}} c_{\mathbf{k},{}^1E_{1a}} + M c^\dagger_{\mathbf{k},{}^2E_{1a}} c_{\mathbf{k},{}^2E_{1a}} \right] . \tag{124}$$

Importantly, the electron operators defining the fragile band do not appear because they are at exactly zero energy. Taking $\mathcal{N} \to \infty$, we now perform another change of basis to the Wannier basis defined by

$$w^\dagger_{\mathbf{R},n} = \int \frac{d^2k}{(2\pi)^2} e^{i\mathbf{R}\cdot\mathbf{k}} U^\alpha_n(\mathbf{k}) c^\dagger_{\mathbf{k},\alpha}, \qquad \sum_{\mathbf{R}} e^{-i\mathbf{R}\cdot\mathbf{k}} w^\dagger_{\mathbf{R},n} = U^\alpha_n(\mathbf{k}) c^\dagger_{\mathbf{k},\alpha} \tag{125}$$

where $U^\alpha_n(\mathbf{k})$ are the eigenvectors of the nth band. For the three uncoupled 1a orbitals, the eigenvectors are trivial: $U^\alpha_n(\mathbf{k})$, and the Wannier basis is simply the orbital basis. Because the bands are perfectly flat, we can solve the Fourier transforms and find

$$H_0 = \sum_{\mathbf{R}} \left[(1-M) w^\dagger_{\mathbf{R},A_{1a}} w_{\mathbf{R},A_{1a}} + (1-M) c^\dagger_{\mathbf{R},A_{1a}} c_{\mathbf{R},A_{1a}} + M c^\dagger_{\mathbf{R},{}^1E_{1a}} c_{\mathbf{R},{}^1E_{1a}} + M c^\dagger_{\mathbf{R},{}^2E_{1a}} c_{\mathbf{R},{}^2E_{1a}} \right] . \tag{126}$$

The Wannier basis has diagonalized the Hamiltonian in energy *and* in the local unit cell. Recall from Eq. 106 that $w^\dagger_{\mathbf{R},A_{1a}}$ the sum electron operators on the 6 nearest neighbor sites. Hence all operators in H_0 are local.

Inspired by the simple form of Eq. 126, we now add in the interaction term

$$H_{int} = U \sum_{\mathbf{R}} w^\dagger_{\mathbf{R},A_{1a}} c^\dagger_{\mathbf{R},A_{1a}} c_{\mathbf{R},{}^1E_{1a}} c_{\mathbf{R},{}^2E_{1a}} + h.c. \tag{127}$$

which introduces scattering between the ${}^1E, {}^2E$ irreps and A, A irreps at 1a. Notably, the interacting involves pair-hopping because the $w^\dagger_{A_{1a}}$ Wannier state is supported off the 1a position. First, we verify that H_{int} is local because $w^\dagger_{\mathbf{R},A_{1a}}$ is local. Second, we check that H_{int} preserves the symmetries of H_0. By construction, H_{int} is invariant under translations. To show that $C_3^\dagger H_{int} C_3 = H_{int}$, we recall

$$\begin{aligned}
C_3^\dagger w^\dagger_{\mathbf{R},A_{1a}} C_3 &= w^\dagger_{C_3\mathbf{R},A_{1a}} \\
C_3^\dagger c^\dagger_{\mathbf{R},A_{1a}} C_3 &= c^\dagger_{C_3\mathbf{R},A_{1a}} \\
C_3^\dagger c^\dagger_{\mathbf{R},{}^1E_{1a}} C_3 &= \omega c_{C_3\mathbf{R},{}^1E_{1a}} \\
C_3^\dagger c^\dagger_{\mathbf{R},{}^2E_{1a}} C_3 &= \omega^* c_{C_3\mathbf{R},{}^2E_{1a}}
\end{aligned} \tag{128}$$

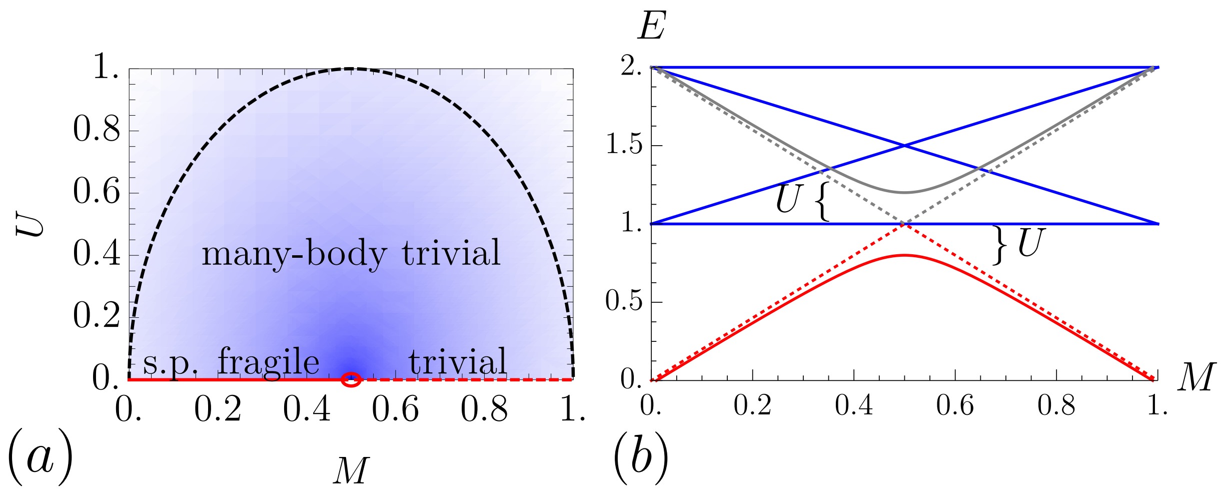

FIG. 8. Supplementary Figure 8. (a) We sketch the phase diagram of $H = H_0 + H_{int}$. At $U = 0$, the single-particle (s.p.) fragile phase is denoted as a solid red line. A gap closing (open oval) at $M = 1/2, U = 0$ separates this phase from the trivial atomic limit, denoted by a dashed red line. However, if $U \neq 0$, the single-particle classifications are destroyed and the model is many-body trivial. In particular, the black dashed line Eq. 135 connects the two single-particle phases without a gap closing, showing that interactions trivialize the single-particle fragile topolgy. (b) H is exactly solvable because it is decoupled in the Wannier basis. We show the spectrum of H in a single unit cell determined in Eq. 134. The groundstate is shown in solid red for nonzero U and dashed for $U = 0$. When $U = 0$, we see there is a gap closing with a higher band at $M = 1/2$ as expected from the single-particle physics.

from which we compute

$$C_3^\dagger H_{int} C_3 = U \sum_{\mathbf{R}} w^\dagger_{C_3\mathbf{R},A_{1a}} c^\dagger_{C_3\mathbf{R},A_{1a}} \omega\omega^* c_{C_3\mathbf{R},{}^1E_{1a}} c_{C_3\mathbf{R},{}^2E_{1a}} + h.c. = H_{int} \ . \tag{129}$$

In summary, we have shown that

$$\begin{aligned} H = H_0 + H_{int} = \sum_{\mathbf{R}} \Big[&(1-M)(w^\dagger_{\mathbf{R},A_{1a}} w_{\mathbf{R},A_{1a}} + c^\dagger_{\mathbf{R},A_{1a}} c_{\mathbf{R},A_{1a}}) + M(c^\dagger_{\mathbf{R},{}^1E_{1a}} c_{\mathbf{R},{}^1E_{1a}} + c^\dagger_{\mathbf{R},{}^2E_{1a}} c_{\mathbf{R},{}^2E_{1a}}) \\ &+ U w^\dagger_{\mathbf{R},A_{1a}} c^\dagger_{\mathbf{R},A_{1a}} c_{\mathbf{R},{}^1E_{1a}} c_{\mathbf{R},{}^2E_{1a}} + h.c. \Big] \end{aligned} \tag{130}$$

is a fully local Hamiltonian with $G = p3$ *and* is diagonalized in the Wannier basis, e.g. each term in the $\mathbf{R}$ sum commutes with every other. H can be interpreted as a “stabilizer code” in the Wannier basis.

D. Exact Solution of H

We now solve H exactly by exploiting its diagonalization in Wannier space. In a given unit cell at $\nu = 4/6$, the Hilbert space is $\binom{6}{4}$-dimensional and is spanned by all 4-operator products chosen from the set $w^\dagger_{\mathbf{R},A_{1a}}, c^\dagger_{\mathbf{R},A_{1a}}, c^\dagger_{\mathbf{R},{}^1E_{1a}}, c^\dagger_{\mathbf{R},{}^2E_{1a}}, f^\dagger_{\mathbf{R},1},$ Here $f^\dagger_{\mathbf{R},1}, f^\dagger_{\mathbf{R},2}$ denote the electron operators of the fragile band in the “Wannier” basis defined by

$$f^\dagger_{\mathbf{R},j} = \int \frac{d^2k}{(2\pi)^2} e^{i\mathbf{R}\cdot\mathbf{k}} U^\alpha_{f,j}(\mathbf{k}) c^\dagger_{\mathbf{k},\alpha}, \qquad j = 1,2 \tag{131}$$

where $U_{f,j}(\mathbf{k})$ are the single-particle eigenvectors defined below Eq. 112 and satisfy $U^\dagger_{f,j}(\mathbf{k}) U_{A_{1a}}(\mathbf{k}) = 0$. Because of the single-particle fragile topology, there is no choice of $U_{f,j}(\mathbf{k})$ where $f^\dagger_{\mathbf{R},j}$ are transform local under the symmetries and are exponentially decaying. For concreteness, we pick $U_{f,j}(\mathbf{k})$ such that $f^\dagger_{\mathbf{R},j}$ *are* exponentially localized[10], at the expense of a local action of the symmetries on $f^\dagger_{\mathbf{R},j}$. Most importantly, $f^\dagger_{\mathbf{R},j}$ and $w^\dagger_{\mathbf{R},A_{1a}}$ form a complete orthonormal basis of the Hilbert space at $\mathbf{R}$. Moreover, the fragile bands are at zero energy and are annihilated by H. Finally, we emphasize that the operators $w^\dagger_{\mathbf{R},A_{1a}}, c^\dagger_{\mathbf{R},A_{1a}}, c^\dagger_{\mathbf{R},{}^1E_{1a}}, c^\dagger_{\mathbf{R},{}^2E_{1a}}, f^\dagger_{\mathbf{R},1}, f^\dagger_{\mathbf{R},2}$ are all orthonormal. The operators

$w^\dagger_{\mathbf{R},A_{1a}}, c^\dagger_{\mathbf{R},A_{1a}}, c^\dagger_{\mathbf{R},{}^1E_{1a}}, c^\dagger_{\mathbf{R},{}^2E_{1a}}$ are local (and compactly supported). The operators $f^\dagger_{\mathbf{R},1}, f^\dagger_{\mathbf{R},2}$ are non-local but do not appear in the Hamiltonian:

$$[H, f^\dagger_{\mathbf{R},1}] = [H, f^\dagger_{\mathbf{R},2}] = 0 \ . \tag{132}$$

Thus H is manifestly local. (It has mutually commuting local terms.)

Writing H explicitly in the 15-dimensional local Hilbert space, we find that H is diagonal except for a U-coupling between the four-particle states $f^\dagger_{\mathbf{R},1}f^\dagger_{\mathbf{R},2}w^\dagger_{\mathbf{R},A_{1a}}c^\dagger_{\mathbf{R},A_{1a}}$ and $f^\dagger_{\mathbf{R},1}f^\dagger_{\mathbf{R},2}c^\dagger_{\mathbf{R},{}^1E_{1a}}c^\dagger_{\mathbf{R},{}^2E_{1a}}$. This is guaranteed by the orthogonality of the operators. Nonzero U mixes these states, which we can think of as performing the adiabatic process $AA \to {}^1E^2E$ at the 1a site. This process is the one responsible for trivializing the single-particle state, e.g.

$$A_{1b} \oplus {}^2E_{1b} \oplus {}^2E_{1c} \ominus A_{1a} \oplus {}^1E_{1a} \oplus {}^2E_{1a} \to A_{1b} \oplus {}^2E_{1b} \oplus {}^2E_{1c} \ominus A_{1a} \oplus A_{1a} \oplus A_{1a} = A_{1b} \oplus {}^2E_{1b} \oplus {}^2E_{1c} \oplus A_{1a} \tag{133}$$

where we see that the Wannier obstruction has been removed.

We now show this explicitly. The spectrum of H in the local Hilbert space is (dropping the unit cell subscript for clarity)

$$\begin{aligned}
\frac{1}{\sqrt{(E_+-2M)^2+U^2}}\left((E-2M)f_1^\dagger f_2^\dagger w^\dagger_{A_{1a}}c^\dagger_{A_{1a}} + U f_1^\dagger f_2^\dagger c^\dagger_{{}^1E_{1a}}c^\dagger_{{}^2E_{1a}}\right)|0\rangle \quad & E_+ = 1+\sqrt{(1-2M)^2+U^2} \\
\left(w^\dagger_{A_{1a}}c^\dagger_{A_{1a}}c^\dagger_{{}^1E_{1a}}c^\dagger_{{}^2E_{1a}}\right)|0\rangle : \quad & E = 2 \\
f_1^\dagger f_2^\dagger c^\dagger_{A_{1a}}c^\dagger_{{}^2E_{1a}}|0\rangle, f_1^\dagger f_2^\dagger c^\dagger_{A_{1a}}c^\dagger_{{}^1E_{1a}}|0\rangle, f_1^\dagger f_2^\dagger w^\dagger_{A_{1a}}c^\dagger_{{}^2E_{1a}}|0\rangle, f_1^\dagger f_2^\dagger w^\dagger_{A_{1a}}c^\dagger_{{}^1E_{1a}}|0\rangle : \quad & E = 1 \\
w^\dagger_{A_{1a}}c^\dagger_{A_{1a}}c^\dagger_{{}^1E_{1a}}f_1^\dagger|0\rangle, w^\dagger_{A_{1a}}c^\dagger_{A_{1a}}c^\dagger_{{}^1E_{1a}}f_2^\dagger|0\rangle, w^\dagger_{A_{1a}}c^\dagger_{A_{1a}}c^\dagger_{{}^2E_{1a}}f_1^\dagger|0\rangle, w^\dagger_{A_{1a}}c^\dagger_{A_{1a}}c^\dagger_{{}^2E_{1a}}f_2^\dagger|0\rangle : \quad & E = 2-M \\
c^\dagger_{{}^1E_{1a}}c^\dagger_{{}^2E_{1a}}f_1^\dagger c^\dagger_{A_{1a}}|0\rangle, c^\dagger_{{}^1E_{1a}}c^\dagger_{{}^2E_{1a}}f_2^\dagger c^\dagger_{A_{1a}}|0\rangle, c^\dagger_{{}^1E_{1a}}c^\dagger_{{}^2E_{1a}}f_1^\dagger w^\dagger_{A_{1a}}|0\rangle, c^\dagger_{{}^1E_{1a}}c^\dagger_{{}^2E_{1a}}f_2^\dagger w^\dagger_{A_{1a}}|0\rangle : \quad & E = 1+M \\
\frac{1}{\sqrt{(E_--2M)^2+U^2}}\left((E_--2M)f_1^\dagger f_2^\dagger w^\dagger_{A_{1a}}c^\dagger_{A_{1a}} + U f_1^\dagger f_2^\dagger c^\dagger_{{}^1E_{1a}}c^\dagger_{{}^2E_{1a}}\right)|0\rangle \quad & E_- = 1-\sqrt{(1-2M)^2+U^2} \ .
\end{aligned} \tag{134}$$

The spectrum is plotted as a function of M in Supplementary Figure 8(b). The last state is the unique groundstate everywhere except $M = 1/2, U = 0$. We can see that the interaction as coupled the two groundstates of the two single-particle phases ($f_1^\dagger f_2^\dagger c^\dagger_{{}^1E_{1a}}c^\dagger_{{}^2E_{1a}}$ which is fragile and $f_1^\dagger f_2^\dagger w^\dagger_{A_{1a}}c^\dagger_{A_{1a}}$ which is trivial) and has opened the gap at $M = 1/2$ for nonzero U. In fact, the gap is given by $\sqrt{(1-2M)^2+U^2}$ and is nonzero everywhere except $M = 1/2, U = 0$ where there is a six-fold degeneracy.

For concreteness, we can choose a parameterization in the U, M phase diagram that connects the two single-particle regions where $U = 0$ without closing the many-body gap. We take

$$M = \frac{1}{2}(1-\cos\theta), \quad U = \sin\theta, \qquad \theta \in (0,\pi) \tag{135}$$

which tunes between $M = 0, U = 0$ and $M = 1, U = 0$. The full many-body groundstate along this path is given by

$$|GS(\theta)\rangle = \prod_{\mathbf{R}} \left(\cos\frac{\theta}{2} f^\dagger_{\mathbf{R},1}f^\dagger_{\mathbf{R},2}c^\dagger_{\mathbf{R},{}^1E_{1a}}c^\dagger_{\mathbf{R},{}^2E_{1a}} - \sin\frac{\theta}{2} f^\dagger_{\mathbf{R},1}f^\dagger_{\mathbf{R},2}w^\dagger_{\mathbf{R},A_{1a}}c^\dagger_{\mathbf{R},A_{1a}}\right)|0\rangle \tag{136}$$

and the many-body gap $\sqrt{(1-2M)^2+U^2}$ is equal to 1 for all θ. At $\theta = \pi$, the groundstate is a Slater determinant and hence

$$|GS(\pi)\rangle = \prod_{\mathbf{R}} \left(-f^\dagger_{\mathbf{R},1}f^\dagger_{\mathbf{R},2}w^\dagger_{\mathbf{R},A_{1a}}c^\dagger_{\mathbf{R},A_{1a}}\right)|0\rangle \propto \prod_{\mathbf{R}} c^\dagger_{\mathbf{R},A_{1b}}c^\dagger_{\mathbf{R},{}^2E_{1b}}c^\dagger_{\mathbf{R},{}^2E_{1c}}c^\dagger_{\mathbf{R},A_{1a}}|0\rangle \tag{137}$$

because $f^\dagger_{\mathbf{R},1}, f^\dagger_{\mathbf{R},2}, w^\dagger_{\mathbf{R}A_{1a}}$ form a complete basis of the $A_{1b}, {}^2E_{1b}, {}^2E_{1c}$ orbitals, and hence by total anti-symmetry it follows that

$$\prod_{\mathbf{R}} f^\dagger_{\mathbf{R},1}f^\dagger_{\mathbf{R},2}w^\dagger_{\mathbf{R},A_{1a}} = e^{i\varphi}\prod_{\mathbf{R}} c^\dagger_{\mathbf{R},A_{1b}}c^\dagger_{\mathbf{R},{}^2E_{1b}}c^\dagger_{\mathbf{R},{}^2E_{1c}} \tag{138}$$

where $e^{i\varphi}$ is the determinant of the unitary operator that changes the orbital basis to the Wannier basis in Eqs. 125 and 131. The righthand side of Eq. 138 makes it clear that $|GS(\pi)\rangle$ is a trivial atomic limit.

---

[1] MI Aroyo, JM Perez-Mato, Cesar Capillas, Eli Kroumova, Svetoslav Ivantchev, Gotzon Madariaga, Asen Kirov, and Hans Wondratschek. Bilbao crystallographic server: I. databases and crystallographic computing programs. *ZEITSCHRIFT FUR KRISTALLOGRAPHIE*, 221:15–27, 01 2006. doi:10.1524/zkri.2006.221.1.15.

[2] Mois I. Aroyo, Asen Kirov, Cesar Capillas, J. M. Perez-Mato, and Hans Wondratschek. Bilbao Crystallographic Server. II. Representations of crystallographic point groups and space groups. *Acta Crystallographica Section A*, 62(2):115–128, Mar 2006. doi:10.1107/S0108767305040286. URL `https://doi.org/10.1107/S0108767305040286`.

[3] Zhi-Da Song, Luis Elcoro, and B. Andrei Bernevig. Twisted bulk-boundary correspondence of fragile topology. *Science*, 367 (6479):794–797, February 2020. doi:10.1126/science.aaz7650.

[4] Chen Fang, Matthew J. Gilbert, and B. Andrei Bernevig. Bulk topological invariants in noninteracting point group symmetric insulators. *Phys. Rev. B*, 86(11):115112, September 2012. doi:10.1103/PhysRevB.86.115112.

[5] Shang Liu, Ashvin Vishwanath, and Eslam Khalaf. Shift Insulators: Rotation-Protected Two-Dimensional Topological Crystalline Insulators. *Physical Review X*, 9(3):031003, July 2019. doi:10.1103/PhysRevX.9.031003.

[6] Zhi-Da Song, L. Elcoro, Nicolas Regnault, and B. Andrei Bernevig. Fragile Phases As Affine Monoids: Full Classification and Material Examples. *arXiv e-prints*, art. arXiv:1905.03262, May 2019.

[7] Benjamin J. Wieder and B. Andrei Bernevig. The Axion Insulator as a Pump of Fragile Topology. *arXiv e-prints*, art. arXiv:1810.02373, Oct 2018.

[8] Jonah Herzog-Arbeitman, Valerio Peri, Frank Schindler, Sebastian D. Huber, and B. Andrei Bernevig. Superfluid Weight Bounds from Symmetry and Quantum Geometry in Flat Bands. *Phys. Rev. Lett.*, 128(8):087002, February 2022. doi: 10.1103/PhysRevLett.128.087002.

[9] B. Andrei Bernevig and Taylor L. Hughes. *Topological Insulators and Topological Superconductors*. Princeton University Press, student edition edition, 2013. ISBN 9780691151755. URL `http://www.jstor.org/stable/j.ctt19cc2gc`.

[10] Christian Brouder, Gianluca Panati, Matteo Calandra, Christophe Mourougane, and Nicola Marzari. Exponential localization of wannier functions in insulators. *Phys. Rev. Lett.*, 98:046402, Jan 2007. doi:10.1103/PhysRevLett.98.046402. URL `https://link.aps.org/doi/10.1103/PhysRevLett.98.046402`.
